# Ligand-Assisted Formation of Heterobimetallic Adducts of AlMe_3_ and ZnMe_2_ with Zr and Hf Salan Catalysts for Olefin Polymerization

**DOI:** 10.1021/acs.inorgchem.5c03732

**Published:** 2025-11-27

**Authors:** Martina Morello, Anna Dall’Anese, Luca Rocchigiani, Christian Ehm, Roberta Cipullo, Pavel S. Kulyabin, Dmitry V. Uborsky, Alexander Z. Voskoboynikov, Alceo Macchioni, Vincenzo Busico, Cristiano Zuccaccia

**Affiliations:** † Department of Industrial Engineering, University of Padua, via Gradenigo 6/a, 35131 Padova, Italy; ‡ Department of Chemistry, Biology and Biotechnology and CIRCC, University of Perugia, via Elce di Sotto 8, 06123 Perugia, Italy; § Department of Chemical Sciences, Federico II University of Naples, via Cinthia, 80126 Napoli, Italy; ∥ Department of Chemistry, Lomonosov Moscow State University, 1/3 Leninskie Gory, 119991 Moscow, Russia; ⊥ DPI, P.O. Box 902, 5600 AX Eindhoven, The Netherlands

## Abstract

Cationic Salan Zr- and Hf-benzyl precatalysts for olefin polymerization were reacted with AlMe_3_ and ZnMe_2_ to probe their tendency to form stable heterobimetallic adducts and understand the role played by the ligand-based Lewis-basic functionalities in modulating the nature of such adducts. NMR studies in solution and DFT computations show that unlike typical metallocenes, Salan complexes bind AlMe_3_ and ZnMe_2_ to form only a single μ-Me interaction between the transition and the main-group metal. The latter is engaged in a further interaction with one oxygen atom of the ligand, leading to an unsymmetrical adduct. The formed heterobimetallic complexes are fluxional in solution, and their chemical exchange patterns were characterized by ^1^H EXSY NMR. In the case of AlMe_3_, exchange between bridging and terminal Al–Me moieties occurs preferentially via an intramolecular mechanism without the involvement of external AlMe_3_. In stark contrast, both bridging and terminal Zn–Me groups undergo chemical exchange with external ZnMe_2_ in the corresponding heterobimetallic adduct. Quantification of the activation parameters for Hf/ZnMe_2_ systems suggests a dissociative mechanism for the exchange involving the bridging methyl and an associative mechanism for the terminal methyl.

## Graphical abstract

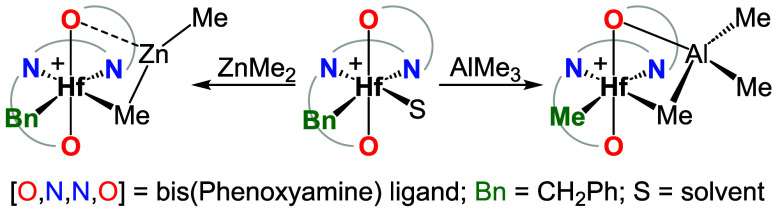

## Introduction

Heterometallic species derived from the combination of group IV transition metal complexes and main-group metal alkyl, hydride, or halide compounds mediate a number of stoichiometric or catalytic transformations,^1^ including metalation of unsaturated substrates,^2–6^ oligomerization,^7–11^ and polymerization of olefins.^12–17^ In the latter case, the formation of multimetallic adducts is the key for the activation^18^ of industrially relevant dichloride precursors by means of methylaluminoxane (MAO),^19–21^ molecular analogues of MAO,^22–25^ or a combination of trialkyl aluminum and Lewis or Brønsted acidic organic cations paired with weakly coordinating perfluoroborate anions.^26,27^

The association between an organotransition catalyst and a main-group metal alkyl is also important for enabling Chain Shuttling Polymerization (CSP)^28–31^ and Coordinative Chain Transfer Polymerization (CCTP),^32–37^ which are remarkable technologies for the production of specialty polyolefins. Cationic four-membered heterobimetallic species held together by two μ-alkyl groups (**I**, Chart 1) are proposed as the key CSP/CCTP intermediates, as they mediate the transfer of the growing polymer chain between the active metal center and the inactive main-group metal (chain transfer agent, CTA).^38–42^ Support for this proposal comes from the spectroscopic^43–47^ or crystallographic^48,49^ characterization of species featuring simple methyl bridges, and, more rarely, of systems bearing bridging alkyl chains or with inequivalent bridging alkyls.^50–52^ As an example, some of us recently reported cationic metallocene Hf/Al and Hf/Zn heterobimetallic complexes with inequivalent bridging alkyls: structural characterization and dynamic behavior in solution highlighted the crucial role of α-agostic interactions^53^ and the impact of the alkyl chain nature in modulating thermodynamics and kinetics of the formation/breaking of these adducts.^54^ Only a relatively small number of olefin polymerization catalysts are suitable for CSP or CCTP owing to the stringent kinetic requirements of rapid (with respect to propagation) and reversible trans-alkylation in the presence of a CTA.^55^ For instance, recent studies demonstrated that typical metallocene catalysts are unable to mediate the production of olefin block copolymer (OBC) via CCTP,^56^ whereas Sita’s Hf-based half metallocene,^57–59^ Symyx/Dow Hf-pyridyl-amido post-metallocenes,^60–66^ Mitsui FI catalysts,^67,68^ and Gibson’s Fe-based complex^38,39^ are capable of promoting CCTP. Leaving out steric and electronic properties, the presence of ancillary ligands with heteroatom functionalities (O, N) in proximity of the active site is a general feature of a working CCTP catalyst. This raises the question of whether an alternative interaction modality between the active site and the CTA (**II**, Chart 1) involving an interaction with the O- or N-functionalities of the ligand and facilitating trans-alkylation, as recently proposed by Sita, is active.^69^ To the best of our knowledge, the only structural characterization in a solution of species of type **II** was reported by Mountford and Clot for titanium imido methyl complexes;^70^ attempts with other systems have been unsuccessful so far.^71^

**Chart 1:**
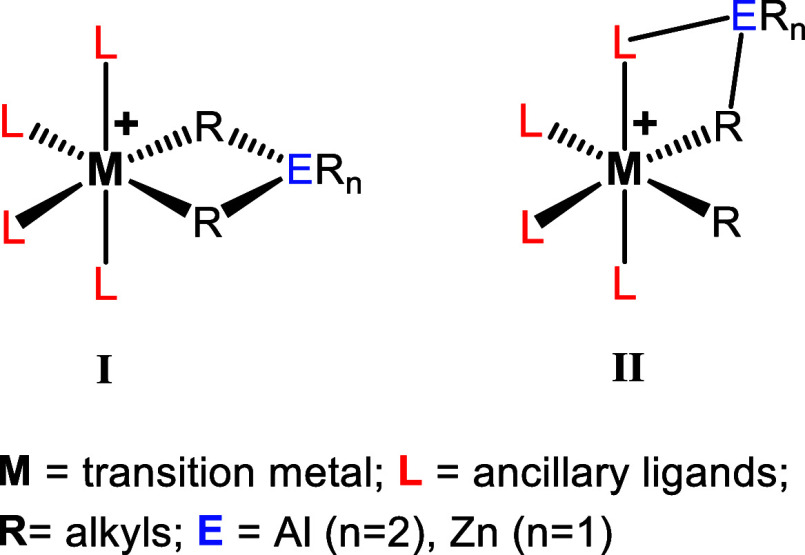
Typical Heterobimetallic Complexes Featuring Two Bridging μ-Alkyls (**I**) and the Alternative Structure (**II**) Featuring a Bridge Involving Ligand Functionalities

Over the last years, we investigated structure–activity relationships for the class of phenoxy-amine (Salan-type) post-metallocene olefin polymerization catalysts,^72–77^ evidencing the main factors determining the structure and stability of the catalyst resting-state and, consequently, catalyst activity. The latter is modulated by the dynamic catalyst speciation between poorly active *mer*-*mer* and highly active *fac*-*fac* isomers,^78,79^ whose ratio can be tuned by the choice of the phenolate ring substituents and solvent,^80^ or by the proper stiffening of the ligand backbone.^81^ Cationic species derived from Salan precatalysts have rather good thermal stability and possess heteroatom functionalities at the active site, so they appear to be optimal candidates for investigating the role played by such donor atoms in the first coordination sphere of the metal in modulating the interaction with aluminum and zinc alkyls CTAs.

Here, we report the results of NMR and DFT investigations of the heterobimetallic adducts derived from the reaction of hafnium complexes bearing [ONNO] Salan ligands and AlMe_3_ or ZnMe_2_. They provide compelling evidence that the presence of oxygen functionalities in the ligand framework controls the modality of the interaction between the cationic metal center and the chain transfer agent, while the nature of the CTA affects the fluxionality pattern of these species.

## Results and Discussion

### Generation and Characterization of Cationic Monobenzyl Complexes

Hafnium cationic monobenzyl derivatives bearing different substituents (R) in the phenoxy ring (**2Hf**_**a**_-**2Hf**_**d**_, Scheme 1) were generated in situ by the reaction of the corresponding neutral dibenzyl precursor (**1Hf**_**a**_ **-1Hf**_**d**_), with 1 equiv of [CPh_3_]­[B­(C_6_F_5_)_4_] (TTB) in C_6_D_5_Cl, as previously reported for the analogous zirconium complexes (**2Zr**_**a**_-**2Zr**_**d**_, Scheme 1).^80^ The new cationic complexes were fully characterized in solution by multinuclear and multidimensional NMR spectroscopy (NMR data are given in the Supporting Information).

**Scheme 1:**
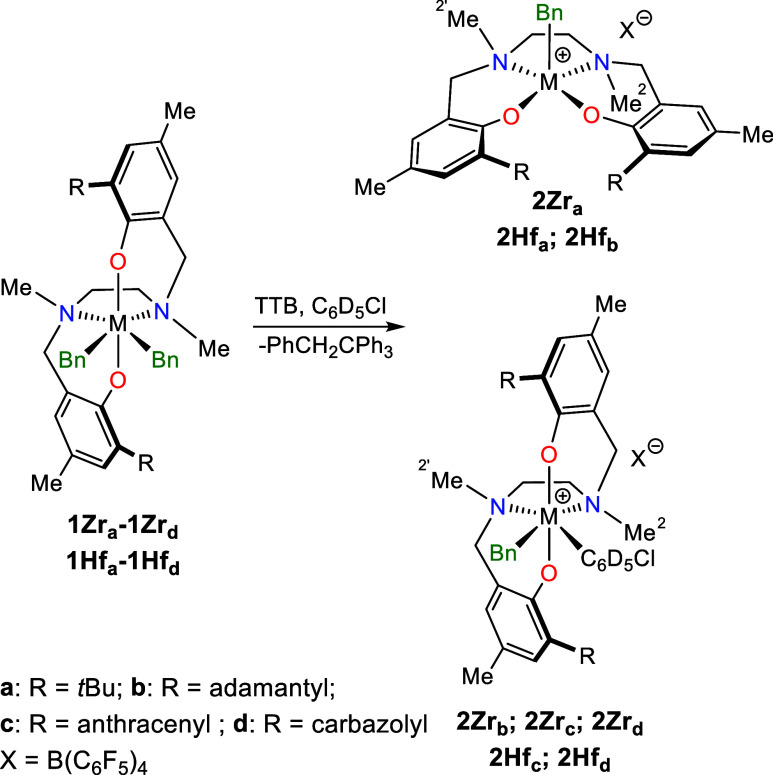
Activation of the Dibenzyl Neutral Precursor with TTB Affording the Corresponding Cationic Complexes Having *mer-mer* (Top) or *fac-fac* (Bottom) Geometries

As recently reported,^80^ the coordination geometry of the [ONNO] ligand around the metal can be easily determined by monitoring the diagnostic ^13^C NMR chemical shift values of the two NMe moieties (C^2^ and C^2′^, Scheme 1). If both C^2^ and C^2′^ resonate at δ_C_ > 40 ppm, the geometry is *fac-fac*: this occurs either in the neutral starting complexes and in cations^80^ where a solvent molecule completes the coordination sphere of the metal.^82–85^ On the contrary, for systems preferring the five-coordinated *mer-mer* geometry, the chemical shift of C^2^ is always observed at δ_C_ < 40 ppm. The preferred coordination mode can be confirmed by measuring the activation entropy value (Δ*S*_SE_^‡^) of the Site Epimerization (SE) process, namely, the dynamic inversion of the configuration at the metal center, leading to magnetization exchange between the corresponding resonances of the two halves of the ligand.^86–95^ Positive values of Δ*S*_SE_^‡^ are observed for *fac-fac* geometries, since the (partial) displacement of the coordinated solvent is necessary to trigger SE. In contrast, negative values of Δ*S*_SE_^‡^ are found for *mer-mer* systems, as the association of a solvent molecule is required to initiate the SE process.

For **2Hf**_**a**_, δ_C2_ and δ_C2′_ are observed at 37.7 and 42.9 ppm, respectively, and Δ*S*_SE_^‡^ is negative (Supporting Information and Table S1), indicating that **2Hf**_**a**_ adopts a *mer*-*mer* geometry in solution (Scheme 1, top right). For **2Hf**_**c**_ and **2Hf**_**d**_, both δ_C2_ and δ_C2′_ are found at frequencies higher than 40 ppm and Δ*S*_SE_^‡^ is positive, in agreement with the *fac*-*fac* geometry (Scheme 1, bottom right).^80^ Those findings mirror the behavior of the corresponding zirconium derivatives. Instead, a switch of geometry is observed for systems bearing the bulky adamantyl substituent depending on the nature of the metal center: **2Zr**_**b**_ is present in solution as a *fac*-*fac* isomer, whereas the *mer*-*mer* geometry is preferred for **2Hf**_**b**_ (δ_C2_ = 38.3 ppm; δ_C2′_ = 42.1 ppm; Δ*S*_SE_^‡^ = −7 cal K^–1^ mol^–1^). The reason for this difference is unclear at present.

### Reaction of Complexes **2** with AlMe_3_

The reaction of **2Hf**_**a**_–**2Hf**_**d**_ with an excess of AlMe_3_ in C_6_D_5_Cl proceeds smoothly at room temperature, affording, over the course of a few minutes, the corresponding cationic heterobimetallic adducts (**3Hf**_**a**_–**3Hf**_**d**_, Scheme 2) together with one equivalent of BnAlMe_2_. The latter was easily recognized by the shift of the original M-*C*H_2_Ph ^13^C NMR resonance from δ_C_ ≈ 60–80 ppm in complexes **2** to δ_C_ ≈ 25 ppm, a typical value for the Al-*C*H_2_Ph moiety.^96^

**Scheme 2:**
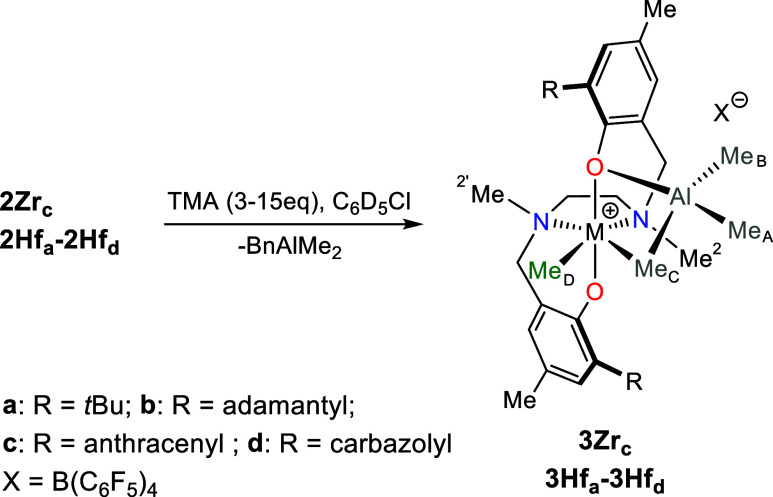
Reaction of Complexes **2** with AlMe_3_ Affording Complexes **3** with the *fac-fac* Geometry

Despite several attempts, using various excess quantities of AlMe_3_ and different temperatures, the reaction of both **2Zr**_**a**_ and **2Zr**_**b**_ always resulted in a complex mixture of species (Figures S21 and S22 in the Supporting Information). Although inspection of NMR spectra allowed us to obtain some evidence about the formation of the corresponding heterobimetallic adducts **3Zr**_**a**_ and **3Zr**_**b**_, their complete NMR characterization was not possible. Under the same conditions, **2Zr**_**c**_ incompletely reacts with AlMe_3_ to afford a mixture of **2Zr**_**c**_ and **3Zr**_**c**_ (Figure S23 in the Supporting Information). The reaction of **2Zr**_**d**_ with AlMe_3_ was not attempted.

Identification and structural characterization in solution of **3Zr**_**c**_ and **3Hf**_**a**_ **-3Hf**_**d**_ was carried out by means of low-temperature multinuclear and multidimensional NMR spectroscopy, since the decomposition to unidentified species occurs at room temperature over the course of a few hours (NMR data for all complexes are given in the Supporting Information). All heterobimetallic complexes feature a methyl resonance at δ_C_ ≈ 50–60 ppm, typical of a terminal Me group in cationic Salan complexes of group IV metals (Me_D_, Scheme 2 and Figure 1).^78^

**Figure 1:**
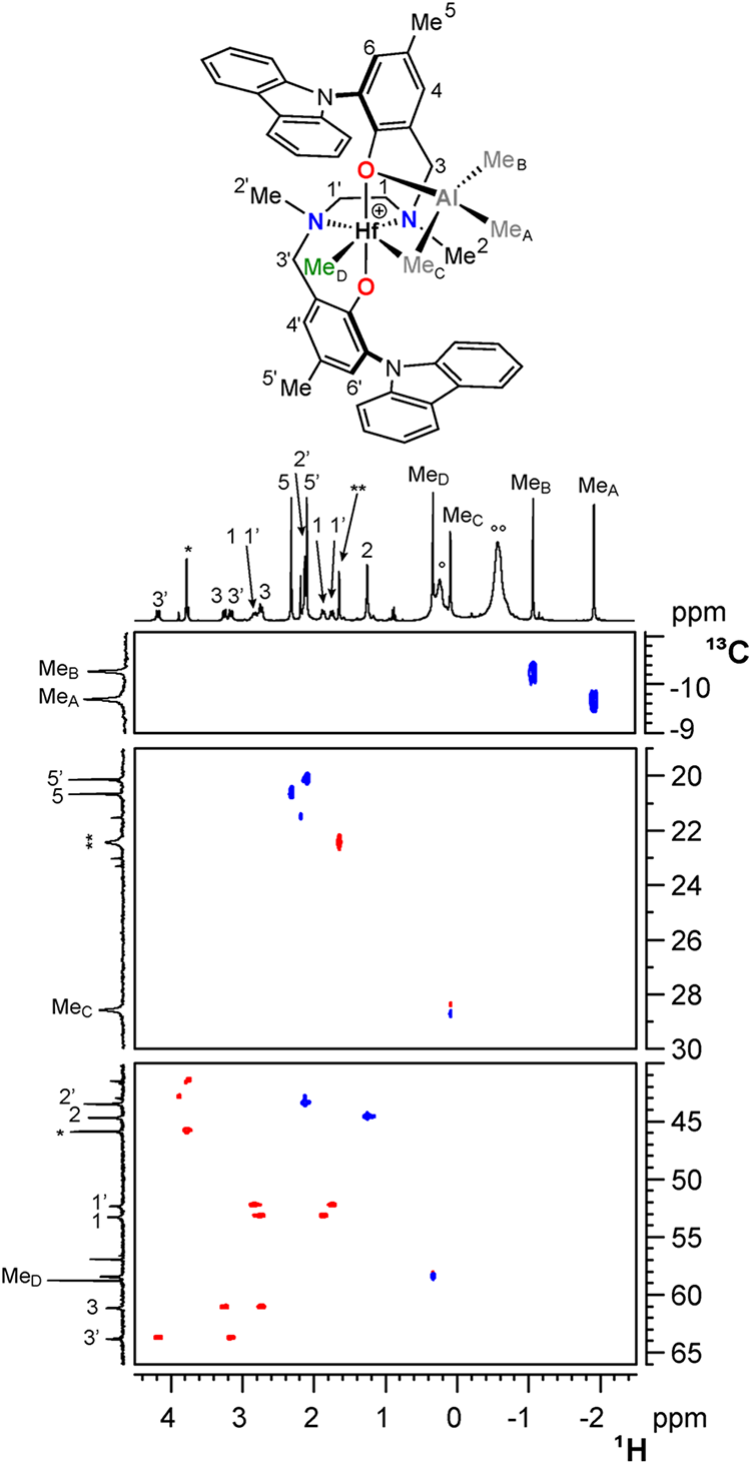
Three sections of the ^1^H,^13^C HSQC NMR spectrum of **3Hf**_**d**_ (C_6_D_5_Cl, 233 K). Asterisk (*) and double asterisks (**) denote resonances of Ph_3_C*CH*_2_Ph and Me_2_Al*CH*_2_Ph, respectively. Open circle (°) and double open circle (°°) denote resonances of bridging and terminal Me moieties of excess AlMe_3_ dimer, respectively.

The ^13^C NMR chemical shifts of NMe moieties are both higher than 40 ppm, establishing that all complexes possesses *C*_1_-symmetry with the [ONNO] ligand coordinated at the metal center in the *fac*-*fac* modality, independent of the geometry of the starting complex **2**.^80^ Three additional singlets in the ^1^H NMR spectra, integrating three protons each, indicate the presence of a single AlMe_3_ molecule in the metal coordination sphere (Scheme 2). One of them shows the corresponding ^13^C NMR chemical shifts in the range δ_C_ ≈ 19–29 ppm, consistent with a bridging methyl group Me_C_,^46^ whereas the other two show δ_C_ values below −3 ppm, typical of terminal methyl groups on aluminum (Me_A_ and Me_B_).^46^

These observations are inconsistent with a symmetric heterobimetallic structural motif featuring two bridging and two terminal Al-bound methyl groups, resulting in an overall *C*_2_-symmetry (**A**, Chart 2) where the pair of bridging Hf-*Me*-Al and the pair of terminal Al-*Me* moieties would be magnetically equivalent to give two separate singlets.^97^ Two alternative structures, **B** and **C** in Chart 2, can be proposed. In **B**, the AlMe_3_ molecule is coordinated *end-on* to the metal center with a single methyl bridge, whereas the aluminum atom is engaged in a further interaction with one of the oxygen atoms of the [ONNO] ligand in structure **C**.^98^ Two additional pieces of evidence consistently demonstrate that the prevalent structure in solution must be **C** in all cases. First, the Me_A_/Me_C_ and Me_A_/Me_D_ NOE contacts (Figure 2) are of comparable intensity; second, Me_B_ shows selective NOE interactions with H3 but not with Me^2^ (Figure 2).

**Figure 2:**
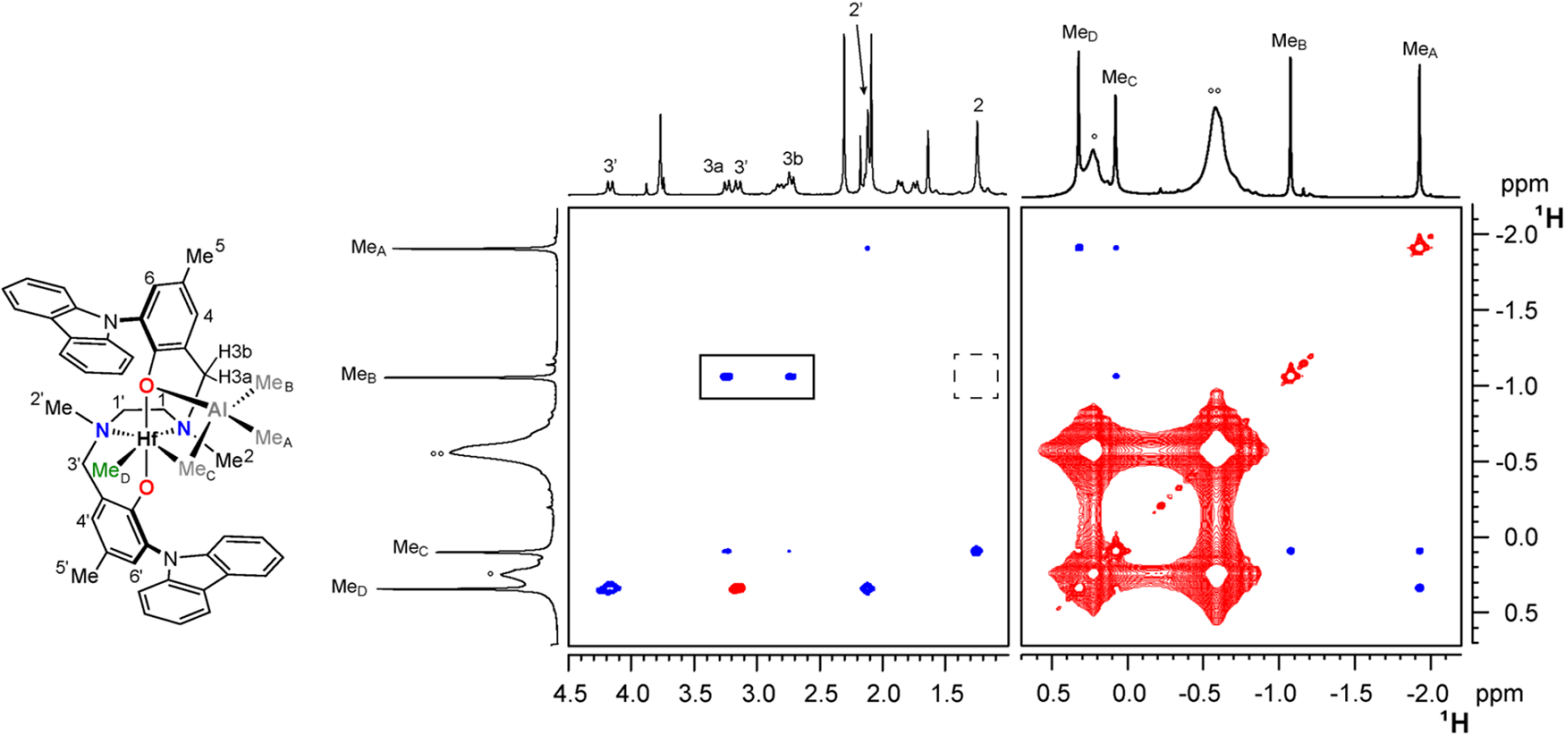
Two sections of the ^1^H ROESY NMR spectrum of **3Hf**_**d**_ (C_6_D_5_Cl, 233 K). Open circle (°) and double open circle (°°) denote resonances of bridging and terminal Me moieties of excess AlMe_3_ dimer, respectively. The rectangle with the solid outline highlights the specific Me_B_/H3a and Me_B_/H3a NOE interactions, whereas the rectangle with the dotted outline highlights the lack of the Me_B_/Me^2^ NOE contact.

**Chart 2:**
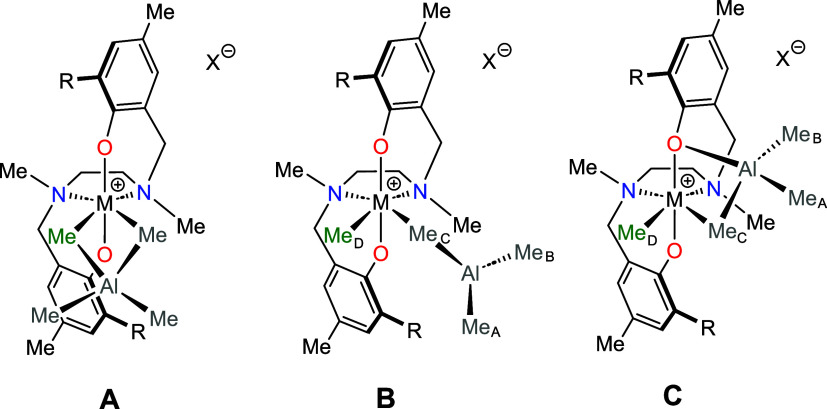
Possible Structures of Heterobimetallic Adducts **3** (X = B­(C_6_F_5_)_4_)

The three possible structural motifs shown in Chart 2 were optimized by DFT calculations for **3Hf**_**a**_ and **3Hf**_**d**_ as the representative cations with alkyl or aromatic R substituents. Structure **C** was found to have the lowest electronic energy in both cases. For **3Hf**_**a**_ (R = *t*Bu), structures **A** and **B** lie 2.3 and 11.2 kcal mol^–1^ above structure **C**, respectively. For **3Hf**_**d**_ (R = carbazolyl), structure **B** (4.6 kcal mol^–1^) is lower in energy than **A** (4.8 kcal mol^–1^). Inspection of optimized structures (Figure 3) strongly supports the NOE data described above: for instance, in structure **C** of **3Hf**_**a**_, the Me_A_/Me_C_ and Me_A_/Me_D_ C–C distances are similar (3.235 and 3.856 Å, respectively), whereas in structure **B**, the latter (5.653 Å) is much longer than the former (3.365 Å).

**Figure 3:**
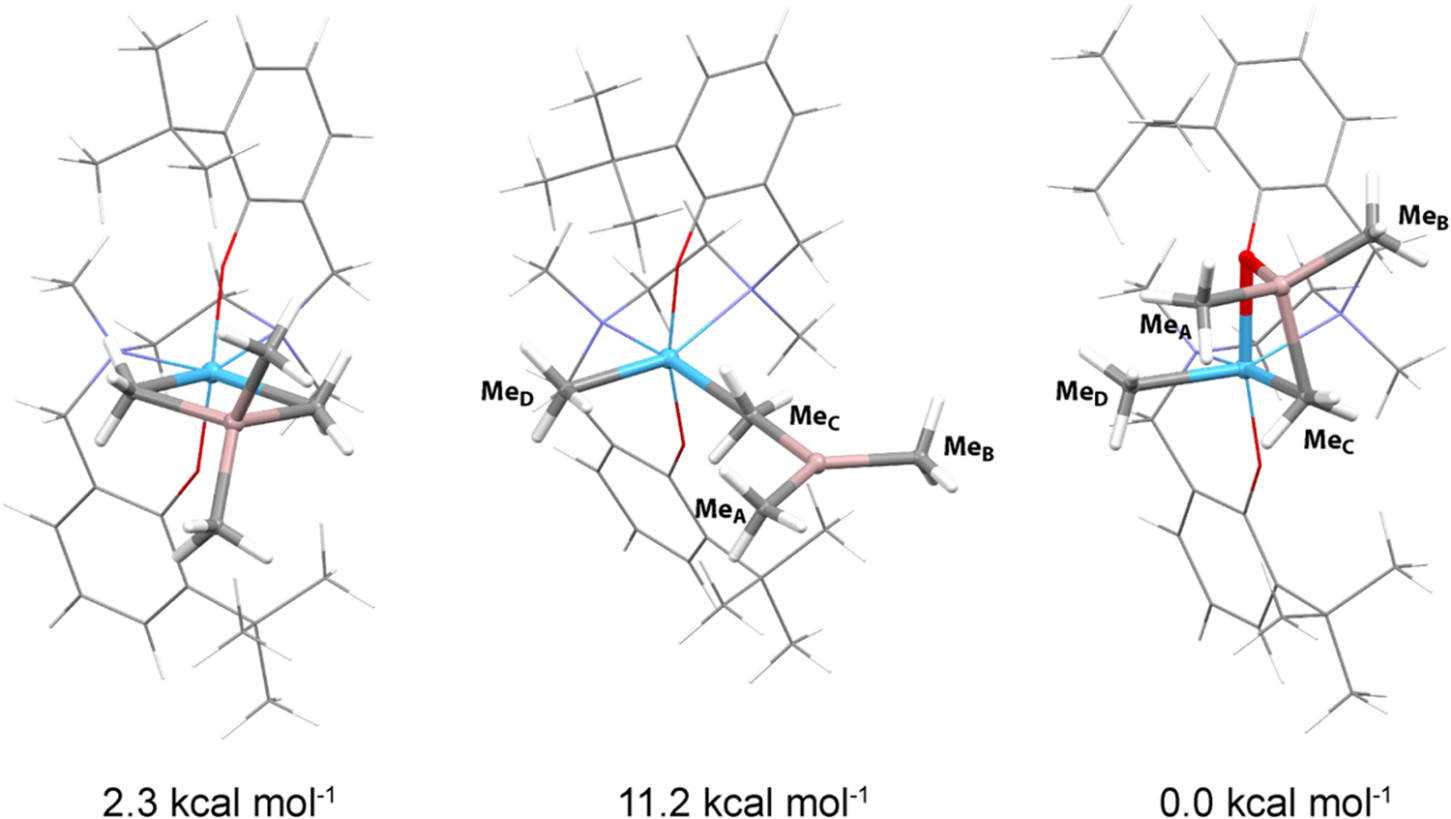
DFT-optimized structures **A** (left), **B** (center), and **C** (right) with the corresponding Gibbs free energies for complex **3Hf**_**a**_.

### Dynamics of Complexes **3** in Solution

The dynamic behavior of complexes **3** was investigated by ^1^H EXSY NMR spectroscopy. One pattern of chemical exchange (Figure 4) appears to be common to all complexes, namely, Me_A_/Me_B_, Me_A_/Me_C_, and Me_B_/Me_C_ exchanges, although, in some cases, the latter could not be readily observed/quantified due to spectral overlapping. Trends of Me_A_/Me_B_ and Me_A_/Me_C_ exchange rates at different temperatures (Supporting Information) are rather similar for all complexes, albeit exchange rates of **3Hf**_**a**_ and **3Hf**_**b**_, bearing aliphatic R substituents, seem slightly faster than those of **3Hf**_**c**_ and **3Hf**_**d**_ bearing aromatic substituents. The corresponding activation parameters, obtained from Eyring plots, are reported in Table 1.

**Figure 4:**
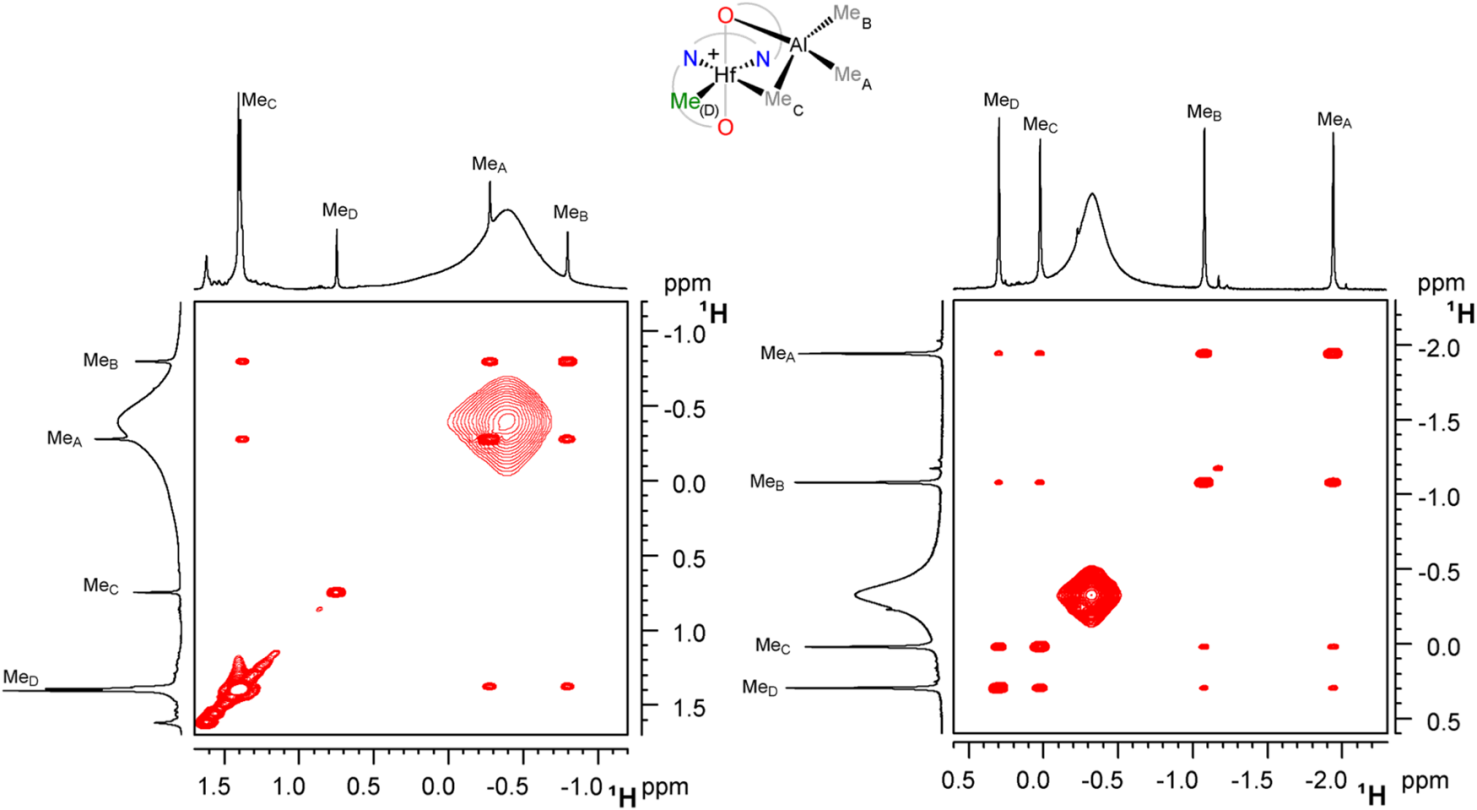
Sections of the ^1^H EXSY NMR spectra of **3Hf**_**a**_ (left) and **3Hf**_**d**_ (right) in C_6_D_5_Cl at 253 K. The broad resonance is due to excess AlMe_3_.

**Table 1:** Activation Parameters (Δ*H* ^‡^, kcal mol^–1^; Δ*S* ^‡^, cal mol^–1^ K^–1^; Δ*G*_298_^‡^, kcal mol^–1^) for Methyl Group Exchanges in Complexes **3Hf**_**a**_-**3Hf**_**d**_ and **3Zr**_**c**_

	Me_A_/Me_B_			Me_A_/Me_C_		
	Δ*H* ^‡^	Δ*S* ^‡^	Δ*G*_298_^‡^	Δ*H* ^‡^	Δ*S* ^‡^	Δ*G*_298_^‡^
**3Hf_a_**	15.7 ± 1.5	4 ± 6	14.4 ± 2.4	17.1 ± 0.9	9 ± 4	14.4 ± 1.4
**3Hf_b_**	14.5 ± 0.8	0 ± 3	14.4 ± 1.2	15.1 ± 1.0	2 ± 4	14.5 ± 1.5
**3Hf_c_**	16.0 ± 0.5	–1 ± 2	16.4 ± 0.8	16.4 ± 0.8	0 ± 3	16.4 ± 1.2
**3Hf_d_**	15.1 ± 0.5	1 ± 1	15.0 ± 1.0	15.0 ± 2.8	–3 ± 11	15.9 ± 4.3
**3Zr_c_**	14.8 ± 0.7	2 ± 3	14.3 ± 1.0			

Activation enthalpies are in the range 14.5–17.1 kcal mol^–1^, whereas activation entropies are small in all cases. It can be reasonably assumed that exchanges among Me_A_, Me_B_, and Me_C_ occur via an intramolecular mechanism, consistent with the lack of observable chemical exchange between methyl groups of coordinated AlMe_3_ and those of free AlMe_3_ in solution, at least within the explored temperature window. Most likely, the exchange process involves the breaking of the M-Me_C_ bond, AlMe_3_ rotation about the Al–O bond, and recoordination to the metal center via a different bridging methyl moiety (Scheme 3, top). This simple pathway also appears to be consistent with the absence of observable chemical exchanges involving the terminal methyl on the metal center (Me_D_) in almost all cases.

**Scheme 3:**
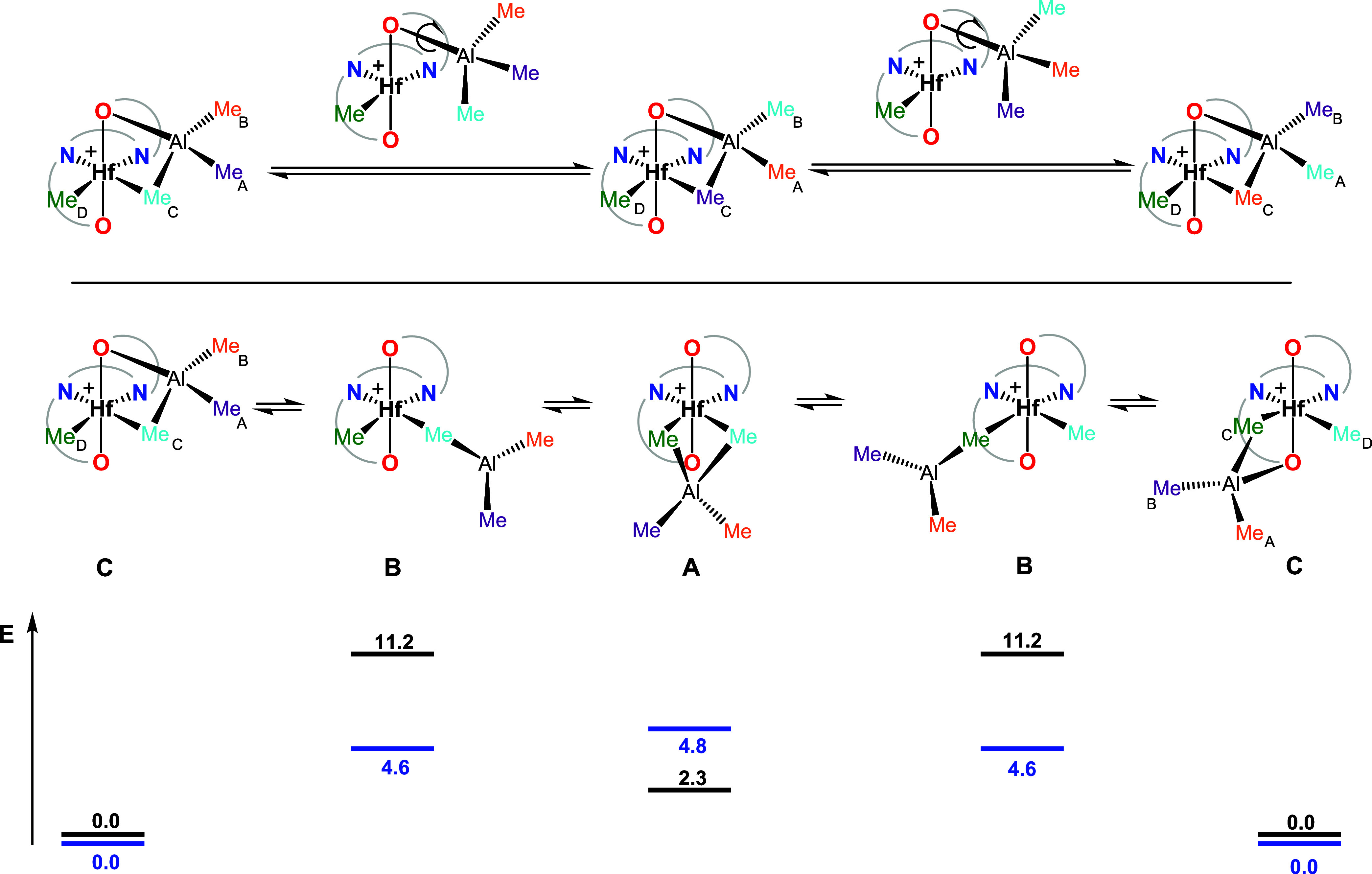
Proposed Mechanism to Explain Me_A_/Me_B_, Me_A_/Me_C_, and Me_B_/Me_C_ Exchanges Observed in Complexes **3** (Top) and the Additional Me_C_/Me_D_ Exchange Observed in Complex **3Hf**_**d**_ (Middle)^a^

a Computed relative energies of structures **A**, **B**, and **C** (kcal mol^–1^) for **3Hf**_**a**_ (black) and **3Hf**_**d**_ (blue) at the TPSSh­(PCM, D_zero_)/TZ//TPSSh/DZ (298 K) level of theory (bottom).

The only exception is **3Hf**_**d**_, where exchanges involving Me_D_ are also observed (Figure 4). The activation parameters of these exchange processes (for example, Δ*H*_MeC/MeD_^‡^ = 15.6 ± 0.8 kcal mol^–1^, Δ*S*_MeC/MeD_^‡^ = 1 ± 3 cal K^–1^ mol^–1^, Δ*G*_MeC/MeD_^‡^ = 15.2 ± 1.2 kcal mol^–1^) are similar to the previous ones, and similar to site epimerization in **3Hf**_**d**_ (Δ*G*_SE_^‡^ = 15.4 ± 3.5 kcal mol^–1^) as quantified by monitoring the Me^2^/Me^2′^ exchange.

It can be proposed that Me_C_/Me_D_ exchange occurs together with concomitant site epimerization at the metal center (Scheme 3, middle). Breaking of the Al–O bond leads to intermediate structure **B** in which Me_C_/Me_D_ permutation may take place via symmetric heterobimetallic intermediate species **A**. Breaking of the Al-Me_C_ bond in **A** and reformation of the Al–O interaction involving the other O-functionality will result in Me_C_/Me_D_ exchange. Relative energy values computed by DFT calculations (Scheme 3, bottom) give additional support to this hypothesis (see above): indeed, for **3Hf**_**d**_, structures **A** and **B** are only 4.8 and 4.6 kcal mol^–1^ above structure **C**, respectively, whereas, for **3Hf**_**b**_ where Me_C_/Me_D_ exchange is not observed, structure **B** is much higher in energy (11.7 kcal mol^–1^) with respect to structure **C**. For **3Hf**_**d**_, we also investigated the exchange processes computationally. For Me_C_/Me_D_ exchange, despite several attempts, we could not locate a transition state connecting **C** and **B**, and the process appears to be simply uphill, likely with a minimal barrier from **B**. Furthermore, no direct pathway from **A** to **C** could be detected. The end-on-coordinated **B** and the bridged structure **A** are connected by a barrier of Δ*G* ^‡^ = 14.1 kcal mol^–1^. Rate limiting for the exchange of the Me_C_/Me_D_ permutation is therefore the formation of the bridged intermediate **A**. For the Me_A_/Me_B_, Me_A_/Me_C_, and Me_B_/Me_C_ exchanges, a rotational TS was identified that was 13.5 kcal/mol higher than that of structure **C**. Both barriers are in good agreement with the experimentally observed barriers around 15 kcal mol^–1^ for both processes in catalyst **3Hf**_**d**_.

### Reaction of Complex **2** with ZnMe_2_

Reaction with ZnMe_2_ was investigated for complexes **2Hf**_**b**_ and **2Hf**_**d**_, as representative examples of systems bearing aliphatic and aromatic R substituents, displaying initial *mer-mer* or *fac-fac* geometries, respectively. In the presence of an excess of ZnMe_2_, the reactions proceed smoothly at room temperature, affording, over the course of a few minutes, the corresponding complexes **4Hf**_**b**_ and **4Hf**_**d**_ as shown in Scheme 4. Full NMR data for **4Hf**_**b**_ and **4Hf**_**d**_ are given in the Supporting Information.

**Scheme 4:**
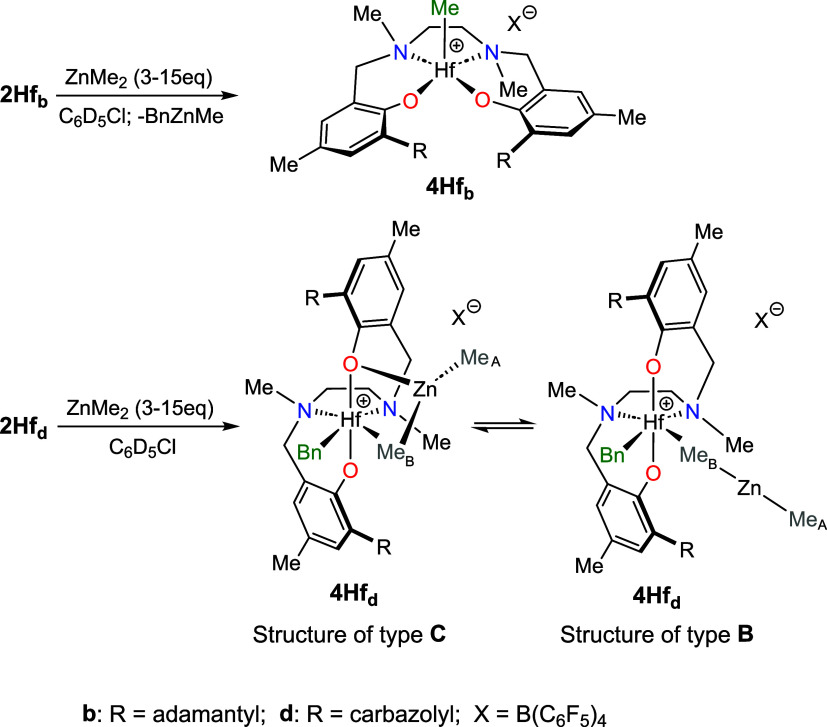
Reaction of Complexes **2Hf**_**b**_ and **2Hf**_**d**_ with ZnMe_2_ Affording Complexes **4Hf**_**b**_ and **4Hf**_**d**_, Respectively

Complex **4Hf**_**b**_ arises from ZnMe_2_/HfBn trans-alkylation as indicated by the absence of the original HfC*H*_2_Ph ^13^C NMR resonance at δ_C_ = 57.7 ppm and the presence of the typical resonance of a terminal Hf*Me* (δ_H_ = 0.49 ppm; δ_C_ ≈ 51.4 ppm).^78^ In contrast with **3Hf**_**b**_, the ^13^C NMR chemical shift of one N*Me* moiety is lower than 40 ppm,^80^ establishing that **4Hf**_**b**_ retains the *mer-mer* geometry of the starting complex **2Hf**_**b**_.

ZnMe_2_/HfBn trans-alkylation does not occur for **2Hf**_**d**_: the ^13^C NMR spectrum of **4Hf**_**d**_ showed the HfC*H*_2_Ph ^13^C NMR resonance at δ_C_ = 68.7 ppm, not much dissimilar to that of starting **2Hf**_**d**_ (δ_C_ = 71.5 ppm). The low ^2^ *J*_HH_ value (8.2 Hz) of benzylic protons and the low value of the C-*ipso* chemical shift (δ_C_ = 129.3 ppm) support the η^2^-coordination of the benzyl moiety. Nevertheless, differently from **2Hf**_**d**_, the aromatic resonances of the benzylic fragment are very broad even at 218 K. The ^13^C NMR chemical shifts of NMe moieties are both higher than 40 ppm, confirming that **4Hf**_**d**_ retains the *fac-fac* geometry of the starting **2Hf**_**d**_. Two additional singlets, integrating for three protons each, are observed at δ_H_ = −0.17 and −2.78 ppm and show scalar correlation with carbon resonances at δ_C_ = −3.1 and −15.0 ppm, respectively. The methyl resonance at δ_H_ = −2.78 ppm (Me_B_) shows strong NOE interactions with both H3 and Me^2^ resonances, a smaller one with the broad aromatic resonance of the benzyl, but no dipolar contacts with the benzylic protons. Consequently, the ZnMe_2_ fragment must be positioned cis with respect to the benzyl substituent, formally in correspondence of the vacant coordination site (i.e., the C_6_D_5_Cl coordinated to the metal in **2Hf**_**d**_ appears to have been displaced). The other methyl group of ZnMe_2_ (Me_A_) shows NOE interaction with aromatic resonances of the carbazolyl substituent(s), but not with the benzylic protons, indicating a specific orientation of the η^2^-coordinated benzyl moiety, in which the aromatic ring is pointing toward the coordinated ZnMe_2_. Strong NOE contacts between the benzylic protons and both H3′ and Me^2′^ give further support. All these data strongly suggest that Me_B_ is oriented toward the metal, but the exact modality of the Hf-ZnMe_2_ interaction cannot be unambiguously determined from the NMR data. Two structures, perhaps in fast equilibrium, appear most likely. The first one resembles a structure of type **C** (Chart 2), in which the Zn atom of the coordinated ZnMe_2_ is engaged in an additional interaction with the phenolate oxygen; the second one is analogous to structures of type **B**, i.e., the ZnMe_2_ moiety is coordinated *end-on* to the Hf center. The preference of structures of types **C** and **B** for **4Hf**_**d**_, with respect to the classical heterobimetallic structure of type **A**, is further corroborated by DFT calculations. Computationally, structure **A** is 9.0 and 2.8 kcal mol^–1^ higher in energy relative to **B** and **C**, respectively.

### Dynamics of Complexes **4Hf_b_** and **4Hf_d_** in Solution

Two dynamic processes occur in **4Hf**_**b**_: site epimerization and methyl exchange between Hf-Me and free ZnMe_2_. The former process is much slower: for instance, at 243 K, *k*_SE_ = 0.05 s^–1^, a value very similar to that of the starting benzyl derivative **2Hf**_**b**_ (0.07 s^–1^). At the same temperature, the magnetization transfer rate constant of the methyl exchange from Hf-Me to ZnMe_2_ is around 45 s^–1^ at [ZnMe_2_] = 0.054 M, corresponding to a second-order rate constant of 830 M^–1^ s^–1^. In reasonable agreement, the second-order rate constant measured for the reverse magnetization transfer of a methyl from ZnMe_2_ to hafnium is 595 M^–1^ s^–1^.

Four distinct exchange processes are observed in the ^1^H EXSY NMR spectra of complex **4Hf**_**d**_, namely, site epimerization, Me_A_/Me_B_ permutation, and exchange of both Me_A_ and Me_B_ with free ZnMe_2_ (Figure 5). Site epimerization is the slowest process (Δ*H*_SE_^‡^ = 22.3 kcal mol^–1^, Δ*S*_SE_^‡^ = 27 cal K^–1^ mol^–1^, Δ*G*_SE_^‡^ = 14.3 kcal mol^–1^) and is also slower than that observed for **2Hf**_**d**_, (Δ*H*_SE_^‡^ = 21.2 kcal mol^–1^, Δ*S*_SE_^‡^ = 31 cal K^–1^ mol^–1^, and Δ*G*_SE_^‡^ = 11.9 kcal mol^–1^), in agreement with a relatively strong interaction between the metal complex and ZnMe_2_.

**Figure 5:**
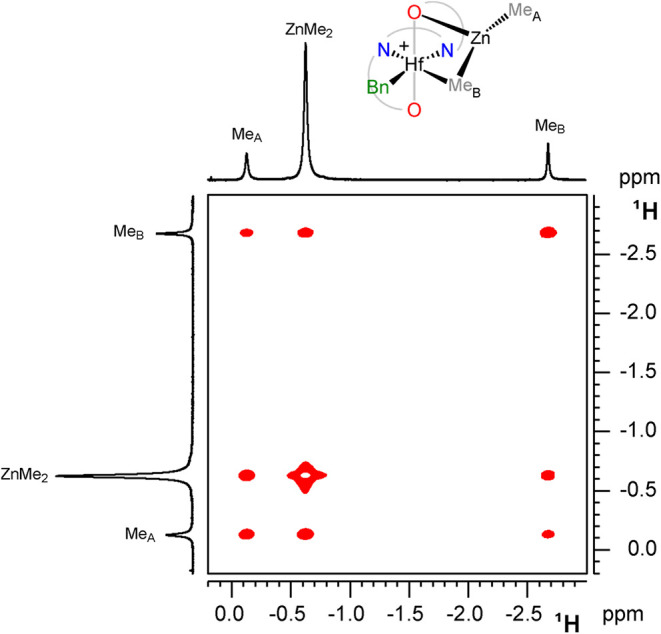
Section of the ^1^H EXSY NMR spectrum of **4Hf**_**d**_ (C_6_D_5_Cl, 233 K).

The measured forward magnetization transfer rate constants (*k*_1obs_) for the other three processes (Me_A_ → Me_B_, Me_B_ → ZnMe_2_, and Me_A_ → ZnMe_2_) display different temperature dependence below 248 K (Figure 6, bottom). An exploration of the dependence of *k*_1obs_ values on [ZnMe_2_], carried out at 233 K, shows that *k*_1obs_ values of Me_A_ → Me_B_ and Me_B_ → ZnMe_2_ exchanges are almost independent of [ZnMe_2_], whereas the *k*_1obs_ value of the Me_A_ → ZnMe_2_ exchange increases linearly as the concentration of ZnMe_2_ increases (Figure 6, top).

**Figure 6:**
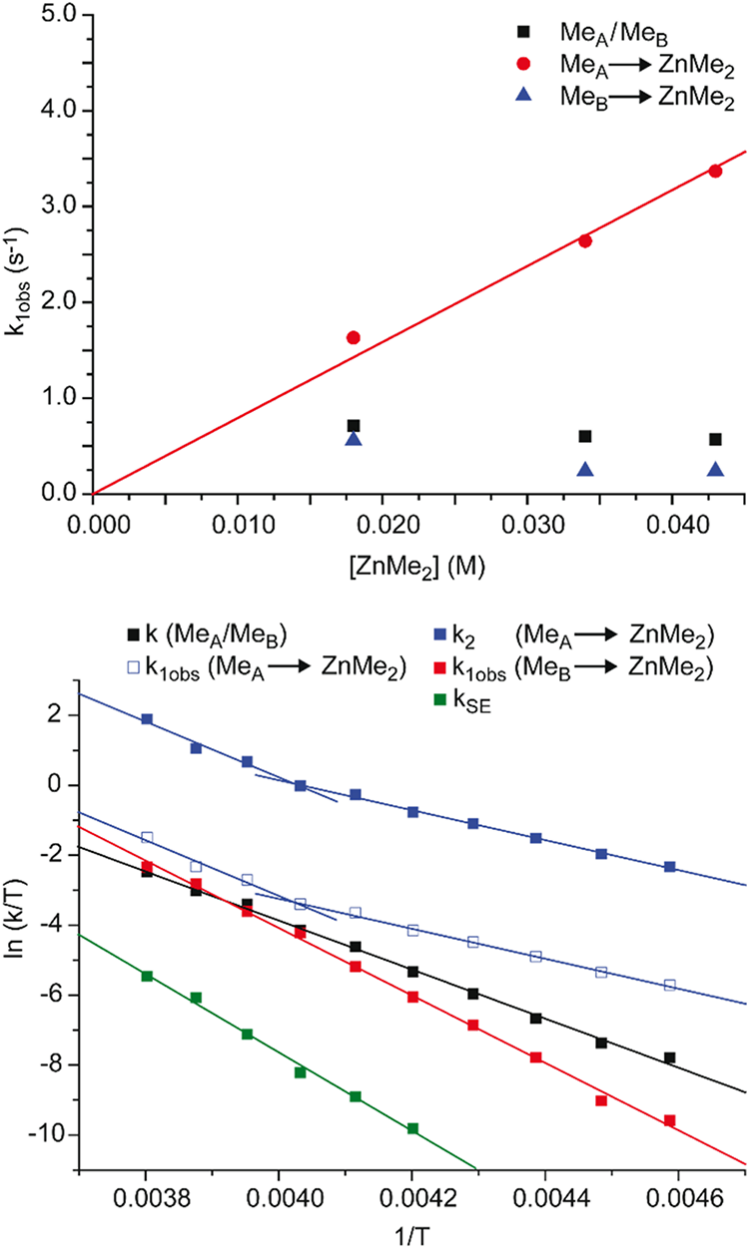
Top: *k*_1obs_ at 233 K as a function of [ZnMe_2_] for Me_A_ → Me_B_, Me_B_ → ZnMe_2_, and Me_A_ → ZnMe_2_. Bottom: Eyring plots of rate constants for the different exchange processes operative in the mixture of **4Hf**_**d**_ plus ZnMe_2_.

In analogy with **3Hf**_**d**_, we conclude that the Me_A_/Me_B_ exchange is prevalently occurring as a first-order process on Hf; indeed, the corresponding activation parameters (Δ*H* ^‡^ = 14.3 kcal mol^–1^, Δ*S* ^‡^ = 3 cal K^–1^ mol^–1^) are similar to those observed for **3Hf**_**d**_ and point to a similar mechanism, in which rotation of the O-bound ZnMe_2_ around the Zn–O bond allows Me_A_/Me_B_ permutation (Scheme 5, pathway A). The independence of *k*_1obs_ on [ZnMe_2_] for the Me_B_ → ZnMe_2_ exchange suggests a first-order dissociative interchange mechanism, possibly solvent-assisted (Scheme 5, pathway B); in agreement, the corresponding Eyring analysis gives a relatively high activation enthalpy (Δ*H*_Me(B)→ZnMe2_^‡^ = 19.2 kcal mol^–1^) and a positive activation entropy (Δ*S*_Me(B)→ZnMe2_^‡^ = 21.3 cal K^–1^ mol^–1^). Pathway B obviously also contributes to the Me_A_/ZnMe_2_ exchange; however, below 248 K, the Me_A_ → ZnMe_2_ exchange is dominated by another associative mechanism (Scheme 5, pathway C) characterized by a small activation enthalpy (Δ*H*_Me(A)→ZnMe2_^‡^ = 8.5 kcal mol^–1^) and a slightly negative activation entropy (Δ*S*_Me(A)→ZnMe2_^‡^ = −12.9 cal K^–1^ mol^–1^). As the temperature increases, both the dissociative and associative pathways (B and C) contribute to the Me_A_ → ZnMe_2_ exchange: indeed, at 263 K, the experimentally measured *k*_1obs_ (59.3 s^–1^ at 263 K) is almost the sum of *k*_1obs_ for Me_B_ → ZnMe_2_ (25.4 s^–1^) and the *k*_1obs_ value extrapolated for the associative pathway of the Me_A_ → ZnMe_2_ exchange (24.3 s^–1^).

**Scheme 5:**
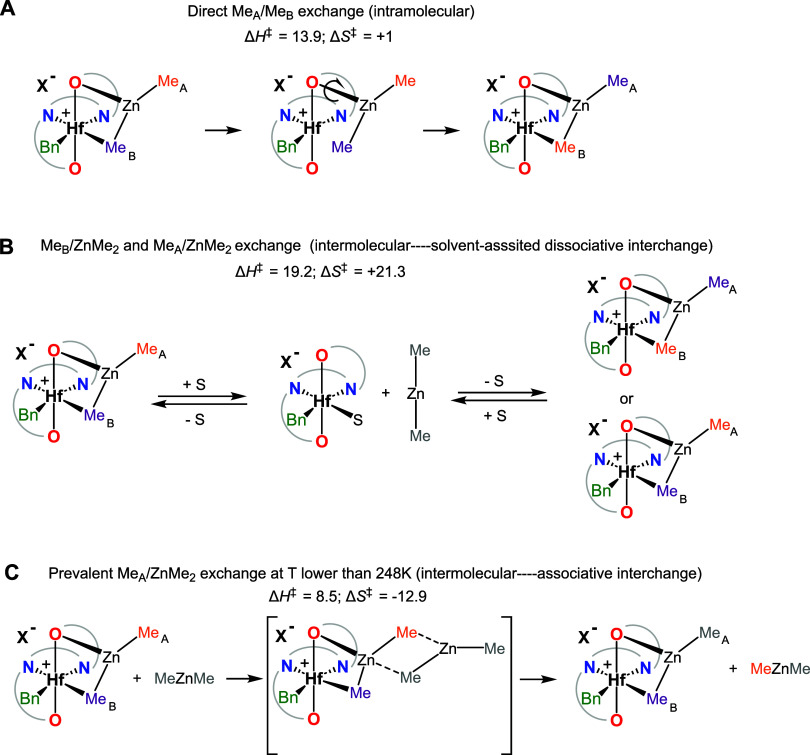
Proposed Mechanism for the Intramolecular (A) and Intermolecular (B, C) Chemical Exchange between Bridging, Terminal, and ZnMe_2_ Methyl Moieties (Δ*H* ^‡^ and Δ*S* ^‡^ Values Are Given in kcal mol^–1^ and cal K^–1^ mol^–1^, Respectively)

### Comparison with Other Heterobimetallic Systems

The results illustrated above indicate that the structure and dynamics of bimetallic adducts between Salan-type cations and AlMe_3_ or ZnMe_2_ are strongly modulated by the nature of ligand substituents while showing similarities and differences with respect to those observed for other post-metallocene and metallocene derivatives. For instance, type **A** (Chart 2) is the only structure detected in solution for [Cp_2_Hf-μMe_2_-AlMe_2_]^+^ and related cyclometalated species;^46,53,54^ in such systems, no chemical exchange is observed between bridging and terminal methyl moieties, but fast exchange occurs between terminal methyl groups and external AlMe_3_. In contrast, the type **C** structure is preferred for **3Hf**_**a**_–**3Hf**_**d**_ methyl cations, and chemical exchange, modulated by the nature of phenolate ring substituents, involves both bridging and terminal methyls, but it is not detectable between the methyls of the cations and those of free AlMe_3_. Interestingly, this behavior closely resembles that of Hf–Al heterobimetallic systems based on pyridylamido Hf complexes,^51^ a system known to efficiently promote CSP and shown to form unusual bimetallic adducts. Although heteroatoms are not involved in this case, a nontraditional heterobimetallic adduct involving a ligand, the carbon atom of the Hf-*C*_aryl_ bond, is formed.

For Hf–Zn adducts involving metallocenes, the type **A** structure could only be observed for cyclometalated species below 233 K^53^ as decomposition readily occurs at higher temperatures, but no data concerning chemical exchange between methyl groups of the cation and ZnMe_2_ were reported. On the contrary, type **B** and **C** structures appear to dominate the speciation of **4Hf**_**d**_ in solution, where the selectivity and temperature dependence of chemical exchanges clearly indicate the onset of different exchange mechanisms capable of interconverting methyl moieties of the cations with those of free ZnMe_2_. Although no quantitative data were reported in the original paper by Mountford and Clot,^70^ they also described a complex methyl group exchange pattern for heterobimetallics formed by Ti-imido methyl cations and ZnMe_2_, indicating again that the presence of heteroatom functionalities in the first coordination sphere of the transition metal is indeed playing a pivotal role. Finally, replacing the carbazolyl substituent in **4Hf**_**d**_ with adamantyl resulted in the stabilization of a monomethyl derivative (**4Hf**_**b**_) with no evidence for subsequent heterobimetallic formation: however, in this latter system, very fast exchange occurs between Hf-Me and ZnMe_2_ (*k*_ex_ ≈ 700 M^–1^ s^–1^ at 243 K), even faster than that observed for cationic post-metallocene pyridylamido Hf-ZnMe_2_ adducts (*k*_ex_ ≈ 100 M^–1^ s^–1^ at 243 K).^51^

### Connection of the Present Results to CTTP Reactivity

The CCTP reactivity, i.e., the (reversible) transfer of a growing polymeryl chain to a main-group metal, requires that bimetallic intermediates of the type **I** open up differently from how they form. However, early transition metals often have strong agostic interactions that main-group metals are incapable of forming, and this stabilization energy would be lost in the transfer. Here, we observed in most cases rapid transfer of Bn groups to Al or Zn, but for **4Hf**_**d**_, with a relatively open active pocket formed by the *N*-carbazolyl group, experimental evidence indicates that the benzyl remains η^2^-coordinated, even in a bimetallic complex. Changing the R-group to 1-Ad in **4Hf**_**b**_ enables rapid Bn transfer, possibly because η^2^-coordination is sufficiently destabilized.

## Conclusions

The reactions of cationic Hf­(IV) complexes bearing [ONNO] Salan-type ligands with AlMe_3_ and ZnMe_2_ were studied by means of NMR spectroscopy and DFT computations. The main purpose of this work was to obtain insights into the role played by Lewis-basic functionalities present in the ligand in modulating the structure and dynamics of their heterobimetallic adducts. The results unambiguously demonstrate strict differences with respect to typical metallocene systems. (i) Salan Hf–Al heterobimetallic complexes feature four-membered species in which one μ-alkyl and one oxygen atom of the ligand are bridging the two metals. (ii) Exchange between bridging and terminal Me moieties on Al occurs preferentially *via* an intramolecular mechanism without the involvement of external AlMe_3_. (iii) While similar heterobimetallic structures form when ZnMe_2_ is used, both bridging and terminal Me groups exchange with external ZnMe_2_. Values of activation enthalpy and entropy suggest a dissociative mechanism for the exchange involving the bridging methyl and an associative mechanism for the terminal methyl.

The main outcome of these experiments is that, first, post-metallocenes allow a much larger structural diversity of heterobimetallic interactions with respect to metallocenes, as donor atoms like O, N, or even C^51^ are key actors in assisting the formation of hetero dimers. Second, a subtle balance between the nature of CTA, metal complex, and substituent R determines whether alkyl transfer occurs rapidly or slowly (if at all). Understanding the design principles for a rapid and reversible chain transfer will unlock novel catalysts for CSP and CCTP, and further efforts in this direction are currently underway in our laboratories.

## Experimental Section

### Materials and Methods

All manipulations were carried out under an inert atmosphere using a Schlenk line interfaced to a high vacuum line (<10^–5^ Torr) and a nitrogen-filled MBraun Labstar Glovebox (<0.5 ppm of O_2_ and <0.5 ppm of H_2_O). Chlorobenzene-d_5_ (Apollo Scientific Ltd.) was degassed through multiple freeze–pump–thaw cycles on a high vacuum line, dried over CaH_2_, vacuum transferred to a dry storage tube with a PTFE valve, and stored over molecular sieves (4 Å) previously activated for 24 h at ca. 200–230 °C under dynamic vacuum. AlMe_3_ and ZnMe_2_ (2.0 M in toluene) were purchased from Sigma-Aldrich and used as received. **Caution!** AlMe_3_ and ZnMe_2_ solutions are flammable and should be handled with care in an inert atmosphere and deactivated prior to disposal. The activator [CPh_3_]­[B­(C_6_F_5_)_4_] Apollo Scientific was used as received. Ligands **L**_**a**_ ^99^ and **L**_**c**_ ^73^ as well as dibenzyl complexes **1Zr**_**a**_ **-1Zr**_**d**_, **1Hf**_**b**_, and **1Hf**_**d**_ were prepared following the published procedures.^73,100,101^ The synthetic procedure for the new dibenzyl complexes **1Hf**_**a**_ and **1Hf**_**c**_ is given in the Supporting Information. The MS spectra were recorded using an Agilent Technologies 8890 GC/5977C MSD system. All samples for NMR measurements were prepared inside the glovebox using flame-dried NMR tubes equipped with a PTFE valve (J-Young NMR tubes). The NMR spectra were recorded using a Bruker Avance III 400 spectrometer equipped with a smartprobe or a Bruker Avance NEO 600 spectrometer equipped with Prodigy Bruker Cryoprobe. Residual solvent resonances were used for referencing, and the reported chemical shifts are relative to external TMS (^1^H and ^13^C) and CCl_3_F (^19^F). NMR characterization was carried out by means of multinuclear and multidimensional NMR experiments using standard pulse sequences available in the Bruker library. ^19^F NMR resonances of B­(C_6_F_5_)_4_^–^ are always comparable with those of the “free” anion. For example, at 233 K: δ (ppm) = −131.7 (s, *o*-F), −161.3 (t, ^3^J_FF_ = 20.4 Hz, *p*-F), −165.2 (pseudo t, ^3^J_FF_ = 20.4 Hz, *m*-F).

### General Procedure for the Generation of the Cationic Benzyl Complexes

In the glovebox, the desired amount of the neutral dibenzyl complex of choice (**1Zr**_**a**_-**1Zr**_**d**_; **1Hf**_**a**_-**1Hf**_**d**_) and 0.98 equiv of [CPh_3_]­[B­(C_6_F_5_)_4_] were loaded into a J-Young NMR tube. Then, 0.6 mL of chlorobenzene-d_5_ was added and the mixture was shaken, resulting in a yellow or orange clear solution depending on the complex used. The resulting cationic complexes (**2Zr**_**a**_-**2Zr**_**d**_; **2Hf**_**a**_-**2Hf**_**d**_) were characterized by low-temperature NMR spectroscopy. NMR data are reported in the Supporting Information.

General procedure for the generation of a heterobimetallic adduct. In the glovebox, the desired amount of AlMe_3_ or ZnMe_2_ was injected directly into a precooled solution of cationic complex within a J-Young NMR tube. The mixture was shaken and allowed to reach 298 K within a few minutes. The NMR tube was taken outside the glovebox and transferred to the precooled probe of the NMR spectrometer for analysis. The NMR data of the resulting species (**3Zr**_**a**_-**3Zr**_**c**_; **3Hf**_**a**_-**3Hf**_**d**_; **4Hf**_**b**_; **4Hf**_**d**_) are reported in the Supporting Information.

### Kinetic Studies of Exchange Processes

Variable temperature ^1^H EXSY NMR measurements were acquired using the standard “noesygptp” pulse sequence available in the Bruker pulse program library. The relaxation delay was set to 1 s, and the mixing time value was adjusted between 2.7 and 800 ms, depending on the rate of chemical exchange at each given temperature. Typically, a matrix of 512 × 512 data points was used for acquisition, and the raw data were processed using zero-filling to 2048 data points in both spectral dimensions. The spectral window and the number of transients were optimized depending on the distribution of relevant resonances and the sample concentration. Values of forward and backward magnetization transfer rate constant (*k*_1obs_ and *k*_–1obs_, both in s^–1^) were evaluated by the method proposed by Perrin^102^ and were calculated from the integration of the 2D spectra by using the EXSYCALC software package.^103^ For intramolecular processes, such as Site Epimerization (SE) and exchange between different methyl moieties belonging to heterobimetallic adducts, magnetization transfer rate constant values (*k*_obs_) correspond to *k*_SE_, *k*_Me(A)‑Me(B)_, *k*_Me(A)‑Me(C)_, or *k*_Me(C)‑Me(D)_; the mathematical average of the experimental forward and backward magnetization transfer rate constants are given in the Supporting Information. For intermolecular processes involving exchange of methyl groups between the heterobimetallic adducts and “free” ZnMe_2_, *k*_–1obs_ values obtained from EXSYCALC software were multiplied by 2 to account for the number of equivalent methyl groups in ZnMe_2_. For associative processes, the forward and backward macroscopic kinetic rate constants were computed as *k*_1_ = *k*_1obs_/[ZnMe_2_] and *k*_–1_ = *k*_–1obs_/[Hf]. The concentrations of the species at equilibrium were estimated by quantitative ^1^H NMR spectra using an external standard. The activation parameters of dynamic motions were estimated from the corresponding Eyring plots; errors on activation enthalpy and activation entropy were determined from the quality of linear fitting and computed at a 95% confidence interval. Experimental values are reported in the Supporting Information.

### Computational Details

The geometries of the complexes were fully optimized using the Gaussian 16 software package.^104^ The BOpt software package was employed for data collection.^105^ Following the protocol proposed in ref,^106^ the TPSSh^107^/cc-pVDZ­(-PP)^108–111^ level of theory, using a small core pseudo-potential on the metal,^112,113^ was employed for optimization. This protocol has been successfully used, in combination with TPSSh-D_zero_/cc-pVTZ­(-PP) single-point energy corrections, to address polymerization-related problems in post-metallocene chemistry.^114,115^ The density fitting approximation (Resolution of Identity, RI)^116–119^ and the standard Gaussian 16 quality settings [Scf = Tight and Int­(Grid = UltraFine)] were used at the optimization stage and for single-point energy (SP) calculations. All structures represent true minima (as indicated by the absence of imaginary frequencies). Grimme’s dispersion correction,^120^ with zero damping and the polarizable continuum model (PCM)^121^ for solvent corrections (toluene or chlorobenzene), was employed at the SP stage. Final energies were then combined with thermal corrections (enthalpy and entropy, 298 K, 1 bar) to obtain final free energies; entropy corrections were scaled by 0.67 to account for the reduced freedom of movement in solution.^122–124^ The CREST program^125^ was used to sample the conformational space and generate guess structures for full DFT optimization. NMR predictions were computed at the SP stage with the Gauge-Independent Atomic Orbital (GIAO) method.^126–129^

## Supplementary Material





## Notes

Materials and methods, synthetic and computational details, NMR data of the complexes, and details about kinetic studies (PDF)
Cartesian coordinates for DFT-optimized structures (XYZ)

## #

Department of Chemical and Pharmaceutical Sciences, Via Licio Giorgieri, 1-34127 Trieste, Italy

## ∇

EaStCHEM, School of Chemistry, University of St. Andrews, North Haugh, St. Andrews KY16 9ST, U.K.
