# Supplementary material for: Ligand-Assisted Formation of Heterobimetallic Adducts of AlMe3 and ZnMe2 with Zr and Hf Salan Catalysts for Olefin Polymerization
Source: Inorg Chem. 2025 Nov 27;64(49):23947–60. doi: 10.1021/acs.inorgchem.5c03732 (PMC12709582; doi:10.1021/acs.inorgchem.5c03732)
Supplement: Supplementary file 1 [file ic5c03732_si_001.pdf]

Supporting Information

Ligand-Assisted Formation of Heterobimetallic Adducts of $AlMe_3$ and $ZnMe_2$ with Zr and Hf Salan Catalysts for Olefin Polymerization

Martina Morello,[a] Anna Dall'Anese,[b,e,†] Luca Rocchigiani,[b] Christian Ehm,[c,e,*] Roberta Cipullo,[c,e] Pavel S. Kulyabin,[d,‡] Dmitry V. Uborsky,[d,*] Alexander Z. Voskoboynikov,[d] Alceo Macchioni,[b,e] Vincenzo Busico[c,e] Cristiano Zuccaccia,[b,e,*]

a. Department of Industrial Engeneering, University of Padua, via Gradenigo 6/a, 35131 Padova, Italy.

b. Department of Chemistry, Biology and Biotechnology and CIRCC, University of Perugia, via Elce di Sotto 8, 06123 Perugia, Italy.

c. Department of Chemical Sciences, Federico II University of Naples, via Cinthia, 80126 Napoli, Italy.

d. Department of Chemistry, Lomonosov Moscow State University, 1/3 Leninskie Gory, 119991 Moscow, Russia

e. DPI, P.O. Box 902, 5600 AX Eindhoven, the Netherlands.

† Present address: Department of Chemical and Pharmaceutical Sciences, Via Licio Giorgieri, 1 - 34127 Trieste, Italy.

‡ EaStCHEM, School of Chemistry, University of St. Andrews, North Haugh, St. Andrews KY169ST, U.K.

Email: cristiano.zuccaccia@unipg.it, christian.ehm@unina.it, duborsky@med.chem.msu.ru

Content

Experimental Section S2
  Synthesis of $1Hf_a$ S3
  Synthesis of $1Hf_c$ S5
General procedure for the generation of the cationic benzyl complexes S8
General procedure for the generation of heterobimetallic adducts. S16
Kinetic studies of exchange processes S34
Computational details S46

Experimental section

All manipulations were carried out under inert atmosphere using a Schlenk line interfaced to a high vacuum line ($<10^{-5}$ Torr) and a nitrogen-filled MBraun Labstar Glovebox (<0.5 ppm of O_2 and <0.5 ppm of H_2O). Chlorobenzene-d_5 (Apollo Scientific Ltd.) was degassed through multiple freeze-pump-thaw cycles on a high vacuum line, dried over CaH_2 and vacuum transferred to a dry storage tube with a PTFE valve and stored over molecular sieves (4 Å) previously activated for 24 h at ca. 200−230 °C under dynamic vacuum. $AlMe_3$ and $ZnMe_2$ (2.0 M in toluene) were purchased from Sigma-Aldrich and used as received (**Caution**! $AlMe_3$ and $ZnMe_2$ are extremely pyrophoric under air; therefore, all manipulations must be carried out under an inert atmosphere using standard Schlenk-line or glovebox techniques). The activator $[CPh_3][B(C_6F_5)_4]$ Apollo Scientific was used as received. Ligands L_a[1] and L_c[2] as well as dibenzyl complexes **$1Zr_a$-$1Zr_d$**, **$1Hf_b$** and **$1Hf_d$** were prepared following reported published procedure.[2,3]

MS spectra were recorded using Agilent Technologies 8890 GC/5977C MSD System. All samples for NMR measurements were prepared inside the glove box using flame-dried NMR tubes equipped with a PTFE valve (J-Young NMR tubes). NMR spectra were recorded using a Bruker Avance III 400 spectrometer equipped with a smartprobe or a Bruker Avance NEO 600 spectrometer equipped with the ProdigyTM Bruker Cryoprobe. Residual solvent resonances were used for referencing and the reported chemical shifts are relative to external TMS (^1H and ^{13}C) and CCl_3F (^{19}F). NMR characterization was carried out by means of multinuclear and multidimensional NMR experiments using standard pulse sequences available in the Bruker library. To describe the multiplicity of the signals, the following abbreviations are used: s, singlet; bs, broad singlet; d, doublet; bd, broad doublet; dd, doublet of doublets; t, triplet; and m, multiplet. ^{19}F NMR resonances of $B(C_6F_5)_4^-$ are always compatible with those of "free" anion. For example, at 233 K: δ (ppm) = -131.7 (s, *o*-F), -161.3 (t, $^3J_{FF}$ = 20.4 Hz, *p*-F), -165.2 (pseudo t, $^3J_{FF}$ =20.4 Hz , *m*-F).

Synthesis of $1Hf_a$.

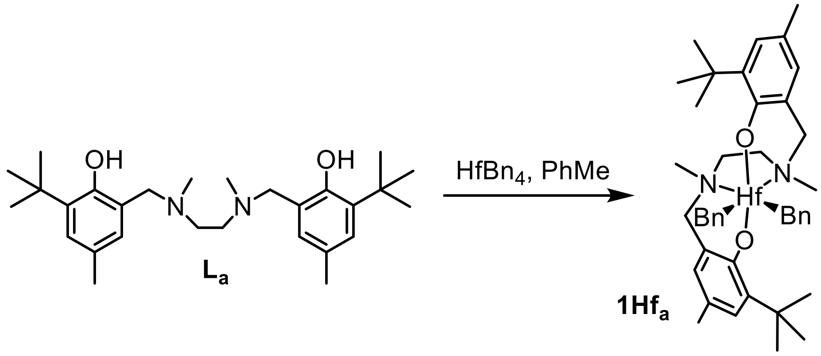

Ligand L_a (1.0 g, 2.3 mmol) was dissolved in warm toluene (150 mL) and a solution of $HfBn_4$ (1.23 g, 2.3 mmol) in toluene (15 mL) was added. The mixture was stirred at r.t. overnight. Then, the solution was concentrated in vacuum and filtered through a glass frit to give the product as a white solid (1.27 g, 77 %). Anal. Calc. for $C_{42}H_{56}HfN_2O_2$: C, 63.10; H, 7.06; N, 3.50. Found: C, 62.93; H, 7.14; N, 3.44.

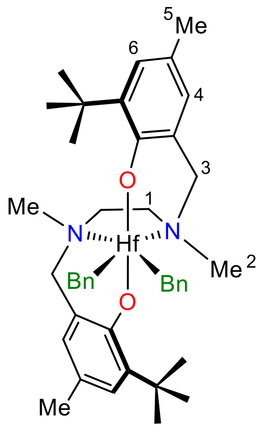

$1Hf_a$: ^{1}H-NMR (C_6D_6, 298K, δ in ppm, J in Hz): δ =7.29 (m, H6), 7.18 (dd, H_{ortho}), 7.09 (m, H_{meta}), 6.73 (tt, H_{para}), 6.4 (d, H4), 3.66 (d, H3), 2.66 (d, H Bn), 2.51 (d, H1), 2.44 (d, H Bn), 2.31 (d, H3), 2.23 (s, H5), 1.73 (s, H2), 1.72 (s, H *t*Bu), 0.79 (d, H1).

^{13}C-NMR (C_6D_6, 298K,, δ in ppm): δ = 156.67 (s, C_{quat}), 149.57 (s, C_{ipso}), 138.04 (s, C_{quat}), 128.56 (s, C4), 128.14 (s, C6), 127.97 (s, C_{meta}), 127.24 (s, C_{quat}), 126.51 (s, H_{ortho}), 124.86 (s, C_{quat}), 120.818 (s, C_{para}), 75.75 (s, C Bn), 63.21 (s, C3), 52.39 (s, C1), 45.37 (s, C2), 30.13 (s, C *t*Bu), 30.07 (s, C *t*Bu), 20.65 (s, C5).

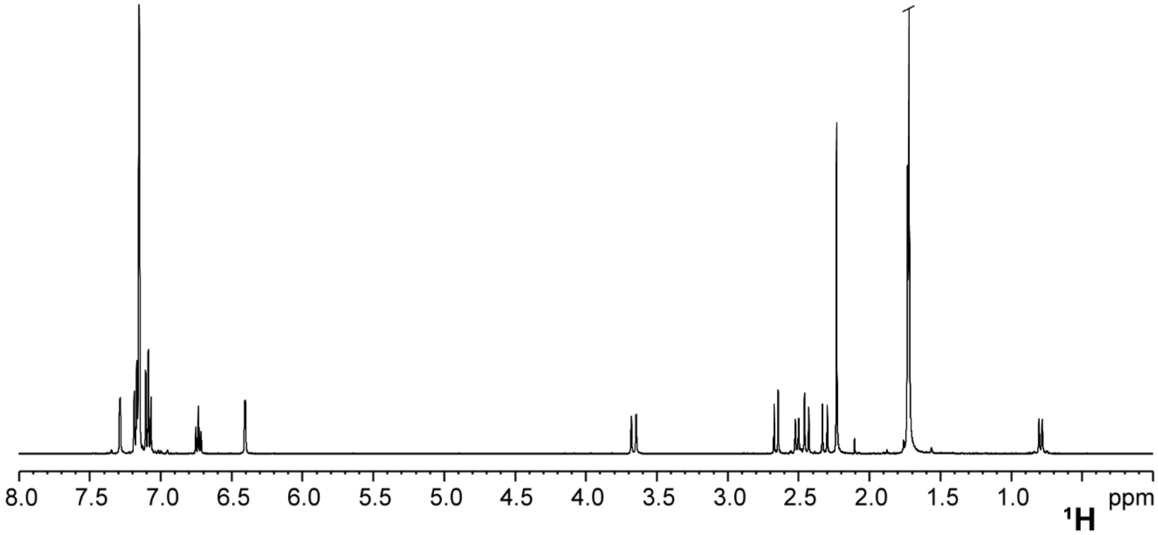

Figure S1. ^{1}H NMR spectrum of **$1Hf_a$** (C_6D_6, 298 K).

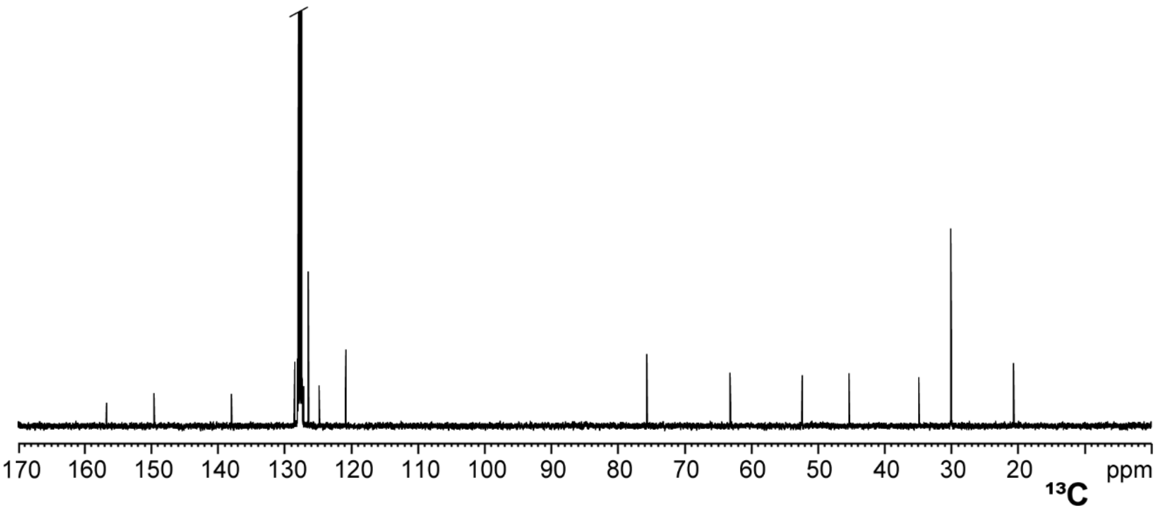

Figure S2. ^{13}C NMR spectrum of **1Hf$_a$** (C_6D_6, 298 K).

Synthesis of $1Hf_c$.

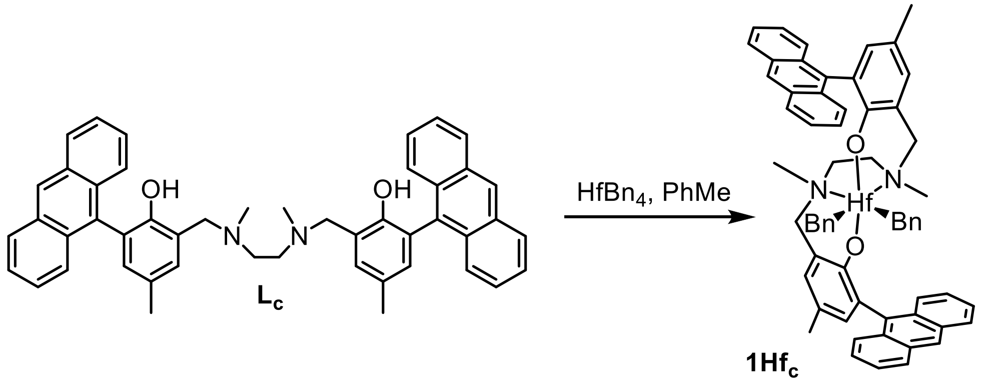

Ligand L_c (451 mg, 0.662 mmol) was dissolved in warm toluene (100 mL) and a solution of $HfBn_4$ (319 mg, 0.662 mmol) in toluene (10 mL) was added. The mixture was stirred at r.t. overnight. Then, the solution was concentrated in vacuum and filtered through a glass frit to give the product as a white solid (347 mg, 50 %). Anal. Calcd. for $C_{62}H_{56}HfN_2O_2$: C, 71.63; H, 5.43; N, 2.69. Found: C, 71.74; H, 5.49; N, 2.66.

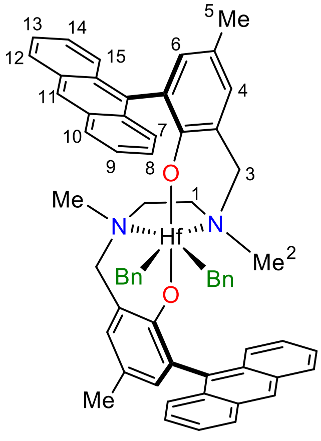	**$1Hf_c$:** ^1H-NMR (C_6D_6, 298K, δ in ppm, J in Hz): δ = 8.33 (s, H11), 8.11 (m, H7), 7.99 (m, H12), 7.87 (m, H10), 7.85 (m, H15), 7.35 (m, H13), 7.31 (m, H8-9), 7.18 (m, H14), 7.08 (m, H6), 6.73 (m, H_{meta}), 6.63 (m, H_{para}), 6.55 (m, H4), 5.79 (m, H_{ortho}), 3.28 (d, H3), 2.57 (d, H1), 2.18 (d, H3), 2.17 (s, H5), 1.35 (s, H2), 0.89 (d, H Bn), 0.87 (d, H1), 0.68 (d, H Bn).<br>^{13}C-NMR (C_6D_6, 298K, δ in ppm): δ = 156.04 (s, C_{quat}), 147.51 (s, C_{ipso}), 134.8 (s, C_{quat}), 133.13 (s, C6), 133.02 (s, C_{quat}), 129.98 (s, C4), 129.06 (s, C12), 128.53 (s, C10), 127.85 (s, C7), 127.65 (C_{meta}), 127.17 (s, C_{ortho}), 126.88 (s, C15), 126.77 (s, C11), 125.54 (s, C8-9), 124.80 (s, C_{quat}), 124.30 (s, C13), 124.12 (s, C14), 120.54 (s, Cp), 70.10 (s, Bn), 62.44 (s, C3), 52.60 (s, C1), 44.64 (s, C2), 20.32 (s, C5).

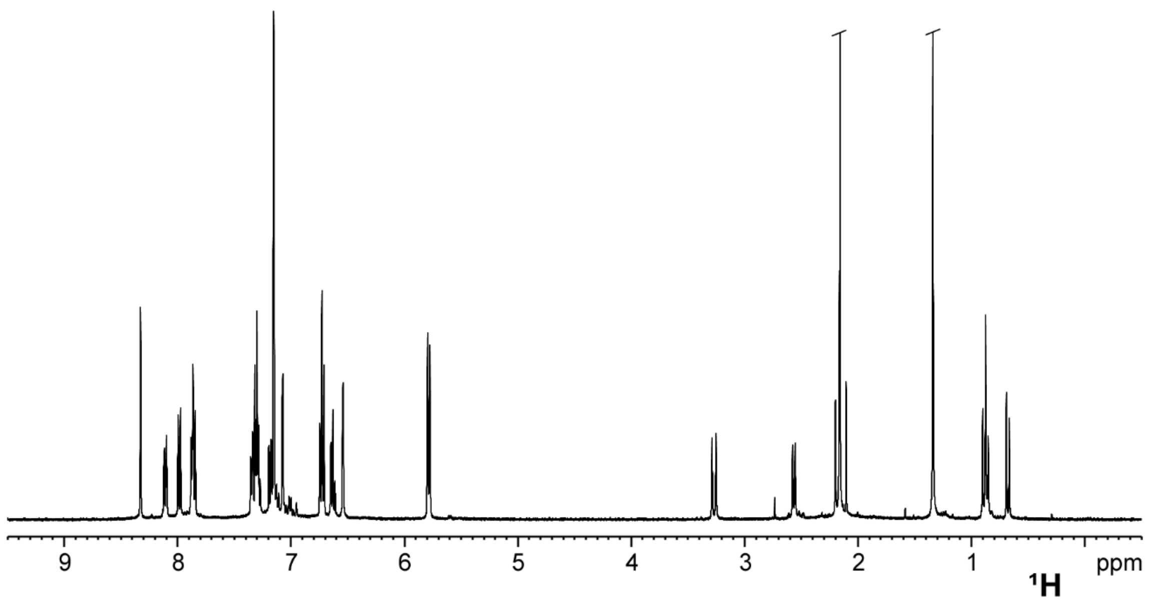

Figure S3. ^{1}H NMR spectrum of **1Hf$_c$** (C_6D_6, 298 K).

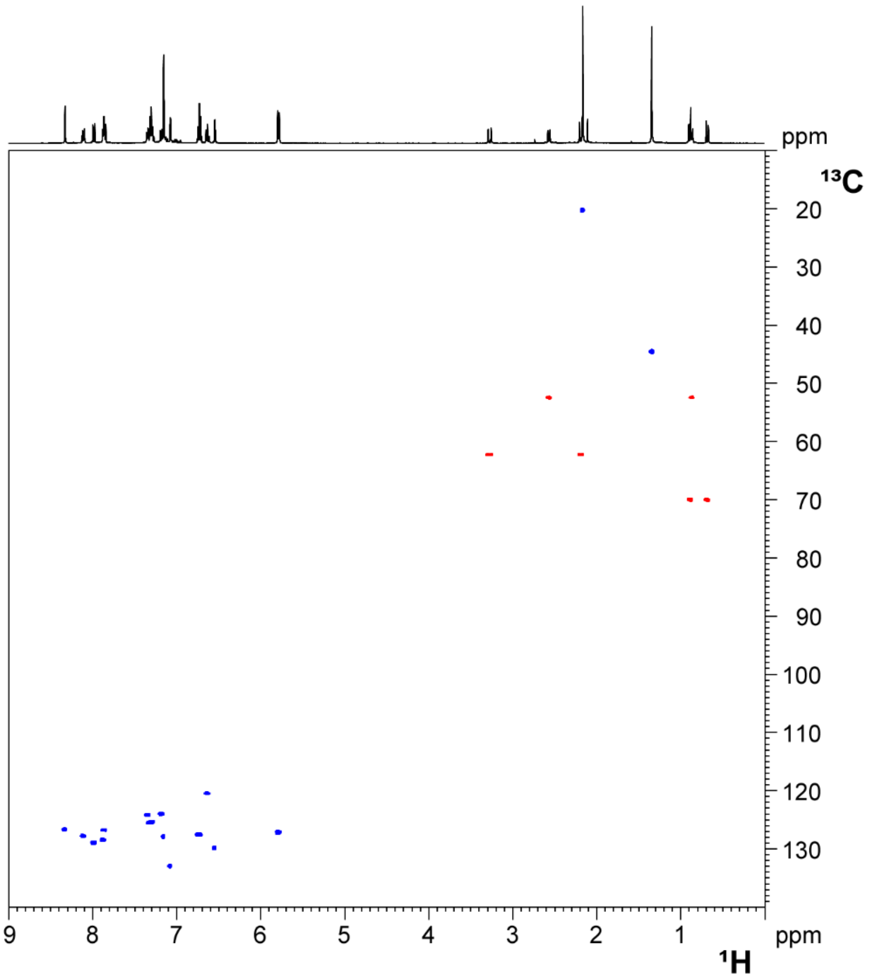

Figure S4. 1H 13C HSQC NMR spectrum of **$1Hf_c$** (C_6D_6, 298 K).

General procedure for the generation of the cationic benzyl complexes.

In the glovebox, the desired amount of the neutral dibenzyl complex of choice (**$1Zr_a$-$1Zr_d$**; **$1Hf_a$-$1Hf_d$**) and 0.98 equiv. of $[CPh_3][B(C_6F_5)_4]$ were loaded into a J-Young NMR tube. Then, 0.6 mL of chlorobenzene-*d*5 were added and the mixture shaken, resulting in a yellow or orange clear solution depending on the complex used. The obtained cationic complexes (**$2Zr_a$-$2Zr_d$**; **$2Hf_a$-$2Hf_d$**) were characterized by low-temperature NMR spectroscopy. NMR data of the zirconium derivative were reported before.[4] NMR data of the hafnium complexes are summarized below. C_{ipso}, C_{ortho}, C_{meta} and C_{para} refer to the carbon atoms of the benzyl moiety.

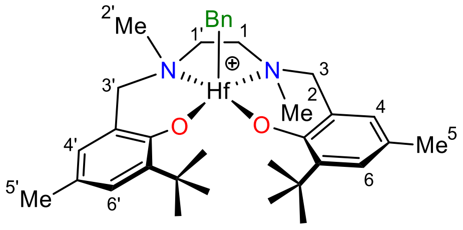	**$2Hf_a$:** ^{1}H-NMR (C_6D_5Cl, 273K, δ in ppm, J in Hz): δ = 7.27 (d, H6'), 7.21 (d, H6), 6.97 (m, H_{meta}), 6.87 (m, H_{para}), 6.75 (d, H_{ortho}), 6.66 (d, H4'), 6.48 (d, H4), 3.45 (d, H3'), 3.24 (d,H3'), 2.93 (d, H3), 2.76 (d, H1'), 2.70 (d, H1), 2.57 (d, H1'), 2.53 (d, Bn), 2.32 (d, H3), 2.27 (s, H5'), 2.23 (d, H1), 2.18 (s, H5), 2.17 (s, H2'), 1.99 (d, Bn), 1.75 (s, H2), 1.55 (s, H ^{t}Bu), 1.49 (s, H ^{t}Bu'). ^{13}C-NMR (C_6D_5Cl, 273K, δ in ppm): δ = 154.8 (s, C_{quat}), 153.3 (s, C_{quat}), 138.1 (s, C_{quat}), 138.1 (s, C_{ipso}), 134.8 (s, C_{ortho}), 132.5 (s, C_{quat}), 132.3 (s, C_{quat}), 130.6 (s, C6'), 129.5 (s, C6), 129.3 (s, C4'), 128.2 (s, C4), 126.1 (s, C_{para}), 122.5 (s, C_{quat}), 121.4 (s, C_{quat}), 75.8 (s, C Bn), 65.4 (s, C3'), 62.5 (s, C3), 57.1 (s, C1), 55.8 (s, C1'), 42.9 (s, C2'), 37.7 (s, C2), 34.9 (s, C ^{t}Bu/^{t}Bu'), 30.0 (s, C ^{t}Bu'), 29.9 (s, C ^{t}Bu), 20.8 (s, C5), 20.8 (s, C5').

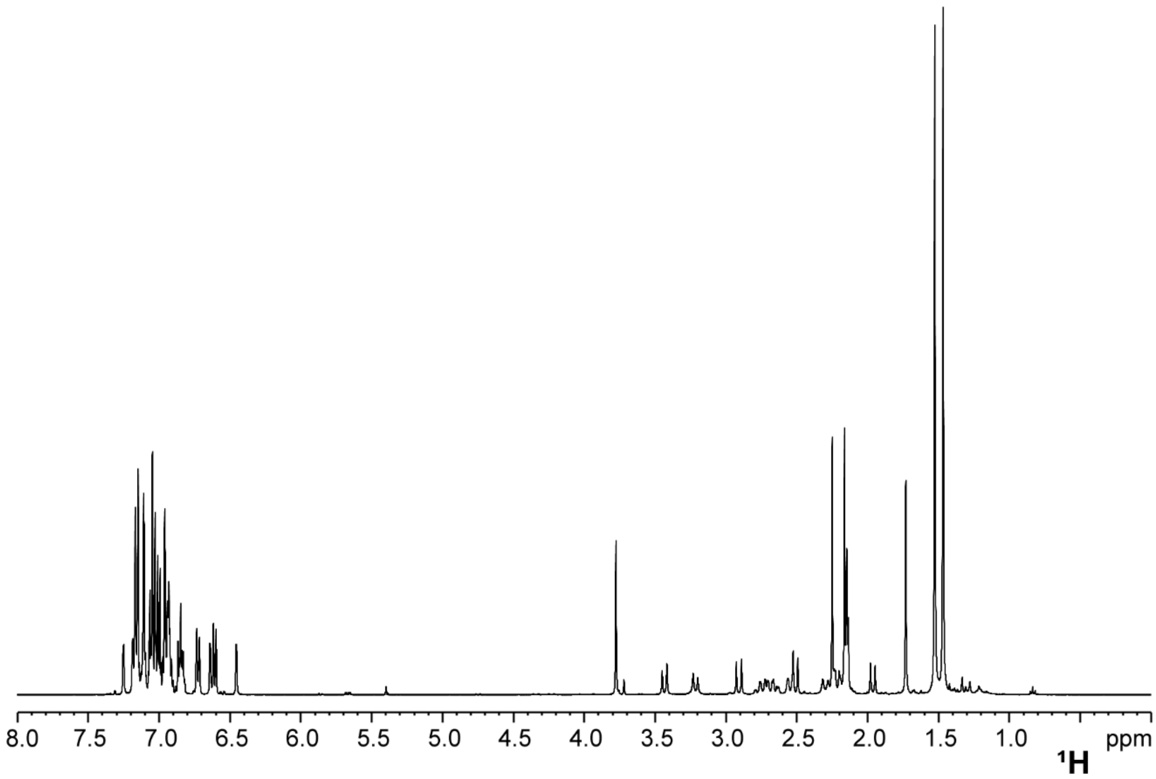

Figure S5. ^{1}H NMR spectrum of **$2Hf_a$** (C_6D_5Cl, 273 K).

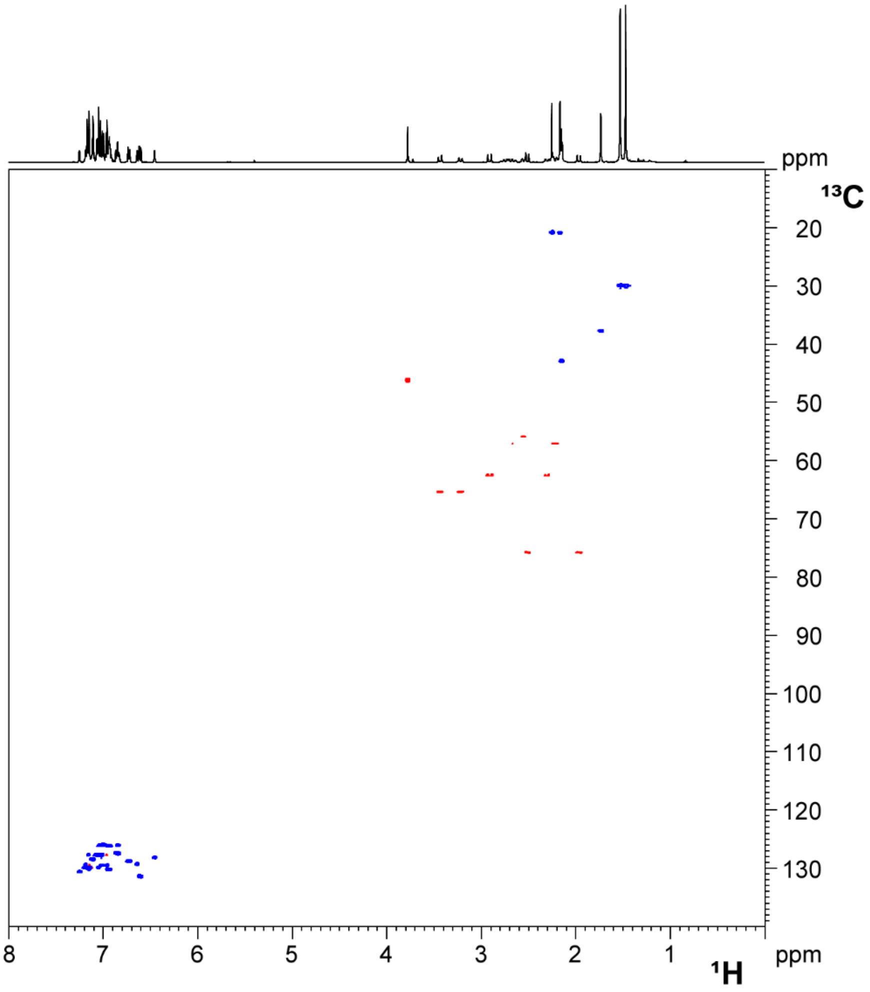

Figure S6. 1H 13C HSQC NMR spectrum of **2Hf$_a$** (C_6D_5Cl, 273 K).

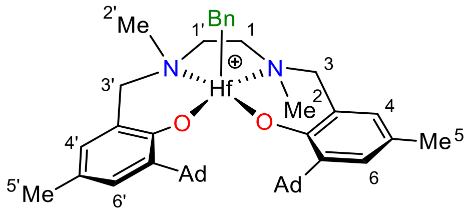	**$2Hf_b$:** ^1H-NMR (C_6D_5Cl, 268K, δ in ppm, J in Hz): δ = 6.57 (s, H4’), 6.50 (s, H4), 3.28 (d, J_{HH} = 13.8 Hz, H3’), 3.18 (s broad, H3), 3.07 (d, J_{HH} = 14.6 Hz, H3), 2.78 (m, H1), 2.66 (H Bn), 2.62 (d, H1), 2.38 (d, H1), 2.29 (s, H5’ + H Bn), 2.25 (s, H5), 2.05 (H2’+ Ad), 1.91 (s, H2).<br>^{13}C-NMR (C_6D_5Cl, 268K, δ in ppm): δ = 128.8 (C4’), 127.8 (C4), 77.4 (C Bn), 62.9 (C3’), 61.4(C3), 56.8 (C1), 42.1 (C2’), 38.3 (C2), 20.5 (C5), 20.4 (C5’).

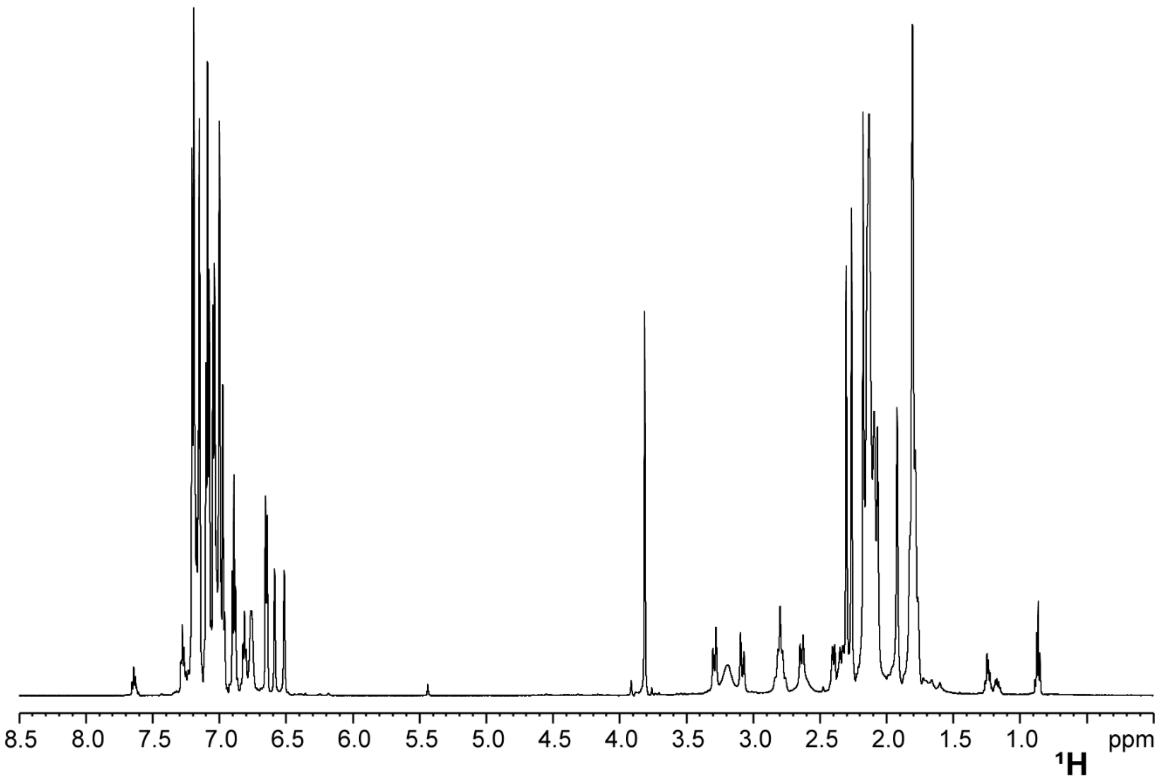

Figure S7. ^1H NMR spectrum of **$2Hf_b$** (C_6D_5Cl, 268 K).

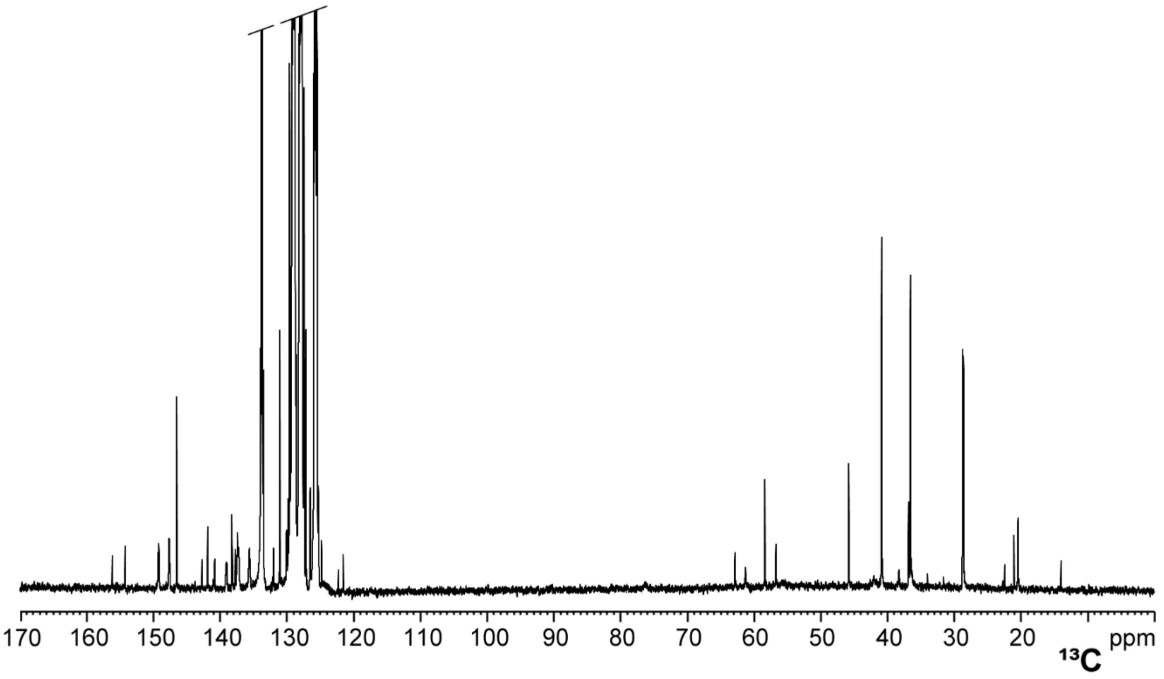

Figure S8. ^{13}C NMR spectrum of **$2Hf_b$** (C_6D_5Cl, 268 K).

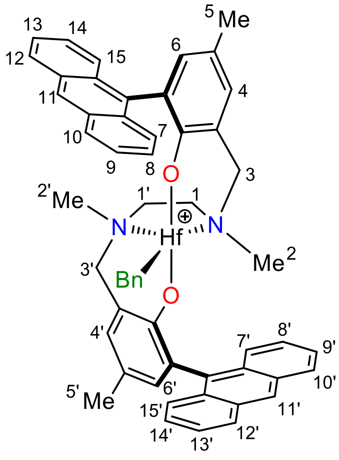	**2Hf$_c$:** ^{1}H-NMR (C_6D_5Cl, 233K, δ in ppm, J in Hz): δ = 6.93 (m, H6'), 6.91 (m, H6), 6.81 (m, H4'), 6.50 (m, H4), 6.30 (m, H$_{para}$'), 5.92 (m, H$_{ortho}$'), 5.73 (m, H$_{meta}$), 5.60 (m, H$_{meta}$'), 4.06 (m, H3' e H$_{ortho}$), 3.04 (d, H3'), 2.98 (d, H1'), 2.59 (d, H1b), 2.32 (s, H5'), 2.31 (d, H3), 2.21 (s, H5), 2.20 (d, H3), 1.97 (s, H2'), 1.93 (d, H1'), 1.86 (d, Bn), 1.48 (d, H1), 1.42 (s, H2), 0.69 (d, Bn).<br>^{13}C-NMR (C_6D_5Cl, 233K, δ in ppm): δ = 153.9 (s, C$_{quat}$), 153.4 (s, C$_{quat}$), 136.4 (s, C$_{ortho}$), 135.2 (s, C'$_{ortho}$), 133.3 (s, C6'), 132.9 (s, H6), 130.7 (s, C4'), 130.1 (s, C'$_{meta}$), 130.1 (s, C4), 130.0 (s, H$_{meta}$), 129.9 (s, C$_{quat}$), 128.8 (s, C'$_{ipso}$), 70.2 (s, C Bn), 64.9 (s, C3'), 61.8 (s, C3), 54.7 (s, C1'), 51.4 (s, C1), 47.5 (s, C2'), 45.9 (s, C2), 20.6 (s, C5'), 20.4 (s, C5).

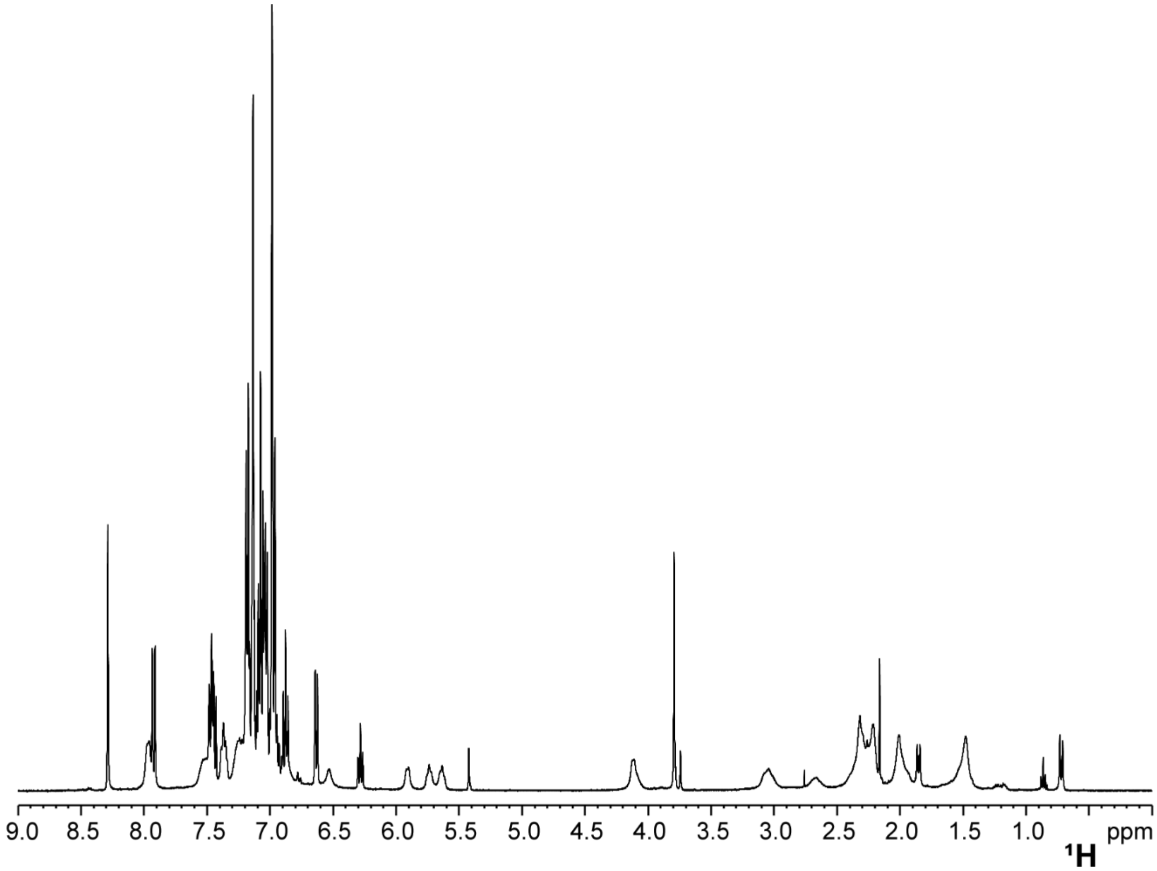

Figure S9. ^{1}H NMR spectrum of **2Hf$_c$** (C_6D_5Cl, 233 K).

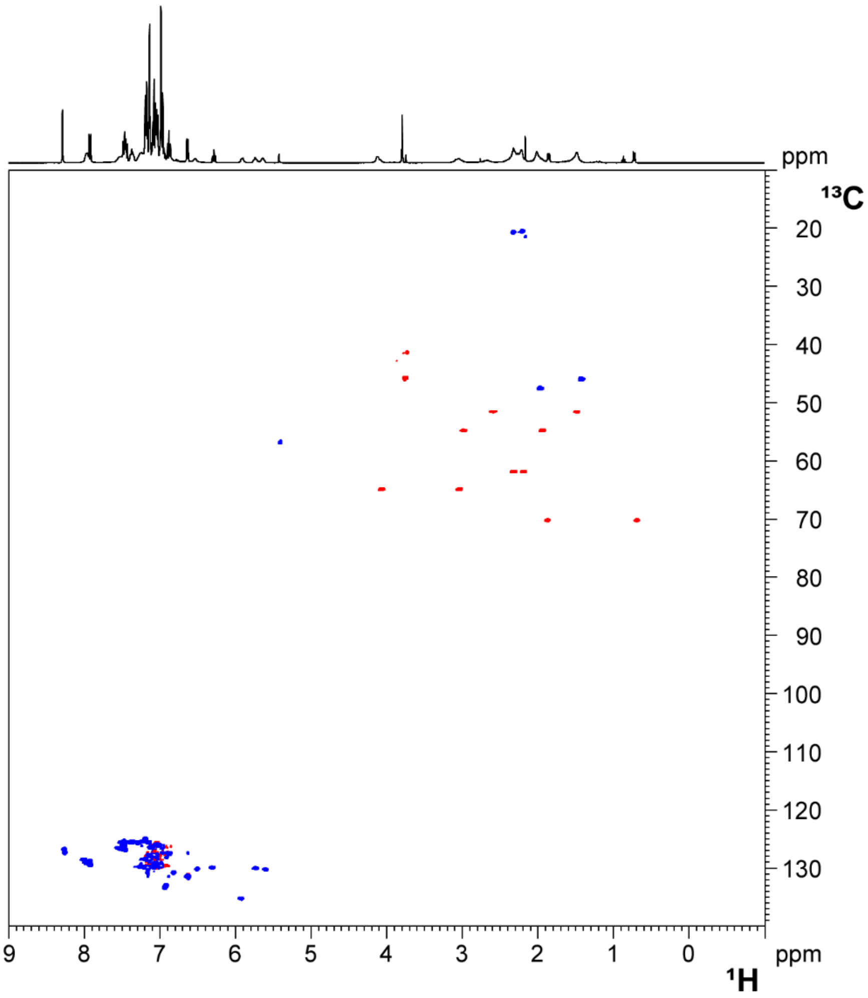

Figure S10. ^{1}H ^{13}C HSQC NMR spectrum of **2Hf$_c$** (C_6D_5Cl, 233 K).

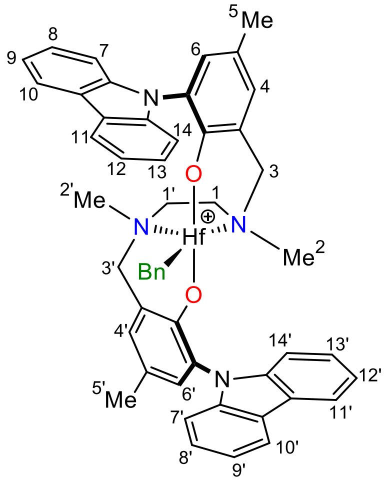	**2Hf$_d$**: ^{1}H-NMR (C_6D_5Cl, 233K, δ in ppm, J in Hz): δ = 6.84 (s, H4'), 6.50 (s, H4), 6.39 (t, H_{para}'), 6.00 (H_{meta}), 5.92 (H_{ortho}), 5.81 (t, H_{meta}'), 4.65 (H_{ortho}'), 4.08 (d, H3'), 3.09 (d, H3'), 2.95 (H1'), 2.58 (t, H1), 2.44 (H3), 2.34 (H3), 2.30 (s, H5), 2.19 (s, H5'), 2.03 (s, H2'), 1.99 (H1'), 1.97 (Bn), 1.56 (s, H2 + H1), 1.02 (d, Bn).<br>^{13}C-NMR (C_6D_5Cl, 233K, δ in ppm): δ = 136.6 (s, C_{ortho}), 135.4(C_{ortho}'), 131.5 (s, C4), 131.1 (s, C6), 130.8 (C_{meta}'), 130.7 (C_{meta}), 130.5 (s, C_{para}), 71.5 (s, C Bn), 64.6 (s, C3'), 61.6 (C3), 54.8 (s, C1'), 51.4 (s, C1), 47.4 (s, C2'), 45.6 (s, C2), 20.3 (s, C5), 20.1 (s, C5').

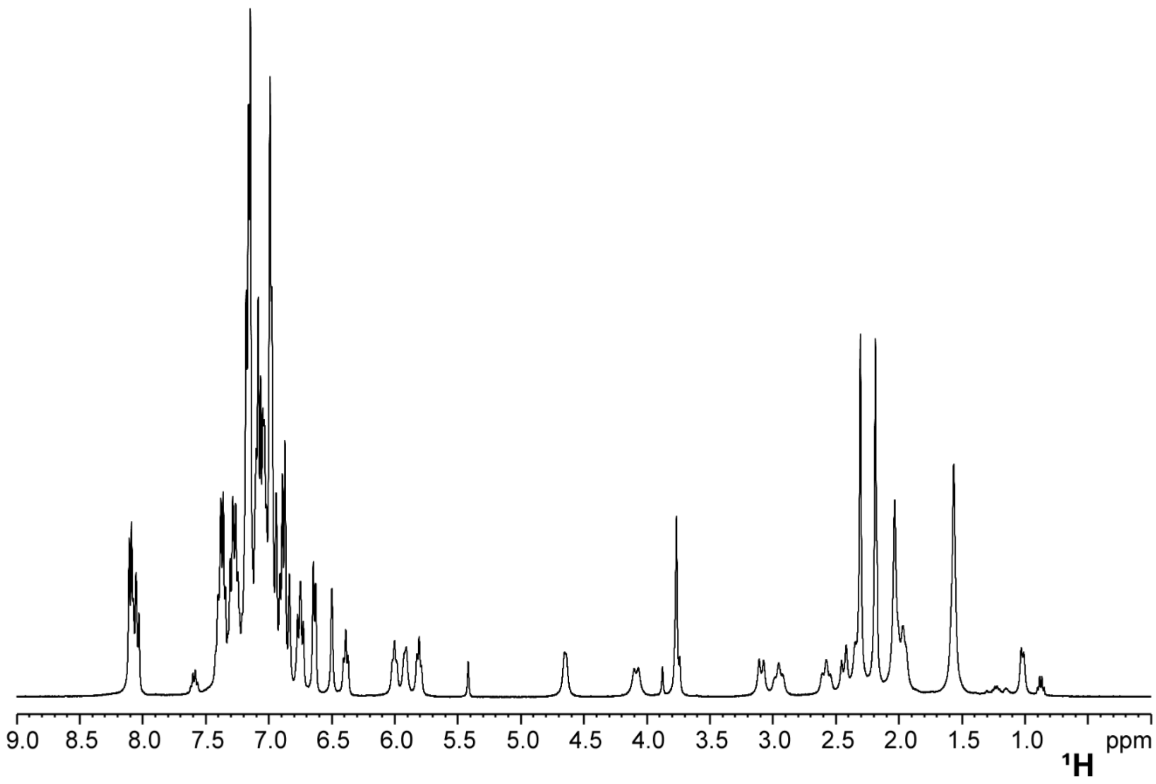

Figure S11. ^{1}H NMR spectrum of **2Hf$_d$** (C_6D_5Cl, 233 K).

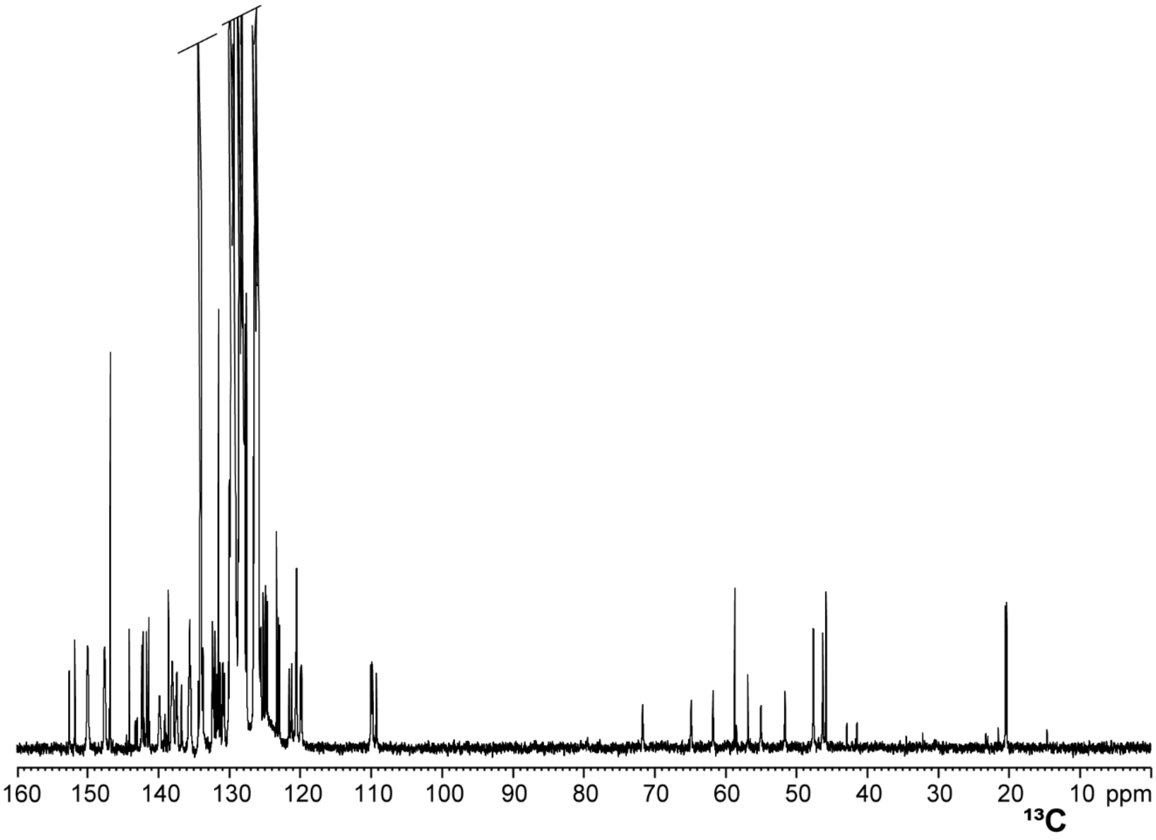

Figure S12. ^{13}C NMR spectrum of **$2Hf_d$** (C_6D_5Cl, 233 K).

General procedure for the generation of heterobimetallic adducts.

In the glovebox, the desired amount of $AlMe_3$ or $ZnMe_2$ was injected directly into a precooled solution of cationic complex within a J-Young NMR tube. The mixture was shaken and left to reach 298 K within a few minutes. The NMR tube was taken outside the glove box and transferred into the precooled probe of the NMR spectrometer for analysis. NMR data of the complexes (**3Zr$_a$**;**3Zr$_c$**; **3Hf$_a$**-**3Hf$_d$**; **4Hf$_b$**; **4Hf$_d$**) are summarized below.

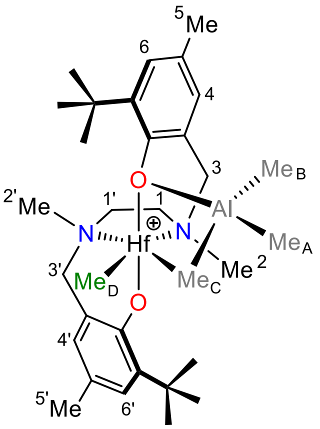	**3Hf$_a$:** ^{1}H-NMR (C_6D_5Cl, 253K, δ in ppm, J in Hz): δ = 7.39 (s, H6), 7.22 (H6'), 6.53 (s, H4'), 3.97 (d, J_{HH} = 14.3 Hz, H3'), 3.77 (H3), 2.96 (d, 14.6 Hz, H3'), 2.89 (d, J_{HH} = 13.7 Hz, H3), 2.65 (H1), 2.59 (H1'), 2.23 (s, H5'), 2.12 (s, H5), 1.91 (s, H2), 1.77 (s, H2'), 1.62 (s, H1), 1.55 (H1'), 1.40 (^{t}Bu/^{t}Bu'), 1.39 (^{t}Bu/^{t}Bu'), 1.38 (Me$_C$), 0.74 (s, Me$_D$), -0.28 (s, Me$_A$), -0.80 (s, Me$_B$). ^{13}C-NMR (C_6D_5Cl, 253K, δ in ppm): δ = 64.8 (C3'), 62.4 (C3), 58.3 (C$_D$), 54.6 (C1), 51.5 (C1'), 47.2 (C2'), 42.4 (C2'), 31.9 (C ^{t}Bu/^{t}Bu'), 29.9 (C$_C$), 20.8 (C5'), 20.4 (C5), -3.5 (C$_A$), -10.7 (C$_B$).

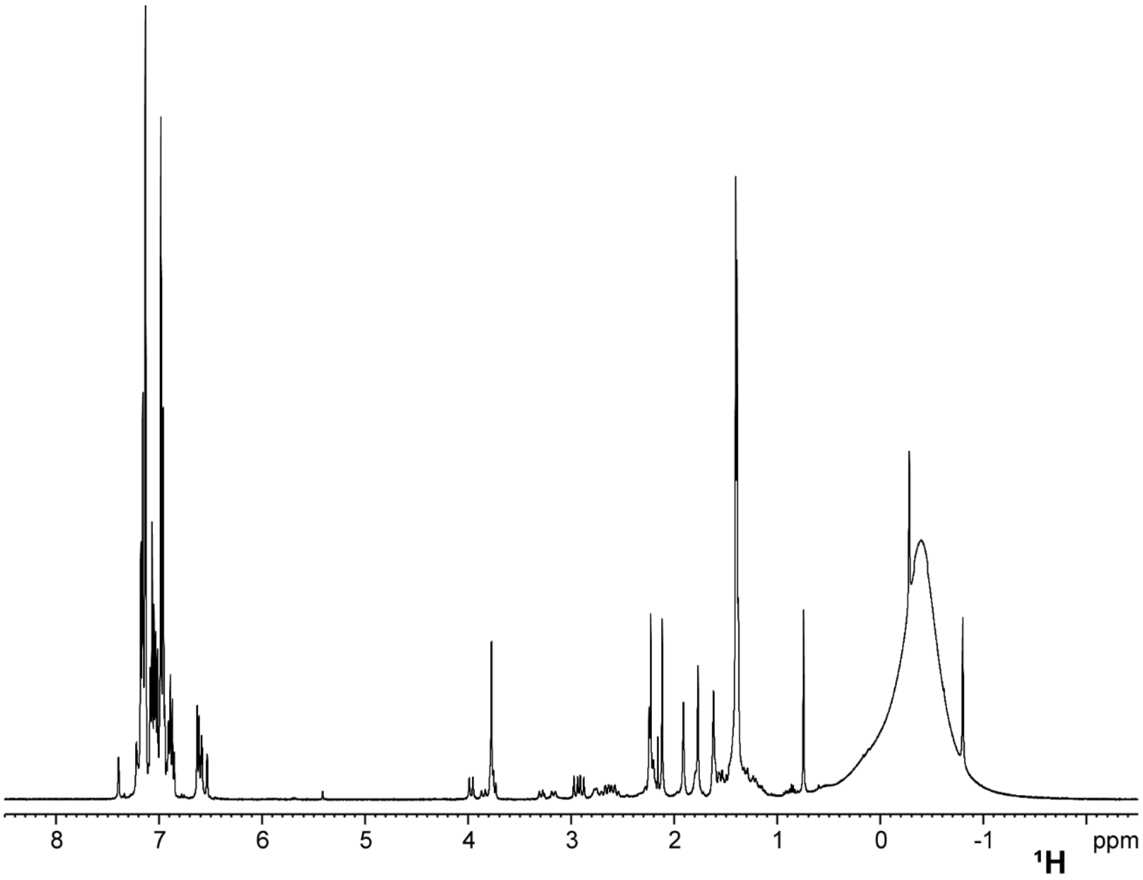

Figure S13. ^{1}H NMR spectrum of **3Hf$_a$** (C_6D_5Cl, 253 K).

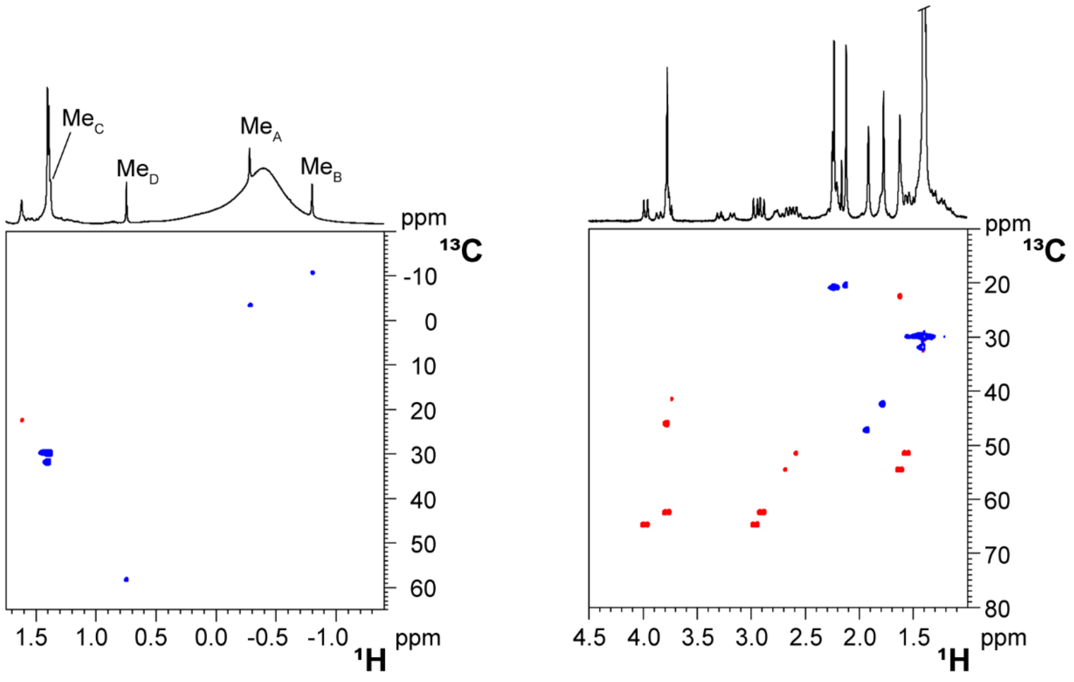

Figure S14. Sections of ^{1}H ^{13}C HSQC NMR spectrum of **3Hf$_a$** (C_6D_5Cl, 253 K).

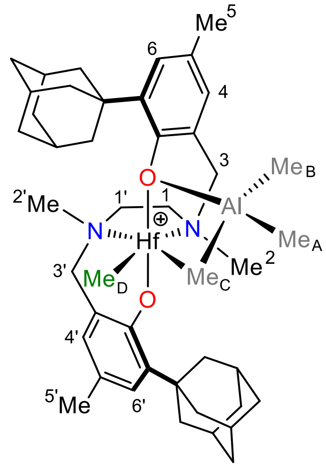

3Hf$_b$: ^{1}H-NMR (C_6D_5Cl, 233K, δ in ppm, J in Hz): δ = 7.31 (s, H6), 6.59 (s, H4), 6.55 (s, H4'), 4.00 (d, J_{HH} = 14.1 Hz, H3'), 3.87 (d, J_{HH} = 13.1 Hz, H3), 2.94 (H3'), 2.90 (H3), 2.67 (H1), 2.54 (H1'), 2.30 (s, H5'), 2.16 (s, H5), 2.03 (H2 + Ad), 1.85 (H2' + Ad), 1.65 (s, H_C), 1.61 (H1), 1.54 (H1'), 0.89 (s, H_D), -0.26 (s, H_A), -0.80 (s, H_B).

^{13}C-NMR (C_6D_5Cl, 233K, δ in ppm): δ = 132.2 (C6), 131.5 (C4), 129.0 (C4'), 64.4 (C3'), 62.6 (C3), 57.7 (C_D), 54.2 (C1), 51.5 (C1'), 47.3 (C2), 43.0 (C2'), 28.6 (C_C), 20.8 (C5'), 20.4 (C5), -3.0 (C_A), -10.8 (C_B).

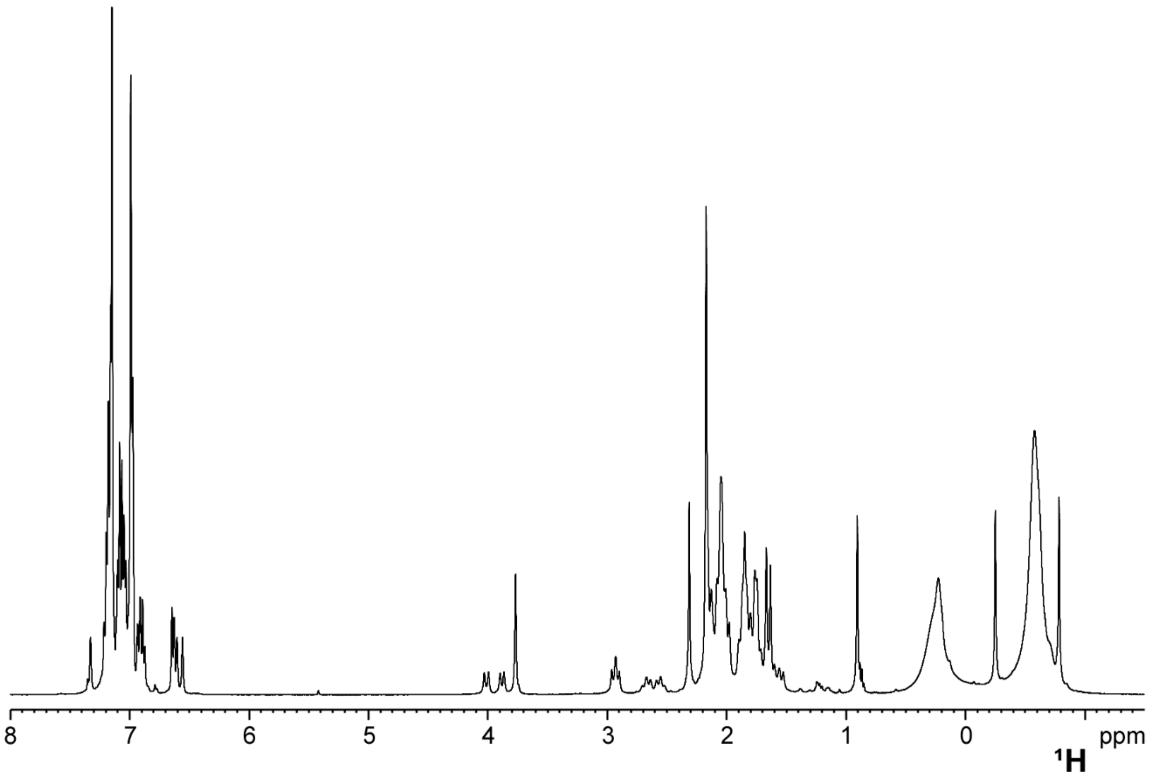

Figure S15. ^{1}H NMR spectrum of **3Hf$_b$** (C_6D_5Cl, 233 K).

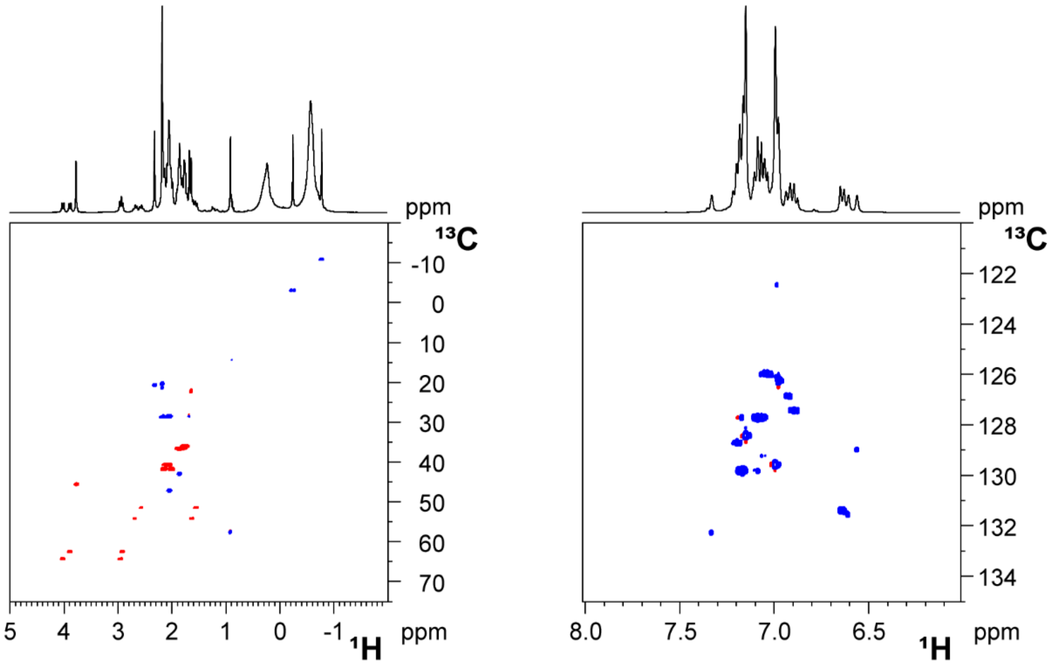

Figure S16. Sections of ^{1}H ^{13}C HSQC NMR spectrum of **$3Hf_b$** (C_6D_5Cl, 233 K).

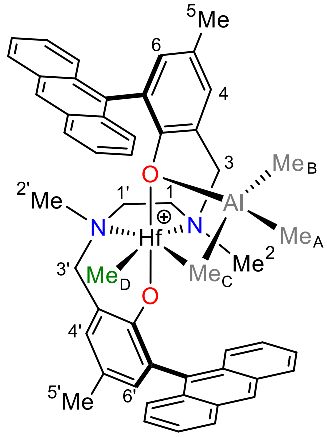

3Hf$_c$: ^{1}H-NMR (C_6D_5Cl, 233K, δ in ppm, J in Hz): δ = 8.35 (s, H11/H11’), 8.24 (s, H11/H11’), 6.79 (s, H4’), 4.15 (d, J_{HH} = 14.4 Hz, H3’), 3.25 (d, J_{HH} = 13.4 Hz, H3), 3.15 (d, J_{HH} = 14.5 Hz, H3’), 2.86 (H1 e H1’), 2.71 (H3), 2.31 (s, H5/H5’), 2.17 (s, H5/H5’), 1.99 (s, H2’), 1.81 (H1 e H1’), 1.29 (s, H2), -0.23 (s, Me$_D$), -0.31 (s, Me$_C$), -1.19 (s, Me$_B$), -2.59 (s, Me$_A$).

^{13}C-NMR (C_6D_5Cl, 233K, δ in ppm): δ = 64.1 (s, C3’), 61.3 (s, C3), 58.2 (s, C$_D$), 53.3 (s, C1/C1’), 52.1 (s, C1/C1’), 45.1 (s, C2), 43.5 (s, C2’), 24.0 (s, C$_C$), 20.8 (s, C5/C5’), 20.4 (s, C5/C5’), -10.0 (s, C$_B$), -10.1 (s, C$_A$).

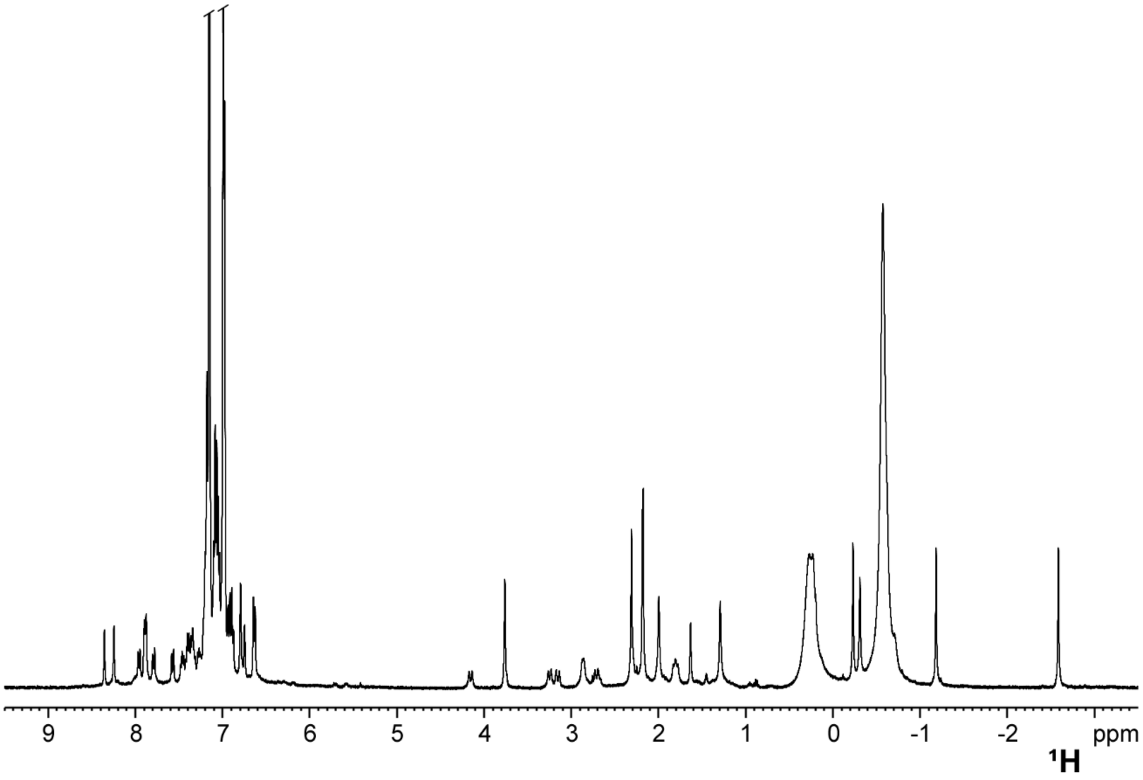

Figure S17. ^{1}H NMR spectrum of **3Hf$_c$** (C_6D_5Cl, 233 K).

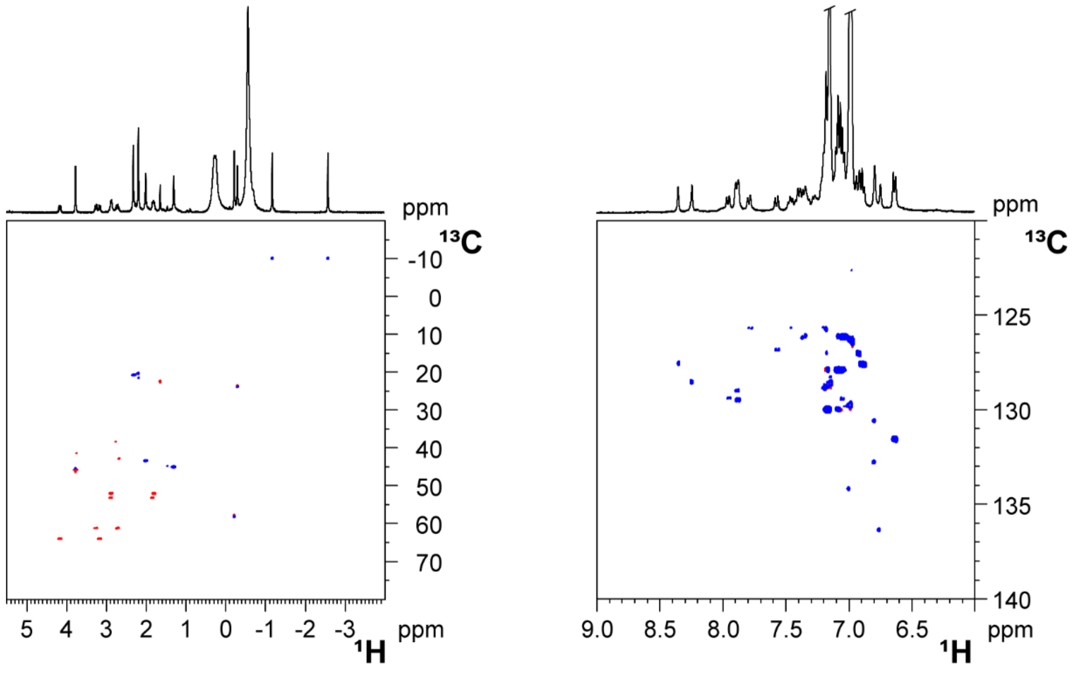

Figure S18. Sections of ^{1}H ^{13}C HSQC NMR spectrum of **$3Hf_c$** (C_6D_5Cl, 233 K).

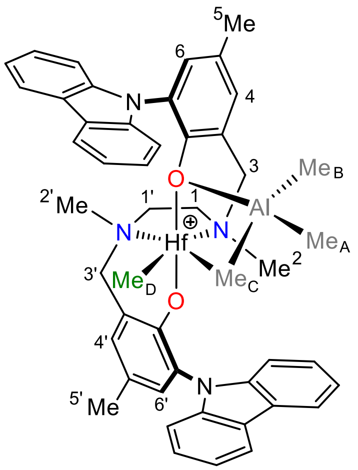	**$3Hf_d$**: ^{1}H-NMR (C_6D_5Cl, 233K, δ in ppm, J in Hz): δ = 7.06 (H6), 6.90 (H4), 4.17 (d, J_{HH}= 14.3 Hz, H3’), 3.24 (d, J_{HH}= 13.5 Hz, H3), 3.15 (d, J_{HH}= 14.6 Hz, H3’), 2.82 (H1), 2.76 (H1’), 2.72 (H3), 2.30 (s, H5), 2.11 (s, H2’), 2.08 (s, H5’), 1.85 (H1’), 1.73 (d, J_{HH}= 11.7 Hz, H1), 1.24 (s, H2), 0.32 (s, Me_D), 0.08 (s, Me_C), -1.08 (s, Me_B), -1.93 (s, Me_A). ^{13}C-NMR (C_6D_5Cl, 233K, δ in ppm): δ = 110.8 (C6), 110.1 (C4), 63.6 (s, C3’), 60.9 (C3), 58.3 (s, C_D), 53.0 (s, C1’), 52.1 (s, C1), 44.4 (s, C2), 43.2 (s, C2’), 28.4 (s, C_C), 20.5 (s, C5), 19.9 (s, C5’), -9.9 (s, C_A), -10.5 (s, C_B).

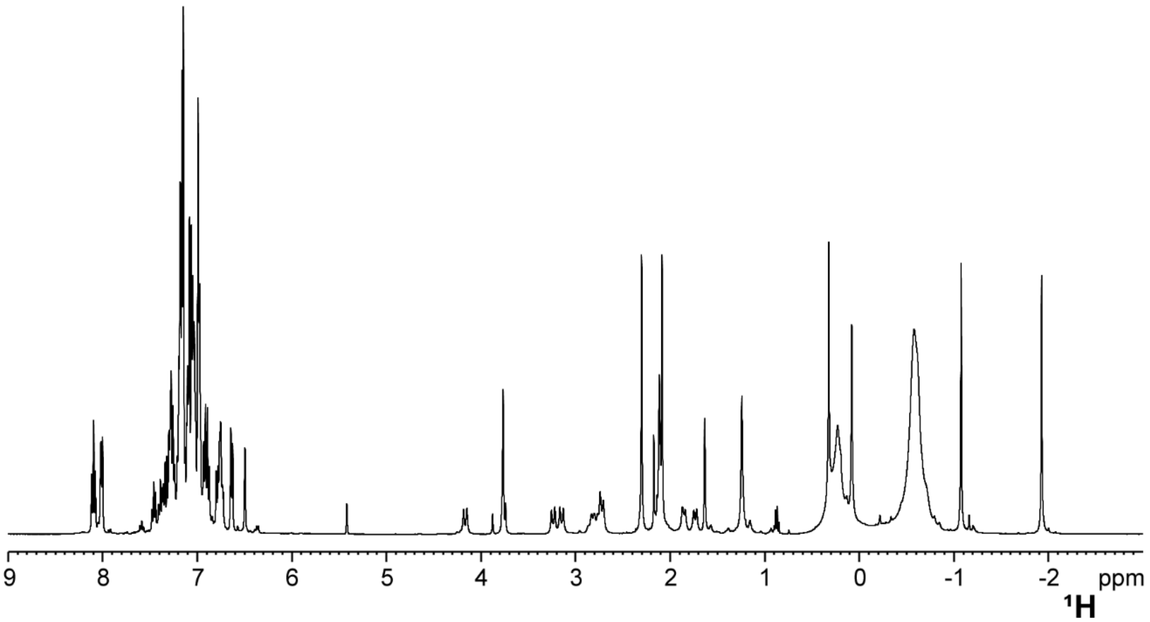

Figure S19. ^{1}H NMR spectrum of **$3Hf_d$** (C_6D_5Cl, 233 K).

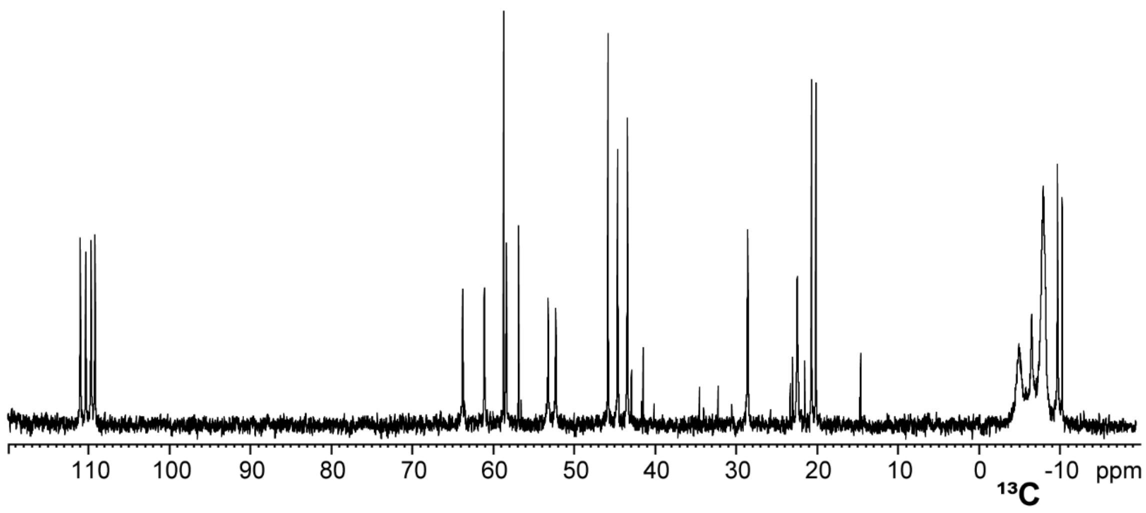

Figure S20. ^{13}C NMR spectrum of **$3Hf_d$** (C_6D_5Cl, 233 K).

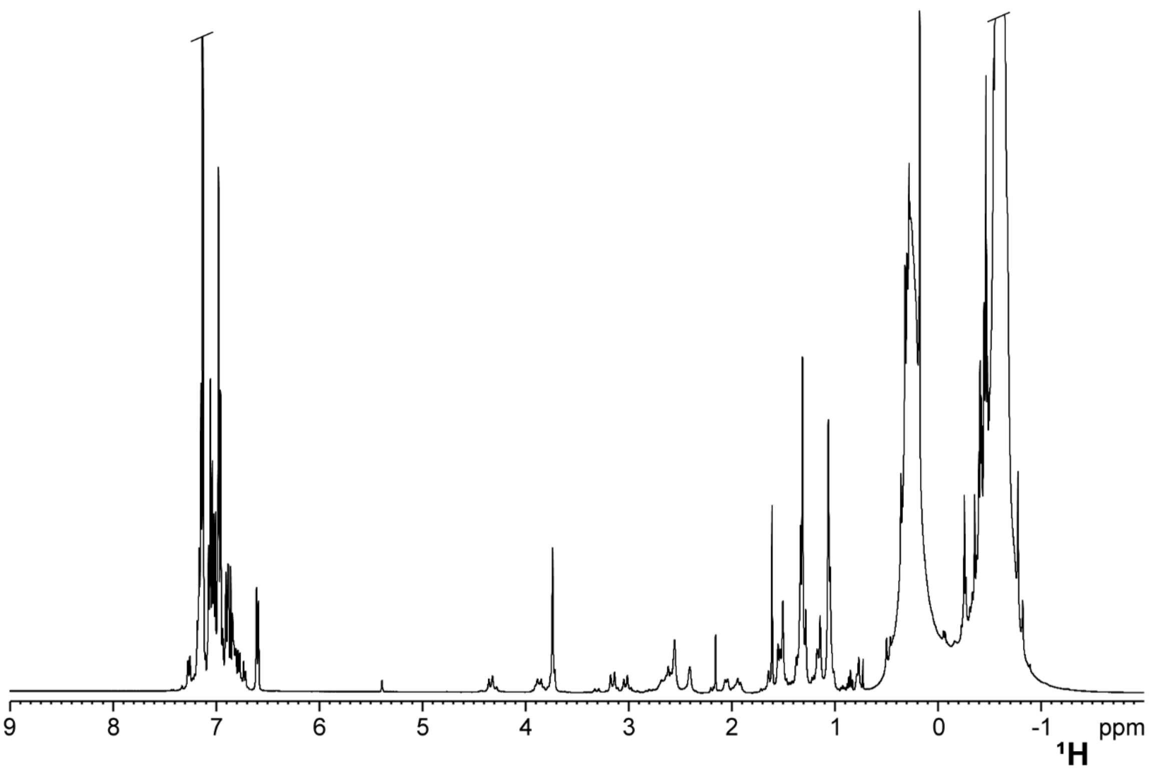

Figure S21. ^{1}H NMR spectrum of the mixture obtained from the reaction of **2Zr$_a$** with $AlMe_3$ (C_6D_5Cl, 233 K).

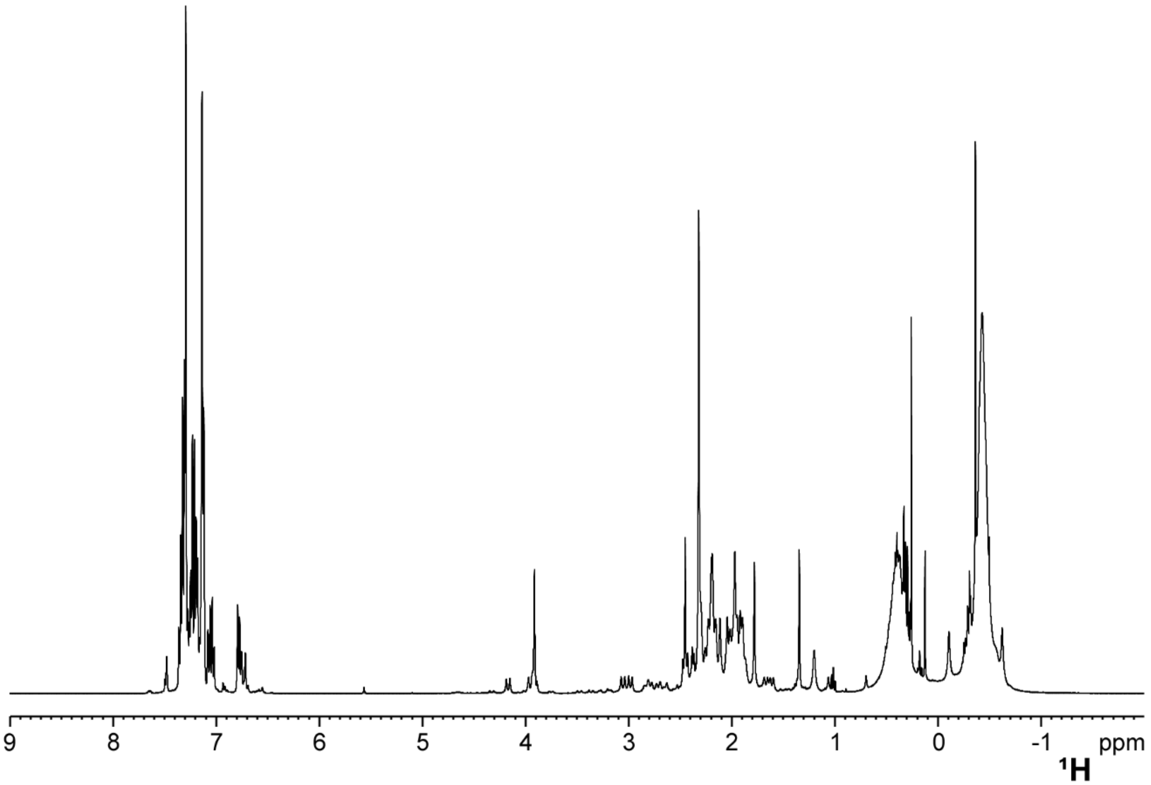

Figure S22. ¹H NMR spectrum of the mixture obtained from the reaction of **$2Zr_b$** with $AlMe_3$ (C_6D_5Cl, 233 K).

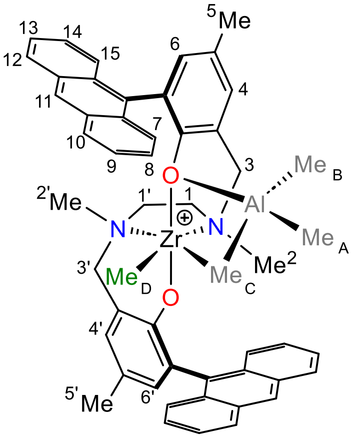

$3Zr_c$: ^{1}H-NMR (C_6D_5Cl, 233K, δ in ppm, J in Hz): δ = 5.90 (d, J_{HH} = 7.5 Hz, H4/H4'), 4.19 (d, J_{HH} = 14.4 Hz, H3'), 3.20 (d, J_{HH} = 13.3 Hz, H3), 3.14 (d, J_{HH} = 14.4 Hz, H3'), 1.88 (m, H1+H1'), 2.63 (m, H3), 2.31 (s, H5), 2.18 (s, H5'), 2.00 (s, H2'), 1.81 (m, H1/H1'), 1.71 (m, H1/H1'), 1.23 (s, H2), 0.12 (s, Me_D), -0.87 (s, Me_C), -1.17 (s, Me_B), -2.61 (s, Me_A).

^{13}C-NMR (C_6D_5Cl, 233K, δ in ppm): δ = 64.3 (C3'), 61.3 (C3), 61.2 (C_D), 53.3 (C1/C1'), 51.7 (C1/C1'), 44.6 (C2), 43.6 (C2'), 20.8 (C5), 20.4 (C5'), 18.8 (C_C), -9.8 (C_B), -10.2 (C_A).

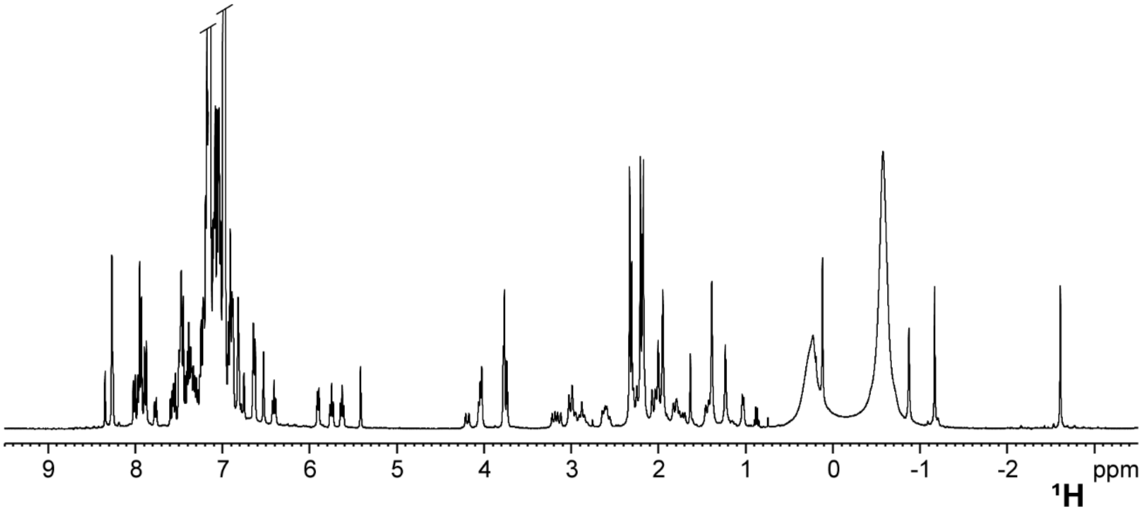

Figure S23. ^{1}H NMR spectrum of the mixture obtained from the reaction of **$2Zr_c$** with $AlMe_3$ (C_6D_5Cl, 233 K).

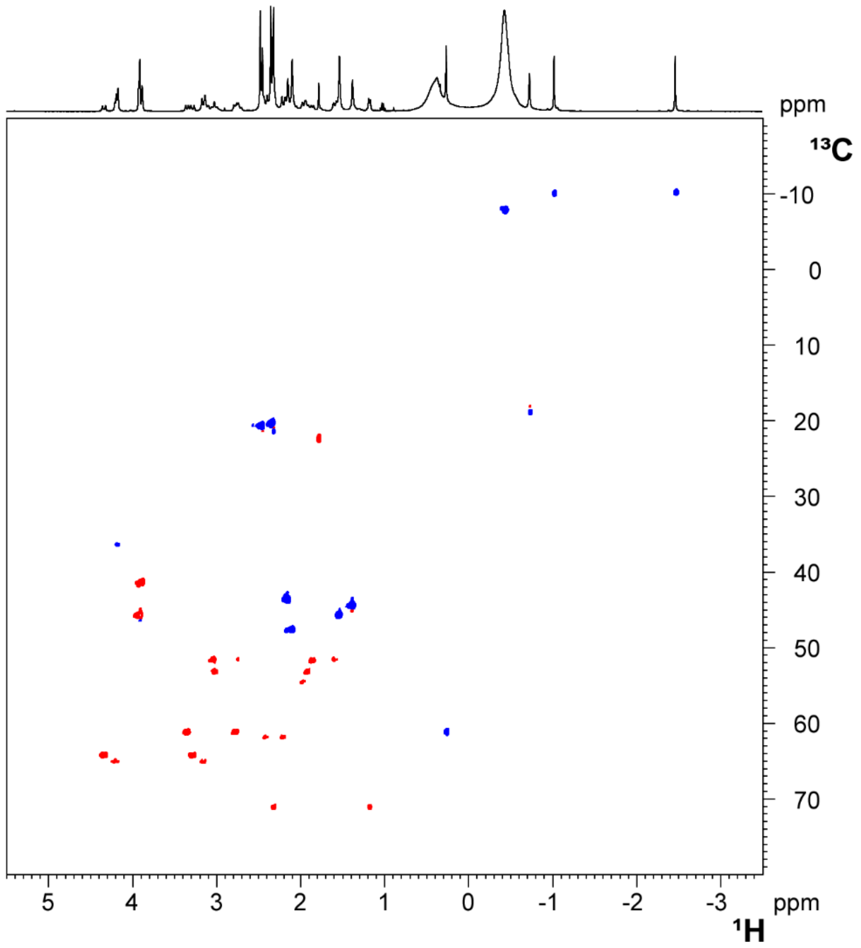

Figure S24. Section of ^{1}H ^{13}C HSQC NMR spectrum of the mixture obtained from the reaction of **2Zr$_c$** with $AlMe_3$ (C_6D_5Cl, 233 K).

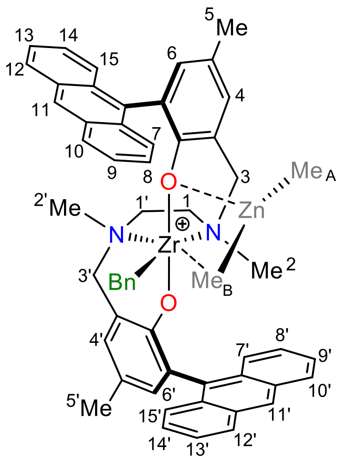

4Zr$_c$: ^{1}H-NMR (C_6D_5Cl, 228K, δ in ppm, J in Hz): 3.86 (d, J_{HH} = 14.1 Hz, H3'), 3.09 (J_{HH} = 14.5 Hz, H3), 2.92 (H1/H1'), 2.82 (d, J_{HH} = 14.1 Hz, H3'), 2.72 (H1/H1'), 2.62 (d, J_{HH} = 14.5 Hz, H3), 2.24 (s, H5/H5'), 2.09 (s, H5/H5'+toluene), 1.93 (Bn), 1.80 (s, H2'), 1.67 (H1/H1'), 1.44 (H1/H1'), 1.25 (s, H2), 0.85 (d, J_{HH} = 6.4 Hz, Bn), -0.21 (s, Me$_A$), -3.55 (s, Me$_B$).

^{13}C-NMR (C_6D_5Cl, 233K, δ in ppm): δ = 68.0 (C$_{ab}$), 65.0 (C3'), 63.2 (C3), 54.5 (C1/C1'), 54.1 (C1/C1'), 47.8 (C2'), 46.0 (C2), 21.5 (C5/C5'), 20.8 (C5/C5'), -4.4 (C$_A$), -14.6 (C$_B$).

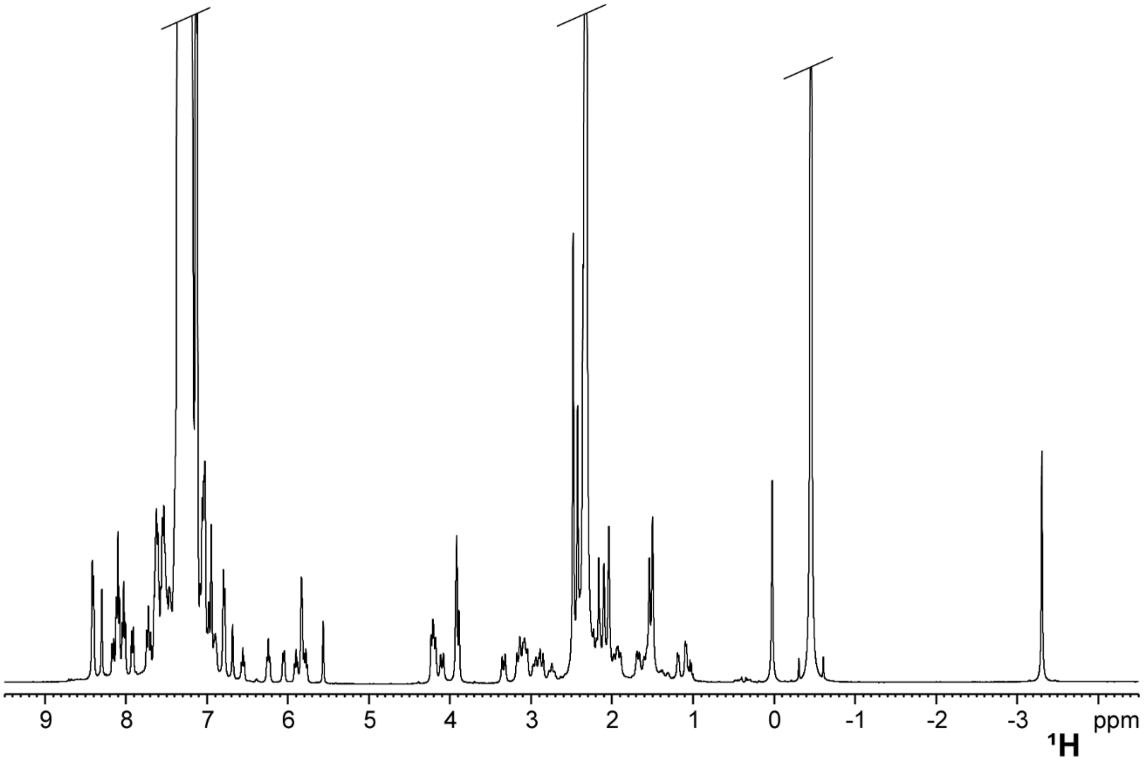

Figure S25. ^{1}H NMR spectrum of **4Zr$_c$** (C_6D_5Cl, 228 K).

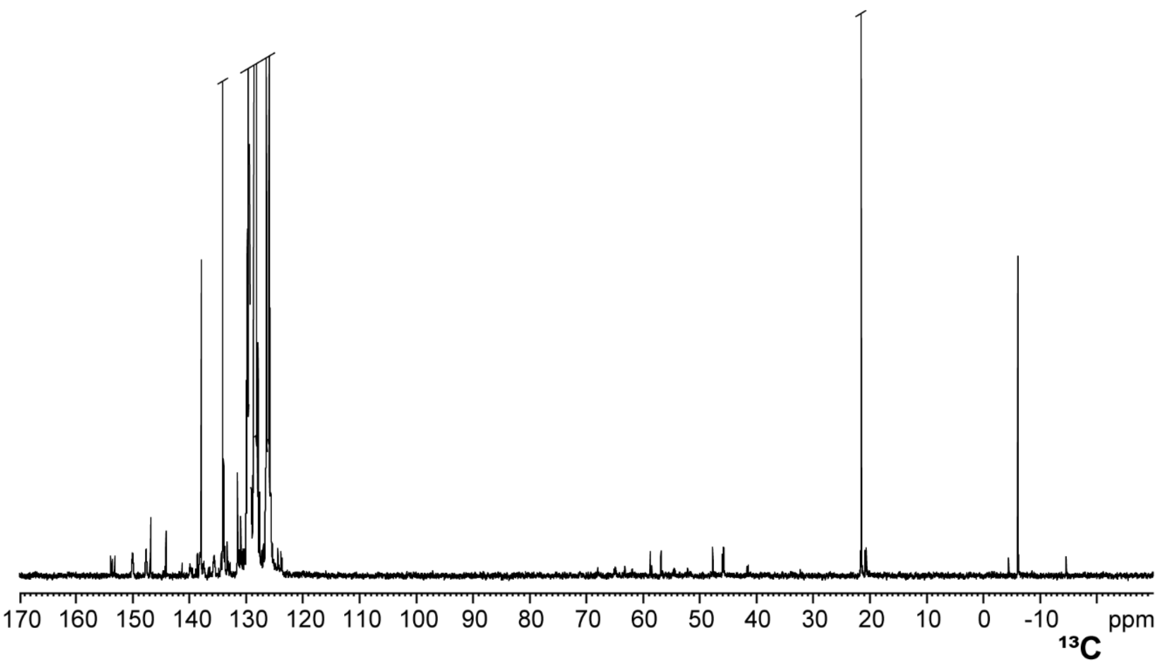

Figure S26. ^{13}C NMR spectrum of **$4Zr_c$** (C_6D_5Cl, 228 K).

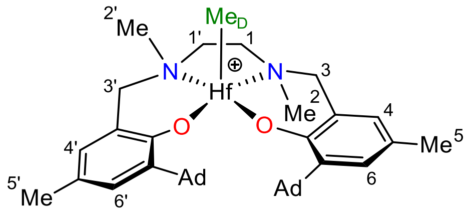	**$4Hf_b$:** ^{1}H-NMR (C_6D_5Cl, 233K, δ in ppm, J in Hz): δ = 6.61(s, H4), 6.39 (s, H4'), 4.00 (d, H3), 3.15 (d, H3), 3.02 (d, H3'), 2.81 (d, H3'), 2.64 (H1'), 2.51 (H1), 2.42 (H1'), 2.33 (H1), 2.27 (H5), 2.22 (H5'), 1.84 (H2), 1.66 (H2'), 0.49 (s, Me_D).<br>^{13}C-NMR (C_6D_5Cl, 233K, δ in ppm): δ = 129.0 (s, C4'), 128.7 8s, C4), 62.6 (s, C3'), 59.3 (s, C3), 56.3 (s, C1'), 56.1 (s, C1), 51.4 (s, Me_D), 41.6 (s, C2'), 38.1 (s, C2), 21.0 (s, C5), 20.9 (s, C5').

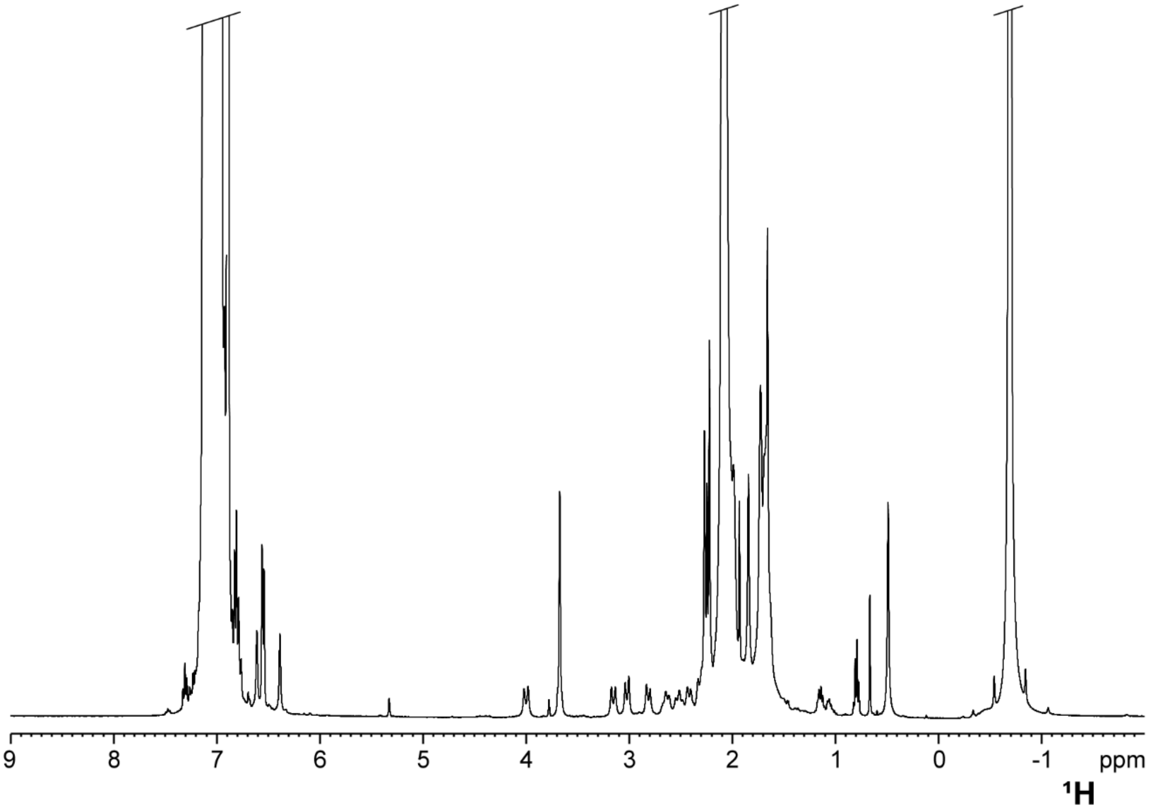

Figure S27. ^{1}H NMR spectrum of $\mathbf{4Hf_b}$ (C_6D_5Cl, 228 K).

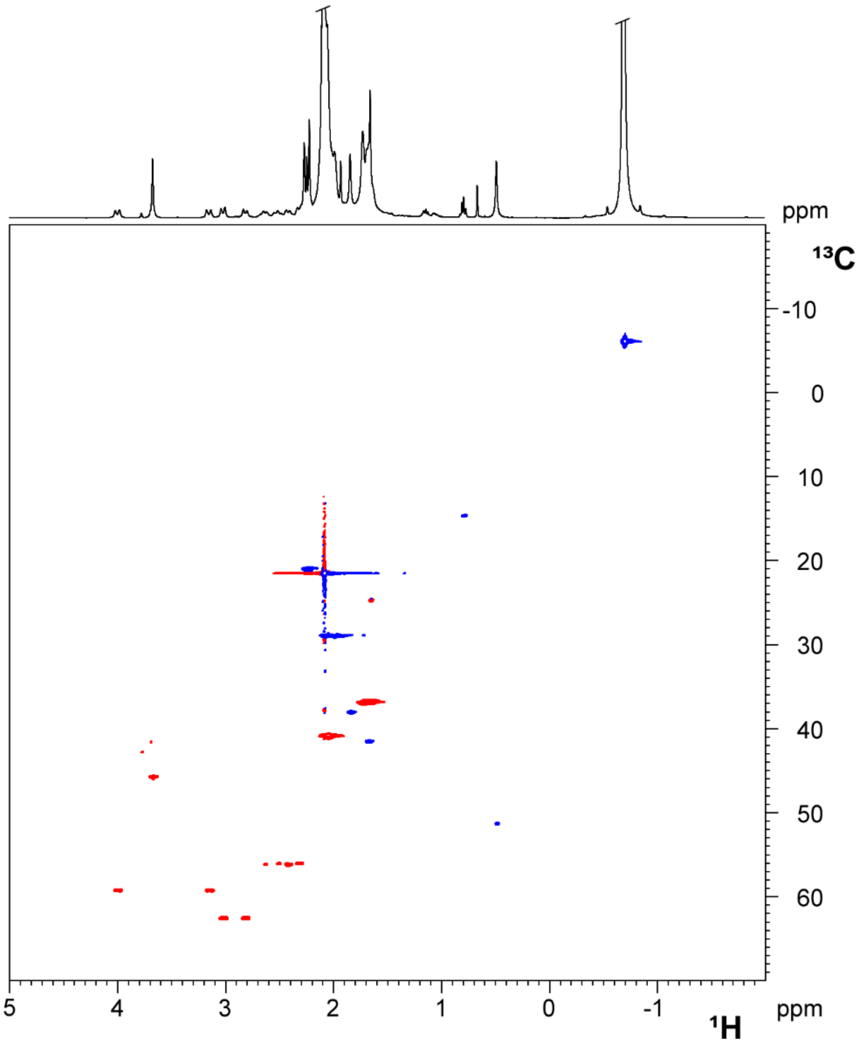

Figure S28. Section of ¹H ¹³C HSQC NMR spectrum of **$4Hf_b$** (C_6D_5Cl, 228 K).

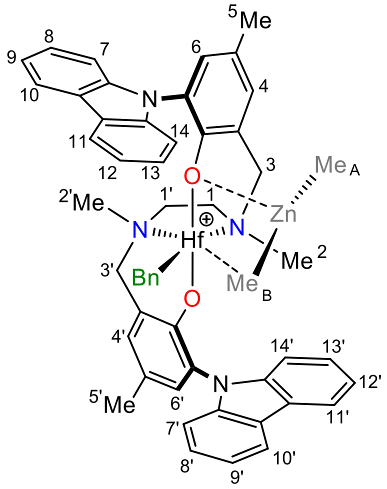 	**4Hf$_d$**: ^{1}H-NMR (C_6D_5Cl, 233K, δ in ppm, J in Hz): δ = = 3.88 (d, J_{HH} = 14.5 Hz, H3'), 3.07 (d, J_{HH} = 14.5 Hz, H3), 2.87 (d, J_{HH} = 14.5 Hz, H3'), 2.74 (H1'), 2.64 (H3+H1), 2.24 (s, H5/H5'), 2.18 (s, H5/H5'), 1.86 (s, H2'), 1.77 (H1'), 1.69 (Bn), 1.49 (H1), 1.29 (s, H2), 0.90 (Bn), -0.17 (s, Me_A), -2.78 (s, Me_B).<br>^{13}C-NMR (C_6D_5Cl, 233K, δ in ppm): δ = 129.3(C_{ipso}), 68.7 (s, Bn), 64.2 (s, C3), 62.5 (s, C3'), 54.5 (s, C1), 51.9 (s, C1'), 47.6 (s, C2), 46.3 (s, C2'), 20.4 (s, C5/C5'), 20.2 (s, C5/C5'), -3.1 (s, Me_A), -15.0 (s, Me_B).

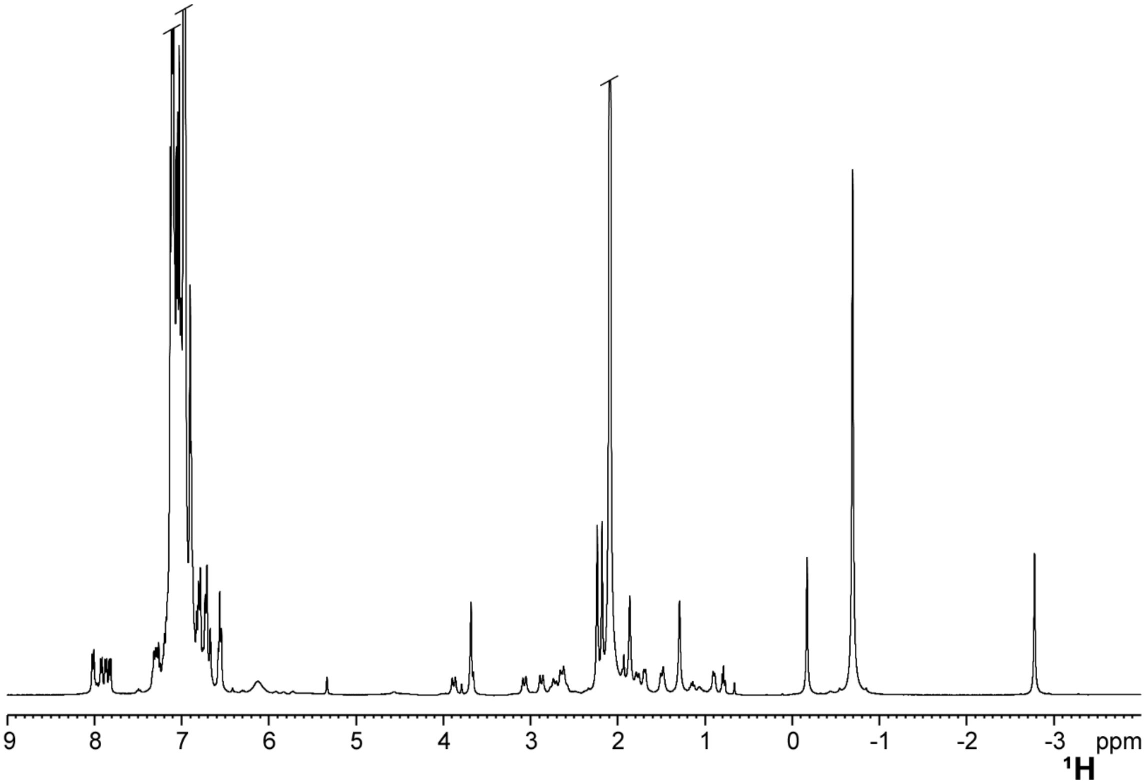

Figure S29. ^{1}H NMR spectrum of **4Hf$_d$** (C_6D_5Cl, 233 K).

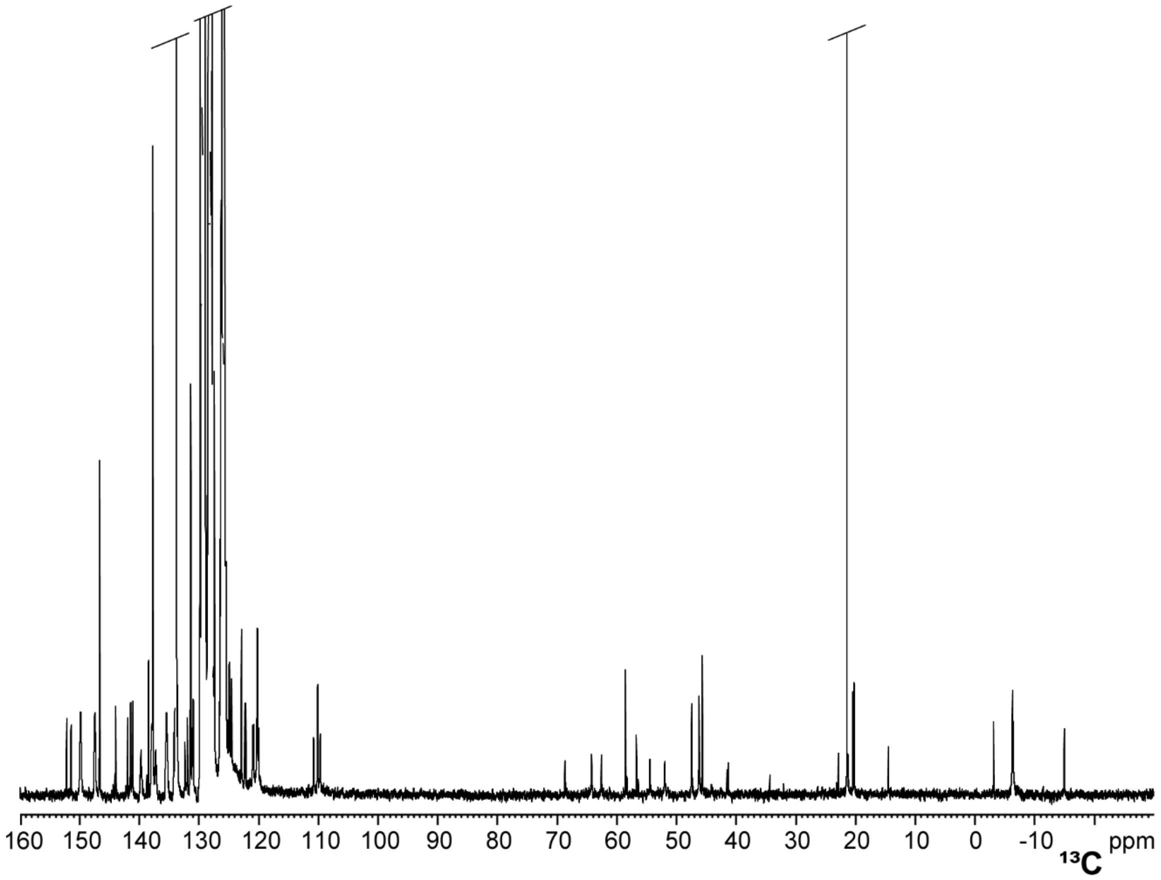

Figure S30. ^{13}C NMR spectrum of **$4Hf_d$** (C_6D_5Cl, 233 K).

Kinetic studies of exchange processes

Variable temperature ^1H EXSY NMR measurements were acquired using the standard "noesygptp" pulse sequence available in the Bruker pulse program library. The relaxation delay was set to 1 s and mixing time values (τ_m) was adjusted between 2.7 and 800 ms, depending on the rate of chemical exchange at each given temperature. Typically, a matrix of 512x512 data points was used for acquisition and the raw data were processed using zero-filling to 2048 data points in both spectral dimensions. The spectral window and the number of transients were optimized depending on distribution of relevant resonances and the sample concentration.

Values of forward and backward magnetization transfer rate constant (k_{1obs} and $k_{-1obs,}$ both in s^{-1}) were evaluated by the method proposed by Perrin,[5] and were calculated from the integration of the 2D spectra by using the EXSYCALC software.[6] For intramolecular processes, such as Site Epimerization (SE) and exchange between different methyl moieties belonging to heterobimetallic adducts, magnetization transfer rate constant values (k_{obs}) correspond to k_{SE}, $k_{Me(A)\text{-}Me(B)}$ or $k_{Me(A)\text{-}Me(C)}$; the corresponding values reported in the following tables are the mathematical average of the experimental forward and backward magnetization transfer rate constants. For intermolecular processes involving exchange of methyl groups between the heterobimetallic adducts and "free" $ZnMe_2$, k_{-1obs} values obtained from EXSYCALC software were multiplied by 2 to account for the number of equivalent methyl groups in $ZnMe_2$. For associative processes, the corresponding forward and backward macroscopic kinetic rate constants were computed as $k_1 = k_{1obs}/[ZnMe_2]$ and $k_{-1} = k_{-1obs}/[Hf]$. The concentrations of the species at equilibrium were estimated by quantitative ^1H NMR spectra using an external standard. Activation parameters of dynamical motions were estimated from the corresponding Eyring plots; errors on activation enthalpy and activation entropy were determined from the quality of linear fitting and computed at 95% confidence interval.

Table S1. Rate constants (k_{SE}, s^{-1}) at different temperatures (T, K) and corresponding activation parameters ($\Delta H^‡$, $kcal \cdot mol^{-1}$; $\Delta S^‡$, $cal \cdot mol^{-1} \cdot K^{-1}$; $\Delta G^‡_{(298)}$, $kcal \cdot mol^{-1}$) for the Site Epimerization process of complexes **$2Hf_a$-$2Hf_d$** in C_6D_5Cl.

**T (K)**	**k_{SE} (s^{-1})**			
	**$2Hf_d$**	**$2Hf_b$**	**$2Hf_c$**	**$2Hf_a$**
223	0.05		0.09	
228	0.17		0.20	
233	0.57		0.62	
238	0.82		1.17	
243	3.06	0.07	2.63	
248	6.82		4.55	
253		0.23	10.22	
258			16.80	
263			25.66	
268		1.12		
273		1.61		0.12
283		5.59		0.26
288		6.27		
303				1.54
308				2.83
313				4.43
318				6.46
**$\Delta H^‡$**	20.7 ± 3.3	13.7 ± 1.3	16.3 ± 1.1	15.0 ± 1.5
**$\Delta S^‡$**	29 ± 14	-7 ±5	10 ± 5	-8 ± 5
**$\Delta G^‡_{298}$**	12.0 ± 5.3	15.8 ± 2.0	13.2 ± 1.7	17.4 ± 2.2

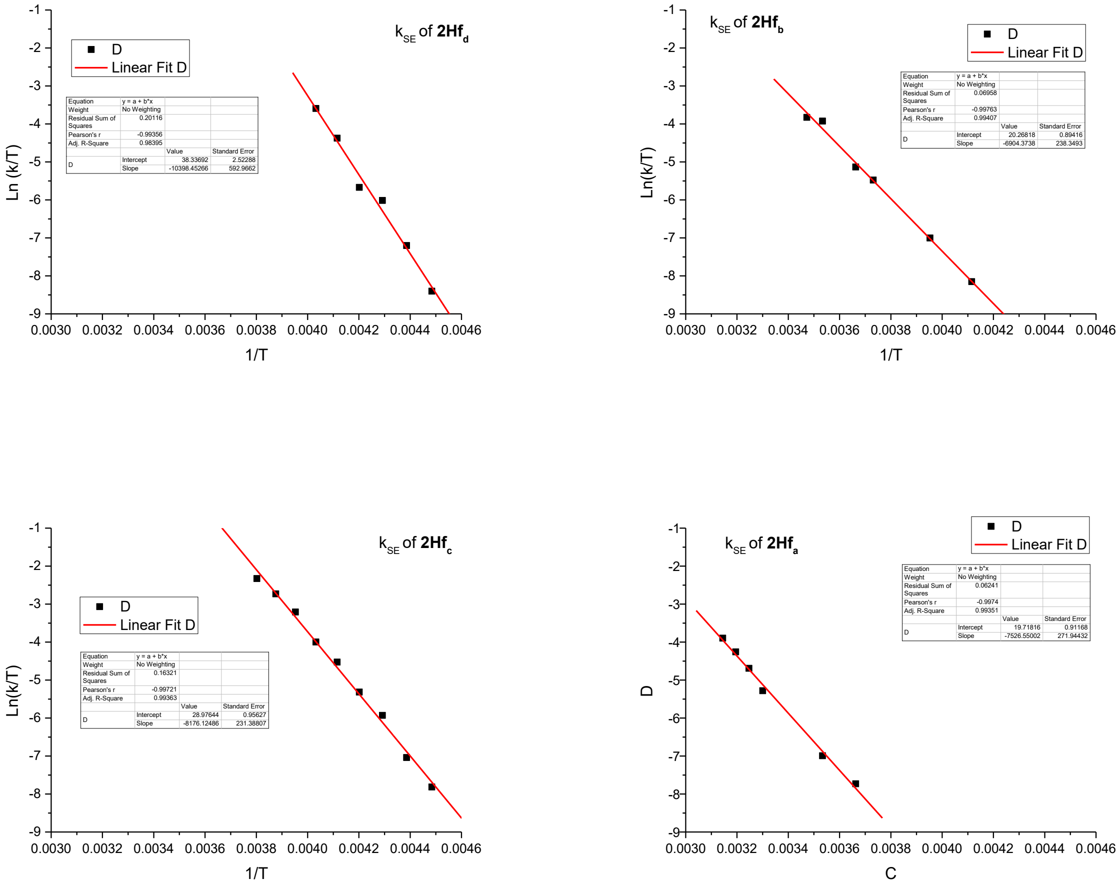

Figure S31. Eyring plots for the Site Epimerization exchange process of complexes **$2Hf_a$-$2Hf_d$** in C_6D_5Cl.

Table S2. Magnetization rate constants (k, s^{-1}) at different temperatures (T, K) and corresponding activation parameters (ΔH‡, kcal·mol^{-1}; ΔS‡, cal·mol^{-1}·K^{-1}; $\Delta G^{\ddagger}_{(298)}$, kcal·$mol^{-1}$) of the Me_A-Me_B exchange process for complexes **$3Hf_a$-$3Hf_d$** and **$3Zr_c$** in C_6D_5Cl.

**T (K)**	**k (s^{-1})**				
	**$3Hf_d$**	**$3Hf_b$**	**$3Hf_c$**	**$3Hf_a$**	**$3Zr_c$**
233		0.16			0.15
238		0.31			0.33
243	0.17	0.60		0.35	0.58
248				0.69	1.23
253	0.60	1.62		1.59	2.31
258			0.08	2.76	4.17
263	1.97	6.46	0.15	4.64	7.00
268			0.27	7.86	10.70
273	5.92	19.19	0.46		
283	16.17	45.58	1.55		
293			3.86		
298			5.90		
**ΔH‡**	15.1 ± 0.1	14.5 ± 0.8	16.0 ± 0.5	15.7 ± 1.5	14.8 ± 0.7
**ΔS‡**	0.4 ± 0.4	0 ± 3	-1 ± 2	4 ± 6	1.8 ± 2.7
**$\Delta G^{\ddagger}_{298}$**	15.0 ± 0.2	14.4 ± 1.2	16.4 ± 0.8	14.4 ± 2.4	14.3 ± 1.0

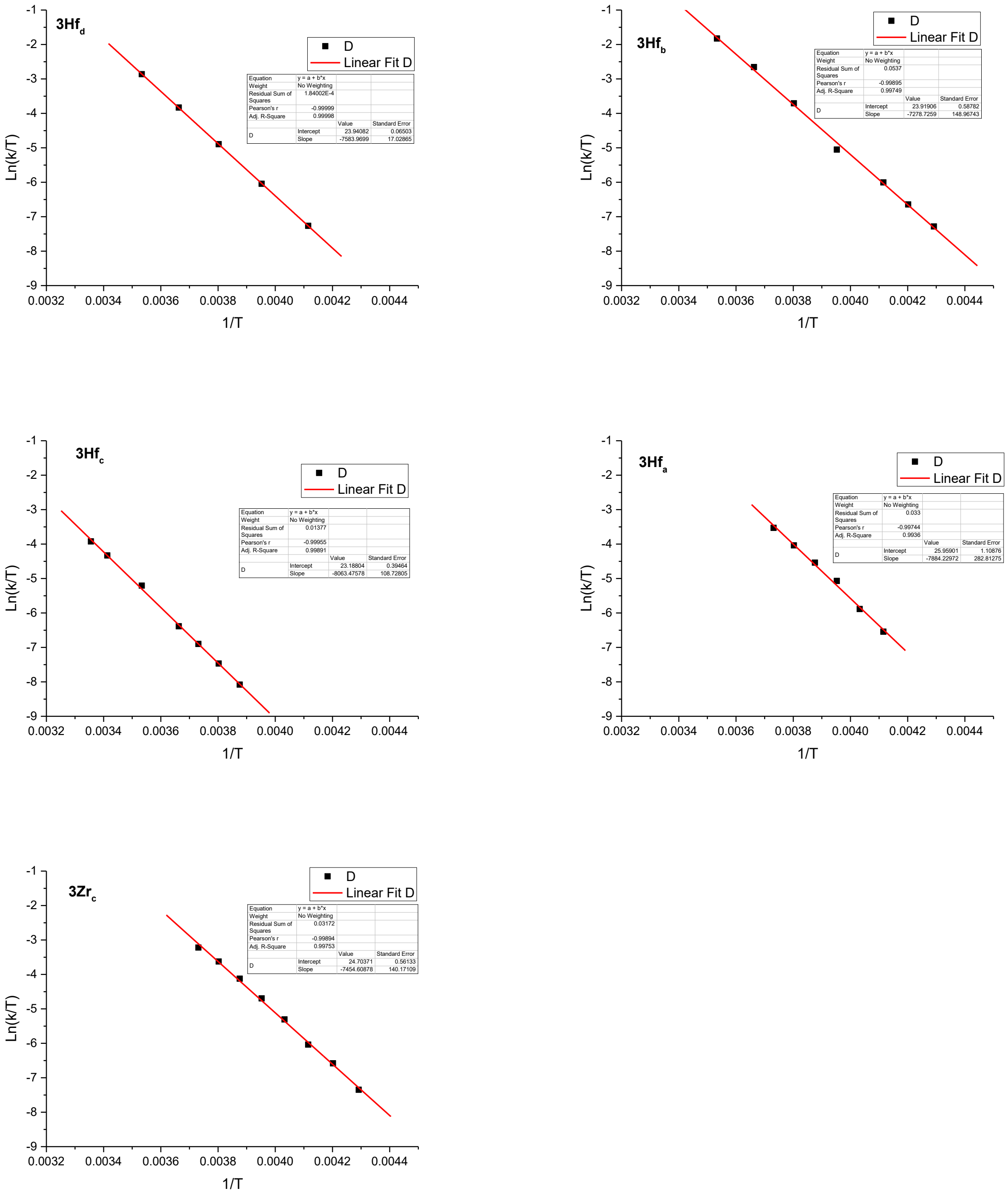

Figure S32. Eyring plots for the Me_A-Me_B exchange process of complexes **3Hf$_a$-3Hf$_d$** and **3Zr$_c$** in C_6D_5Cl.

Table S3. Magnetization rate constant (k, s^{-1}) at different temperatures and corresponding activation parameters ($\Delta H^‡$, kcal·mol^{-1}; $\Delta S^‡$, cal·mol^{-1}·K^{-1}; $\Delta G^‡_{(298)}$, kcal·mol^{-1}) of the Me_A-Me_C exchange process for complexes **3Hf$_a$-3Hf$_d$** in C_6D_5Cl.

**T (K)**	**k (s^{-1})**			
	**3Hf$_d$**	**3Hf$_b$**	**3Hf$_c$**	**3Hf$_a$**
238				0.10
243	0.04	0.35		0.19
248				0.40
253	0.11	1.39		0.90
258				1.77
263	0.43	4.68	0.13	3.53
268			0.24	5.67
273	1.62	13.31	0.41	
283	2.98	33.91	1.38	
293			3.77	
298			5.57	
**$\Delta H^‡$**	15.0 ± 2.8	15.1 ± 1.0	16.4 ± 0.8	17.1 ± 0.9
**$\Delta S^‡$**	-3 ± 11	2 ± 4	0 ± 3	9 ± 4
**$\Delta G^‡_{298}$**	15.9 ± 4.3	14.5 ± 1.5	16.4 ± 1.2	14.4 ± 1.4

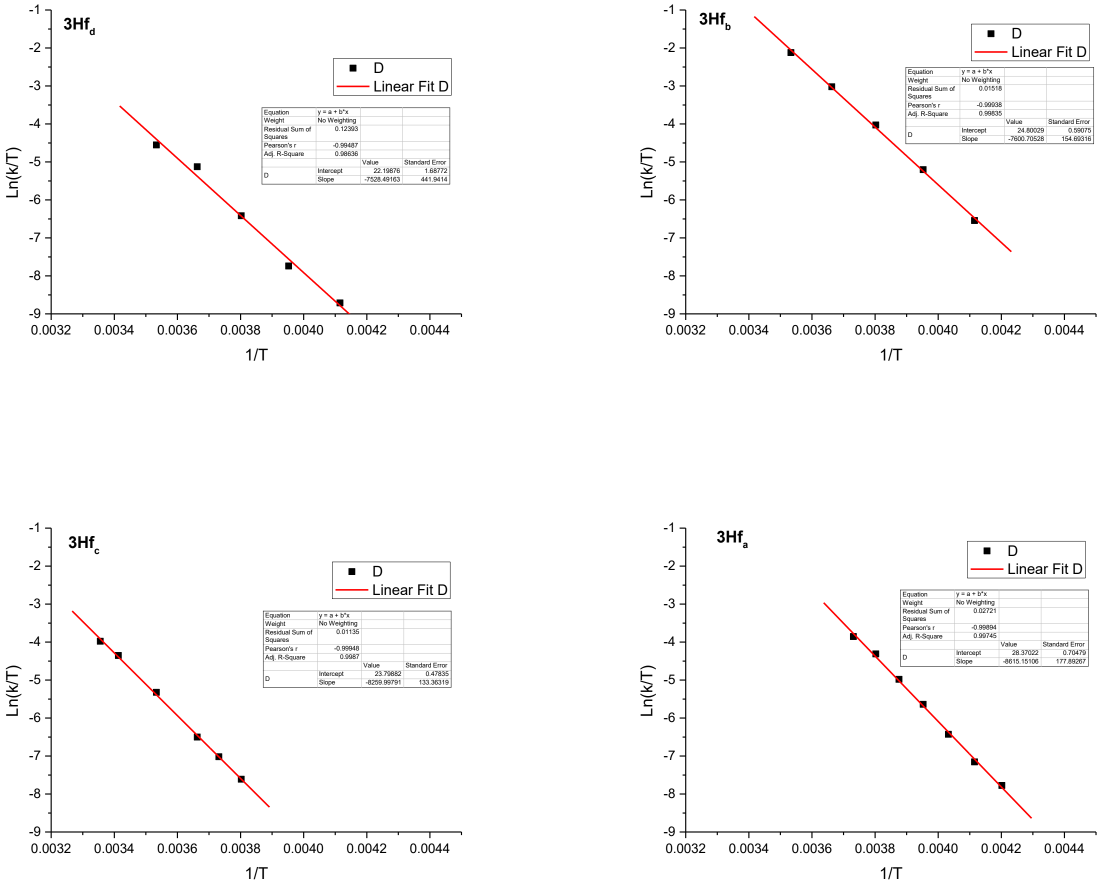

Figure S33. Eyring plots for the Me_A-Me_C exchange process of complexes **$3Hf_a$-$3Hf_d$** in C_6D_5Cl.

Table S4. Magnetization rate constants (k, s^{-1}) at different temperatures (T, K) and corresponding activation parameters ($\Delta H^‡$, kcal·mol^{-1}; $\Delta S^‡$, cal·mol^{-1}·K^{-1}; $\Delta G^‡_{(298)}$, kcal·mol^{-1}) of the Me_C-Me_D exchange process for complexes **$3Hf_d$** in C_6D_5Cl.

T (K)	k (s^{-1})
	**$3Hf_d$**
253	0.32
263	1.14
273	3.39
283	9.65
**$\Delta H^‡$**	15.6 ± 0.8
**$\Delta S^‡$**	1 ± 3
**$\Delta G^‡_{298}$**	15.2 ± 1.2

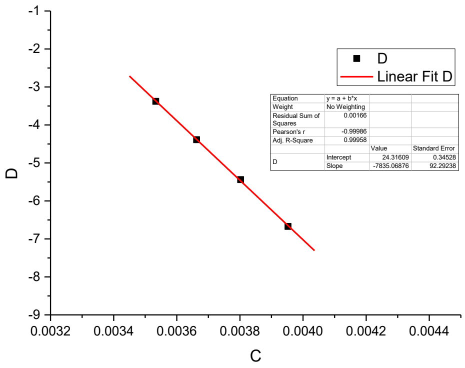

Figure S34. Eyring plots for the Me_C-Me_D exchange process of complexes **$3Hf_d$** in C_6D_5Cl.

Table S5. Rate constants (k_{SE}, s^{-1}) at different temperatures and corresponding activation parameters ($\Delta H^{\ddagger}$, $kcal \cdot mol^{-1}$; $\Delta S^{\ddagger}$, $cal \cdot mol^{-1} \cdot K^{-1}$; $\Delta G^{\ddagger}_{(298)}$, $kcal \cdot mol^{-1}$) for the Site Epimerization process (measured by following Me2/Me2' chemical exchange) of **$3Hf_d$** in C_6D_5Cl.

**T (K)**	**k_{SE} (s^{-1})**
253	0.10
263	0.57
273	1.58
283	5.30
293	19.22
**$\Delta H^{\ddagger}$**	18.3 ± 2.3
**$\Delta S^{\ddagger}$**	10 ± 9
**$\Delta G^{\ddagger}_{298}$**	15.4 ± 3.5

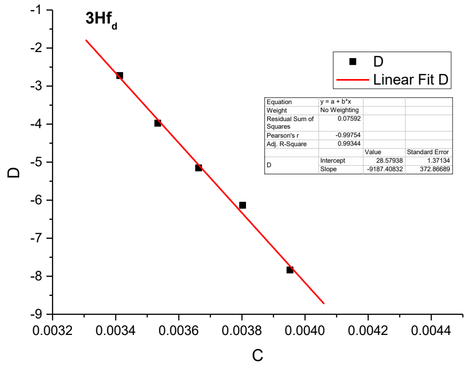

Figure S35. Eyring plots for the Site Epimerization exchange process of complexes **$3Hf_d$** in C_6D_5Cl.

Table S6. Magnetization rate constant (k, s^{-1}) at different temperatures and corresponding activation parameters ($\Delta H^{\ddagger}$, kcal·mol^{-1}; $\Delta S^{\ddagger}$, cal·mol^{-1}·K^{-1}; $\Delta G^{\ddagger}_{(298)}$, kcal·mol$_{-1}$) of the Me_A-Me_B chemical exchange process (*[$ZnMe_2$] = 0.034M, [Hf] = 0.014 M; ** [$ZnMe_2$] = 0.043 M, [Hf] = 0.016 M).

**T (K)**	**k(s^{-1})**	
	**4Hf$_d$ ***	**4Hf$_d$ ****
218	0.09	
223	0.14	
228	0.29	0.28
233	0.60	0.57
238	1.15	1.23
243	2.41	2.50
248	3.91	4.18
253	8.40	8.25
258	12.60	13.8
263	22.00	23.9
**$\Delta H^{\ddagger}$**	13.9 ± 0.6	14.7 ± 0.5
**$\Delta S^{\ddagger}$**	1 ± 3	4.0 ± 2
**$\Delta G^{\ddagger}_{298}$**	13.7 ± 1.0	13.5 ± 0.8

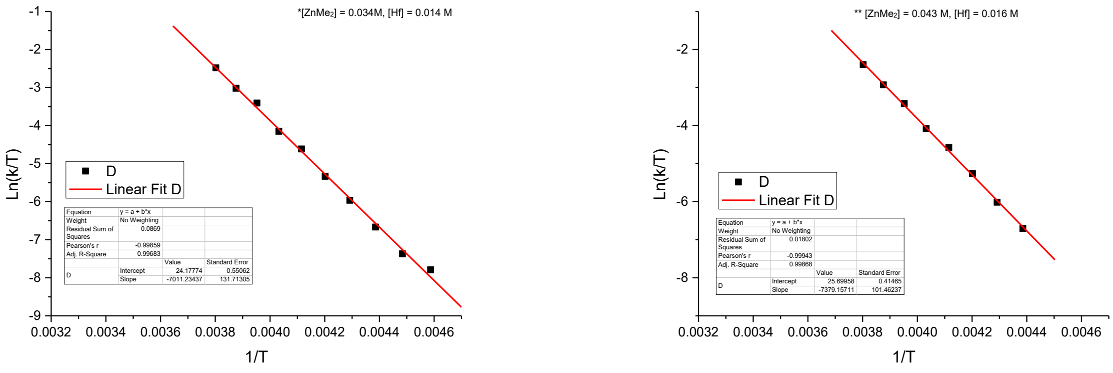

Figure S36. Eyring plots for the Me_A-Me_B exchange process of complexes **4Hf$_d$** in C_6D_5Cl.

Table S7. Magnetization rate constants (k_{1obs} and $k_{-1\ obs}$, s^{-1}) and corresponding second-order rate constant at different temperatures (k_1, k_{-1} and $k_{average}$, $M^{-1}s^{-1}$) of the Me_A-$ZnMe_2$ chemical exchange process of **$4Hf_d$** ([$ZnMe_2$] = 0.034 M, [Hf] = 0.014 M) in C_6D_5Cl.

**T**	**k_{1obs} (s^{-1})**	**k_{-1obs} (s^{-1})**	**k_1 ($M^{-1}s^{-1}$)**	**k_{-1} ($M^{-1}s^{-1}$)**	**$k_{average}$ ($M^{-1}s^{-1}$)**
218	0.72	0.34	21.2	24.3	22.7
223	1.06	0.46	31.2	32.8	32.0
228	1.70	0.77	50.0	55.0	52.5
233	2.64	1.12	77.6	80.0	78.8
238	3.74	1.57	110.0	112.1	111.1
243	6.32	2.36	185.9	168.6	177.2
248	8.29	3.40	243.8	242.9	243.3
253	16.83	5.48	495.0	391.4	443.2
258	25.20	7.56	741.2	540.0	640.6
263	59.30	18.32	1744.1	1308.6	1526.3

Table S8. Magnetization rate constants (k_{1obs} and k_{-1obs}, s^{-1}) at different temperatures of the chemical exchange process Me_B-$ZnMe_2$ of **$4Hf_d$** ([$ZnMe_2$] = 0.034 M, [Hf] = 0.014 M) in C_6D_5Cl.

**T**	**k_{1obs} (s^{-1})**	**k_{-1obs} (s^{-1})**
218	0.015	0.002
223	0.027	0.012
228	0.095	0.042
233	0.244	0.096
238	0.558	0.30
243	1.362	0.58
248	3.621	1.37
253	6.902	2.48
258	15.38	4.84
263	25.38	6.84

Table S9. Forward magnetization rate constants (**k_{1obs}**, s^{-1}) at 233K of the chemical exchange processes of **$4Hf_d$** in C_6D_5Cl as a function of concentrations (M).

**[Hf]**	**[$ZnMe_2$]**	Me_A-Me_B	Me_A-$ZnMe_2$	Me_B-$ZnMe_2$
0.0036	0.018	0.71	1.63	0.56
0.014	0.034	0.60	2.64	0.24
0.016	0.043	0.57	3.37	0.24

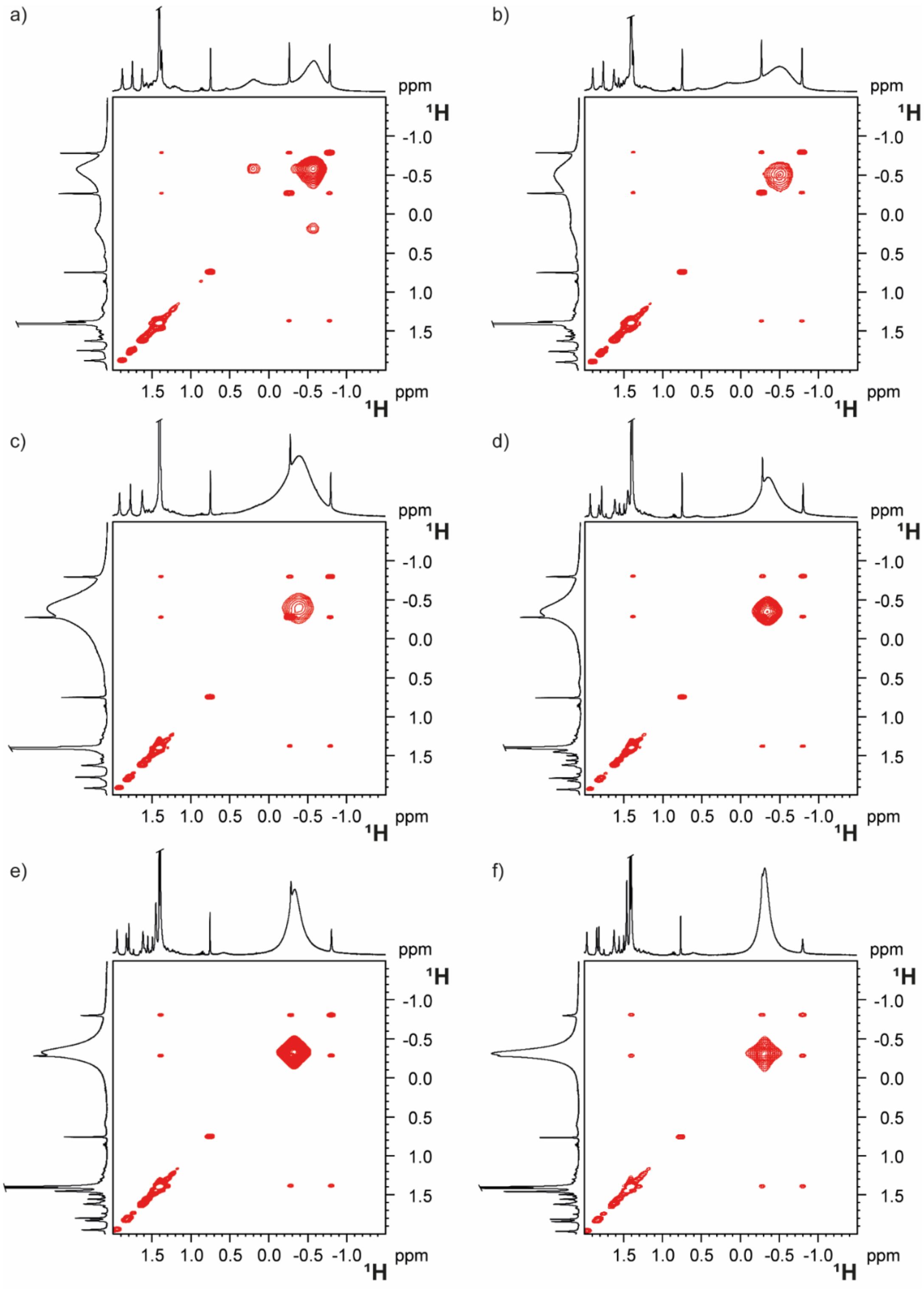

Figure S37. Sections of ^{1}H EXSY NMR spectra for **$3Hf_a$** at different temperatures. a) 243 K; b) 248 K; c) 253 K; d) 258 K; e) 263 K; f) 268 K.

Computational details.

Computational Results for Different Bimetallic Complexes

PCM = Toluene

Name	Formula	Energy(DZ)	Energy(TZ+PCM)	D0	Energy(TZ+PCM+D0)	ZPE	EnthalpyCorr (p=1.0, t=298.15)	EntropyCorr (p=1.0, t=298.15)	E	H	G	E	H	G	ΔH	ΔG
Al2Me6_C2v	C6H18Al2	-724.4418329	-724.5584123	-0.01546691	-724.5738792	0.21415	0.232100389	0.059881501	-724.35973	-724.341779	-724.3818995	-362.1798648	-362.1708894	-362.1909497	-10.2	-4.2
AlMe3_C1	C3H9Al	-362.2076006	-362.2662235	-0.00306352	-362.269287	0.10505	0.114691775	0.044276463	-362.164237	-362.154595	-362.1842605	-362.1642371	-362.1545953	-362.1842605		
**ONNO_otBu_HfBn+_TMA**																
ONNO_otBu_HfBn+_eta2_Al2Me6	C39H63Al2HfN2O2	-2317.857293	-2318.389291	-0.14696833	-2318.536259	0.929827	0.987984313	0.148358776	-2317.60643	-2317.54828	-2317.647675	-2317.606432	-2317.548275	-2317.647675	0.0	0.0
ONNO_otBu_HfBn+_alpha_agostic_Al2Me6	C39H63Al2HfN2O2	-2317.85254	-2318.392453	-0.13868184	-2318.531135	0.927556	0.986386413	0.151599691	-2317.60358	-2317.54475	-2317.64632	-2317.603579	-2317.544748	-2317.64632	2.2	0.9
ONNO_otBu_HfBn+_alpha_agostic_AlMe3_bridged	C36H54AlHfN2O2	-1955.609676	-1956.089804	-0.12633095	-1956.216135	0.819757	0.869185515	0.129229521	-1955.39638	-1955.34695	-1955.433533	-2317.576242	-2317.517839	-2317.624483	19.1	14.6
ONNO_otBu_HfBn+_alpha_agostic_AlMe3_endon	C36H54AlHfN2O2	-1955.614035	-1956.09455	-0.12130339	-1956.215854	0.818419	0.86885601	0.136565209	-1955.39743	-1955.347	-1955.438496	-2317.577299	-2317.517887	-2317.629446	19.1	11.4
ONNO_otBu_HfBn+_alpha_agostic_AlMe3_endonO	C36H54AlHfN2O2	-1955.601451	-1956.07948	-0.12949558	-1956.208976	0.820694	0.869328279	0.12610534	-1955.38828	-1955.33965	-1955.424138	-2317.568146	-2317.510537	-2317.615088	23.7	20.4
**ONNO_otBu_HfMe+_TMA**																
ONNO_otBu_HfMe+_Al2Me6	C33H59Al2HfN2O2	-2086.760535	-2087.245925	-0.12199749	-2087.367923	0.845642	0.899338871	0.138703893	-2086.52228	-2086.46858	-2086.561515	-2086.522281	-2086.468584	-2086.561515	1.2	4.5
ONNO_otBu_HfMe+_AlMe3_endonO	C30H50AlHfN2O2	-1724.509976	-1724.937136	-0.1137474	-1725.050883	0.739013	0.783277917	0.115551149	-1724.31187	-1724.26761	-1724.345025	-2086.491735	-2086.438495	-2086.535974	20.1	20.6
ONNO_otBu_HfMe+_AlMe3_endon	C30H50AlHfN2O2	-1724.522592	-1724.951847	-0.10579041	-1725.057638	0.736735	0.782824111	0.125897477	-1724.3209	-1724.27481	-1724.359165	-2086.500767	-2086.445703	-2086.550115	15.5	11.7
ONNO_otBu_HfMe+_AlMe3_bridged	C30H50AlHfN2O2	-1724.535282	-1724.965244	-0.11337482	-1725.078619	0.738468	0.783105041	0.1163922	-1724.34015	-1724.29551	-1724.373497	-2086.520016	-2086.466403	-2086.564446	2.6	2.7
ONNO_otBu_HfMe+_AlMe3_endonO_B	C30H50AlHfN2O2	-1724.538429	-1724.966075	-0.11626432	-1725.082339	0.738009	0.782752812	0.116711845	-1724.34433	-1724.29959	-1724.377783	-2086.524195	-2086.470476	-2086.568733	0.0	0.0
**ONNO_oCarbazolyl_HfMe+_TMA**																
ONNO_oCarbazolyl_HfMe+_Al2Me6	C49H57Al2HfN4O2	-2804.939797	-2805.570075	-0.14235478	-2805.71243	0.934606	0.997004794	0.161842124	-2804.77782	-2804.71543	-2804.823859	-2804.777824	-2804.715425	-2804.823859	1.2	4.0
ONNO_oCarbazolyl_HfMe+_AlMe3_endonO	C46H48AlHfN4O2	-2442.717205	-2443.286604	-0.13553984	-2443.422143	0.827515	0.879905274	0.137143464	-2442.59463	-2442.54224	-2442.634124	-2804.774493	-2804.713128	-2804.825074	2.7	3.3
ONNO_oCarbazolyl_HfMe+_AlMe3_endon	C46H48AlHfN4O2	-2442.716828	-2443.288365	-0.13105687	-2443.419421	0.827087	0.879843917	0.138947349	-2442.59233	-2442.53958	-2442.632672	-2804.772199	-2804.710467	-2804.823622	4.4	4.2
B_ONNO_HfMe_oCarbazolyl+_C2_AlMe3_endon_to_bridged_TS2_freq	C46H48AlHfN4O2	-2442.699521	-2443.269631	-0.13817523	-2443.407806	0.828033	0.879243248	0.133545761	-2442.57977	-2442.52856	-2442.618039	-2804.759638	-2804.699452	-2804.808988	11.3	13.4
B_ONNO_HfMe_oCarbazolyl+_C2_AlMe3_endonO_rotation_TSfreq	C46H48AlHfN4O2	-2442.698054	-2443.273712	-0.13518581	-2443.408897	0.829533	0.880311538	0.132956508	-2442.57936	-2442.52859	-2442.617667	-2804.759229	-2804.699475	-2804.808616	11.3	13.6
ONNO_oCarbazolyl_HfMe+_AlMe3_bridged	C46H48AlHfN4O2	-2442.711769	-2443.286489	-0.13129536	-2443.417785	0.826951	0.879737618	0.139391911	-2442.59083	-2442.53805	-2442.63144	-2804.770698	-2804.708937	-2804.82239	5.3	5.0
ONNO_oCarbazolyl_HfMe+_AlMe3_endonO_B	C46H48AlHfN4O2	-2442.717698	-2443.290419	-0.13580861	-2443.426227	0.827075	0.879710532	0.138548276	-2442.59915	-2442.54652	-2442.639344	-2804.779017	-2804.717406	-2804.830294	0.0	0.0
**ONNO_oCarbazolyl_HfBn+_ZnMe2**																
ONNO_oCarbazolyl_HfBn+_alpha_agostic_ZnMe2_endonO	C51H49HfN4O2Zn	-2618.431685	-2619.039371	-0.15327831	-2619.192649	0.872361	0.927311114	0.14465742	-2618.32029	-2618.26534	-2618.362259	-2618.320289	-2618.265338	-2618.362259	14.6	14.8
ONNO_oCarbazolyl_HfBn+_alpha_agostic_ZnMe2_endonO_B	C51H49HfN4O2Zn	-2618.430343	-2619.042349	-0.15464007	-2619.196989	0.872304	0.927420188	0.146220555	-2618.32469	-2618.26957	-2618.367537	-2618.324685	-2618.269569	-2618.367537	11.9	11.5
ONNO_oCarbazolyl_HfBn+_alpha_agostic_ZnMe2_endon	C51H49HfN4O2Zn	-2618.438376	-2619.052918	-0.14834448	-2619.201263	0.872506	0.92778271	0.148143781	-2618.32876	-2618.27348	-2618.372737	-2618.328757	-2618.27348	-2618.372737	9.5	8.2
ONNO_oCarbazolyl_HfBn+_eta2_ZnMe2_endon	C51H49HfN4O2Zn	-2618.446906	-2619.061856	-0.15597196	-2619.217828	0.87451	0.929227608	0.145098501	-2618.34332	-2618.2886	-2618.385816	-2618.343318	-2618.2886	-2618.385816	0.0	0.0
ONNO_oCarbazolyl_HfBn+_ZnMe2_bridged	C51H49HfN4O2Zn	-2618.435616	-2619.048933	-0.15293758	-2619.201871	0.872228	0.927473166	0.146677295	-2618.32964	-2618.2744	-2618.372671	-2618.329642	-2618.274397	-2618.372671	8.9	8.2
ONNO_oCarbazolyl_HfBn+_eta2_ZnMe2_endonO	C51H49HfN4O2Zn	-2618.445755	-2619.052658	-0.16107708	-2619.213735	0.87442	0.928756276	0.14180901	-2618.33931	-2618.28498	-2618.379991	-2618.339315	-2618.284979	-2618.379991	2.3	3.7
ONNO_oCarbazolyl_HfBn+_alpha_agostic_ZnMe2_endonON	C51H49HfN4O2Zn	-2618.429449	-2619.039175	-0.15189782	-2619.191073	0.871203	0.92682528	0.147254397	-2618.31987	-2618.26425	-2618.362908	-2618.319869	-2618.264247	-2618.362908	15.3	14.4
ONNO_oCarbazolyl_HfBn+_alpha_agostic_ZnMe2_endonON_B	C51H49HfN4O2Zn	-2618.438265	-2619.045573	-0.15822178	-2619.203795	0.874547	0.928759707	0.14201388	-2618.32925	-2618.27504	-2618.370184	-2618.329248	-2618.275035	-2618.370184	8.5	9.8

PCM = Chlorobenzene

Name	Formula	Energy(DZ)	Energy(TZ+PCM)	D0	Energy(TZ+PCM+D0)	ZPE	EnthalpyCorr (p=1.0, t=298.15)	EntropyCorr (p=1.0, t=298.15)	E	H	G	E	H	G	ΔH	ΔG
Al2Me6_C2v	C6H18Al2	-724.4418329	-724.5592702	-0.01546691	-724.5747371	0.21415	0.232100389	0.059881501	-724.360587	-724.342637	-724.3827573	-362.1802937	-362.1713184	-362.1913787	-10.0	-4.0
AlMe3_C1	C3H9Al	-362.2076006	-362.2669361	-0.00306352	-362.2699996	0.10505	0.114691775	0.044276463	-362.16495	-362.155308	-362.184973	-362.1649497	-362.1553078	-362.184973		
**ONNO_otBu_HfBn+_TMA**																
ONNO_otBu_HfBn+_eta2_Al2Me6	C39H63Al2HfN2O2	-2317.857293	-2318.405877	-0.14696833	-2318.552846	0.929827	0.987984313	0.148358776	-2317.62302	-2317.56486	-2317.664262	-2317.623019	-2317.564861	-2317.664262	0.0	0.0
ONNO_otBu_HfBn+_alpha_agostic_Al2Me6	C39H63Al2HfN2O2	-2317.85254	-2318.403706	-0.13868184	-2318.542387	0.927556	0.986386413	0.151599691	-2317.61483	-2317.556	-2317.657573	-2317.614832	-2317.556001	-2317.657573	5.6	4.2
ONNO_otBu_HfBn+_alpha_agostic_AlMe3_bridged	C36H54AlHfN2O2	-1955.609676	-1956.104522	-0.12633095	-1956.230853	0.819757	0.869185515	0.129229521	-1955.4111	-1955.36167	-1955.448251	-2317.590961	-2317.532986	-2317.63963	20.0	15.5
ONNO_otBu_HfBn+_alpha_agostic_AlMe3_endon	C36H54AlHfN2O2	-1955.614035	-1956.109778	-0.12130339	-1956.231081	0.818419	0.86885601	0.136565209	-1955.41266	-1955.36223	-1955.453724	-2317.592527	-2317.533544	-2317.645102	19.7	12.0
ONNO_otBu_HfBn+_alpha_agostic_AlMe3_endonO	C36H54AlHfN2O2	-1955.601451	-1956.093396	-0.12949558	-1956.222891	0.820694	0.869328279	0.12610534	-1955.4022	-1955.35356	-1955.438053	-2317.582062	-2317.524881	-2317.629432	25.1	21.9
**ONNO_otBu_HfMe+_TMA**																
ONNO_otBu_HfMe+_Al2Me6	C33H59Al2HfN2O2	-2086.760535	-2087.260178	-0.12199749	-2087.382176	0.845642	0.899338871	0.138703893	-2086.53653	-2086.48284	-2086.575768	-2086.536534	-2086.482837	-2086.575768	1.4	4.7
ONNO_otBu_HfMe+_AlMe3_endonO	C30H50AlHfN2O2	-1724.509976	-1724.951007	-0.1137474	-1725.064754	0.739013	0.783277917	0.115551149	-1724.32574	-1724.28148	-1724.358896	-2086.505606	-2086.452795	-2086.550274	20.2	20.7
ONNO_otBu_HfMe+_AlMe3_endon	C30H50AlHfN2O2	-1724.522592	-1724.966679	-0.10579041	-1725.072469	0.736735	0.782824111	0.125897477	-1724.33573	-1724.28965	-1724.373996	-2086.515599	-2086.460963	-2086.565375	15.1	11.2
ONNO_otBu_HfMe+_AlMe3_bridged	C30H50AlHfN2O2	-1724.535282	-1724.979999	-0.11337482	-1725.093373	0.738468	0.783105041	0.1163922	-1724.35491	-1724.31027	-1724.388251	-2086.53477	-2086.481587	-2086.57963	2.2	2.3
ONNO_otBu_HfMe+_AlMe3_endonO_B	C30H50AlHfN2O2	-1724.538429	-1724.980209	-0.11626432	-1725.096474	0.738009	0.782752812	0.116711845	-1724.35847	-1724.31372	-1724.391918	-2086.53833	-2086.485039	-2086.583297	0.0	0.0
**ONNO_oCarbazolyl_HfMe+_TMA**																
ONNO_oCarbazolyl_HfMe+_Al2Me6	C49H57Al2HfN4O2	-2804.939797	-2805.585859	-0.14235478	-2805.728213	0.934606	0.997004794	0.161842124	-2804.79361	-2804.73121	-2804.839643	-2804.793607	-2804.731209	-2804.839643	1.8	4.6
ONNO_oCarbazolyl_HfMe+_AlMe3_endonO	C46H48AlHfN4O2	-2442.717205	-2443.301973	-0.13553984	-2443.437513	0.827515	0.879905274	0.137143464	-2442.61	-2442.55761	-2442.649493	-2804.789862	-2804.728926	-2804.840872	3.3	3.9
ONNO_oCarbazolyl_HfMe+_AlMe3_endon	C46H48AlHfN4O2	-2442.716828	-2443.303943	-0.13105687	-2443.435	0.827087	0.879843917	0.138947349	-2442.60791	-2442.55516	-2442.648251	-2804.787778	-2804.726475	-2804.83963	4.8	4.6
B_ONNO_HfMe_oCarbazolyl+_C2_AlMe3_endon_to_bridged_TS2_freq	C46H48AlHfN4O2	-2442.699521	-2443.284567	-0.13817523	-2443.422742	0.828033	0.879243248	0.133545761	-2442.59471	-2442.5435	-2442.632975	-2804.774574	-2804.714817	-2804.824353	12.1	14.2
B_ONNO_HfMe_oCarbazolyl+_C2_AlMe3_endonO_rotation_TSfreq	C46H48AlHfN4O2	-2442.698054	-2443.29022	-0.13518581	-2443.425406	0.829533	0.880311538	0.132956508	-2442.59587	-2442.54509	-2442.634175	-2804.775738	-2804.716413	-2804.825554	11.1	13.5
ONNO_oCarbazolyl_HfMe+_AlMe3_bridged	C46H48AlHfN4O2	-2442.711769	-2443.30301	-0.13129536	-2443.434305	0.826951	0.879737618	0.139391911	-2442.60735	-2442.55457	-2442.64796	-2804.787218	-2804.725886	-2804.839339	5.2	4.8
ONNO_oCarbazolyl_HfMe+_AlMe3_endonO_B	C46H48AlHfN4O2	-2442.717698	-2443.306729	-0.13580861	-2443.442537	0.827075	0.879710532	0.138548276	-2442.61546	-2442.56283	-2442.655654	-2804.795327	-2804.734145	-2804.847033	0.0	0.0
**ONNO_oCarbazolyl_HfBn+_ZnMe2**																
ONNO_oCarbazolyl_HfBn+_alpha_agostic_ZnMe2_endonO	C51H49HfN4O2Zn	-2618.431685	-2619.055199	-0.15327831	-2619.208477	0.872361	0.927311114	0.14465742	-2618.33612	-2618.28117	-2618.378087	-2618.336117	-2618.281166	-2618.378087	16.7	16.8
ONNO_oCarbazolyl_HfBn+_alpha_agostic_ZnMe2_endonO_B	C51H49HfN4O2Zn	-2618.430343	-2619.060781	-0.15464007	-2619.215421	0.872304	0.927420188	0.146220555	-2618.34312	-2618.288	-2618.385968	-2618.343117	-2618.288001	-2618.385968	12.4	11.9
ONNO_oCarbazolyl_HfBn+_alpha_agostic_ZnMe2_endon	C51H49HfN4O2Zn	-2618.438376	-2619.071921	-0.14834448	-2619.220266	0.872506	0.92778271	0.148143781	-2618.34776	-2618.29248	-2618.391739	-2618.347759	-2618.292483	-2618.391739	9.6	8.3
ONNO_oCarbazolyl_HfBn+_eta2_ZnMe2_endon	C51H49HfN4O2Zn	-2618.446906	-2619.080975	-0.15597196	-2619.236947	0.87451	0.929227608	0.145098501	-2618.36244	-2618.30772	-2618.404936	-2618.362438	-2618.30772	-2618.404936	0.0	0.0
ONNO_oCarbazolyl_HfBn+_ZnMe2_bridged	C51H49HfN4O2Zn	-2618.435616	-2619.066792	-0.15293758	-2619.219729	0.872228	0.927473166	0.146677295	-2618.3475	-2618.29226	-2618.39053	-2618.347501	-2618.292256	-2618.39053	9.7	9.0
ONNO_oCarbazolyl_HfBn+_eta2_ZnMe2_endonO	C51H49HfN4O2Zn	-2618.445755	-2619.067734	-0.16107708	-2619.228811	0.87442	0.928756276	0.14180901	-2618.35439	-2618.30005	-2618.395067	-2618.354391	-2618.300055	-2618.395067	4.8	6.2
ONNO_oCarbazolyl_HfBn+_alpha_agostic_ZnMe2_endonON	C51H49HfN4O2Zn	-2618.429449	-2619.056449	-0.15189782	-2619.208347	0.871203	0.92682528	0.147254397	-2618.33714	-2618.28152	-2618.380182	-2618.337144	-2618.281522	-2618.380182	16.4	15.5
ONNO_oCarbazolyl_HfBn+_alpha_agostic_ZnMe2_endonON_B	C51H49HfN4O2Zn	-2618.438265	-2619.062202	-0.15822178	-2619.220424	0.874547	0.928759707	0.14201388	-2618.34588	-2618.29166	-2618.386814	-2618.345877	-2618.291664	-2618.386814	10.1	11.4

Relative Gibbs Free Energies and relevant predicted ^{13}C shifts for Systems **$3Hf_a$**, **$3Hf_d$** and **$4Hf_d$** (solvent toluene)

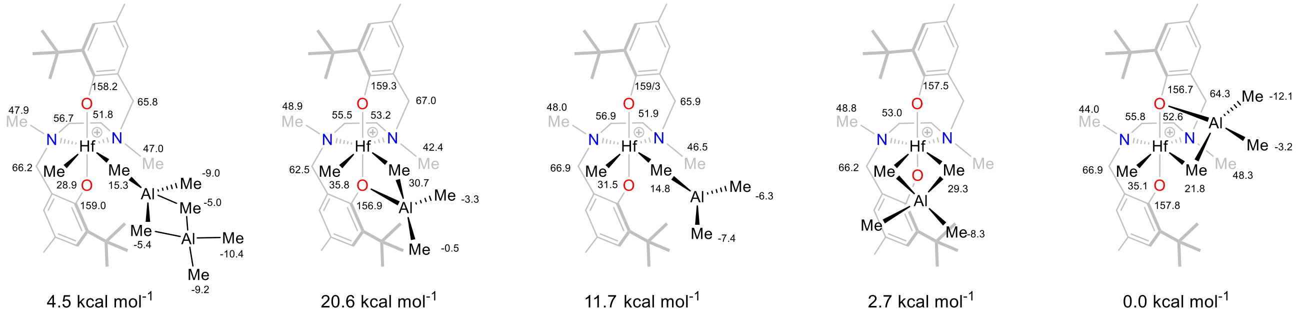

4.5 kcal mol^{-1} | 20.6 kcal mol^{-1} | 11.7 kcal mol^{-1} | 2.7 kcal mol^{-1} | 0.0 kcal mol^{-1}

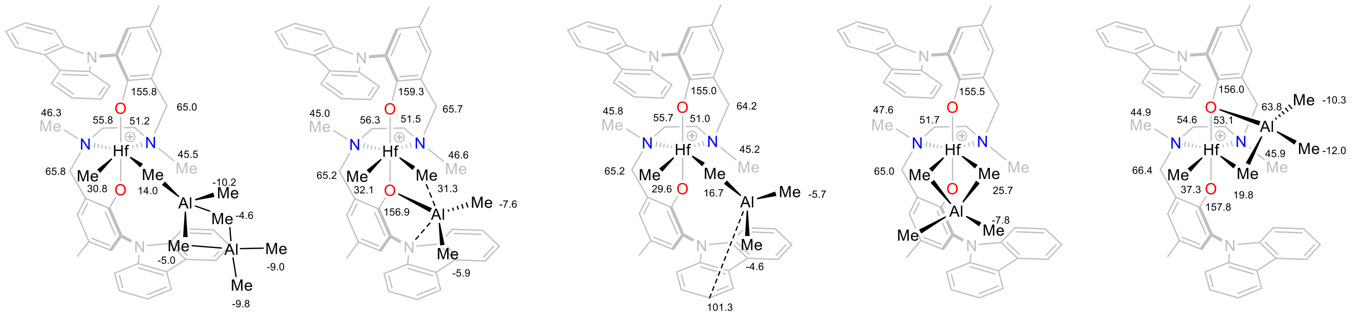

4.0 kcal mol^{-1} | 3.3 kcal mol^{-1} | 4.2 kcal mol^{-1} | 5.0 kcal mol^{-1} | 0.0 kcal mol^{-1}

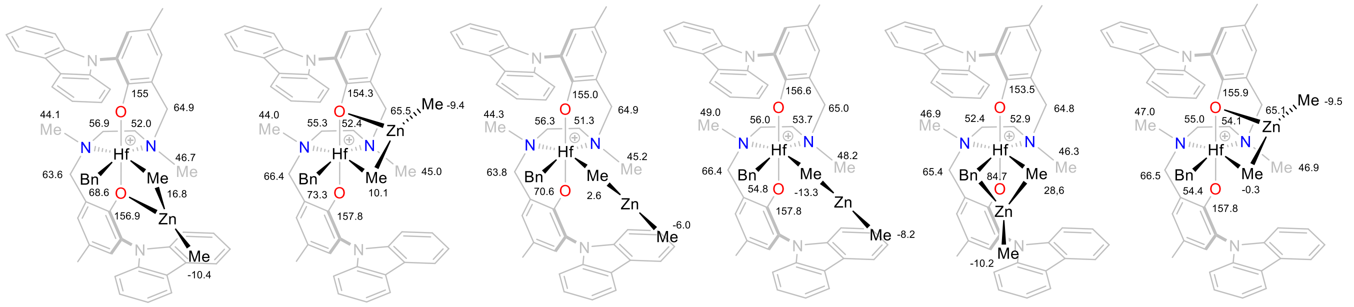

14.8 kcal mol^{-1} | 11.5 kcal mol^{-1} | 8.2 kcal mol^{-1} | 0.0 kcal mol^{-1} η^2-Bn coordination | 8.2 kcal mol^{-1} | 3.7 kcal mol^{-1} η^2-Bn coordination

Relative Gibbs Free Energies and relevant predicted ^{13}C shifts for Systems **3Hf$_a$**, **3Hf$_d$** and **4Hf$_d$** (solvent chlorobenzene)

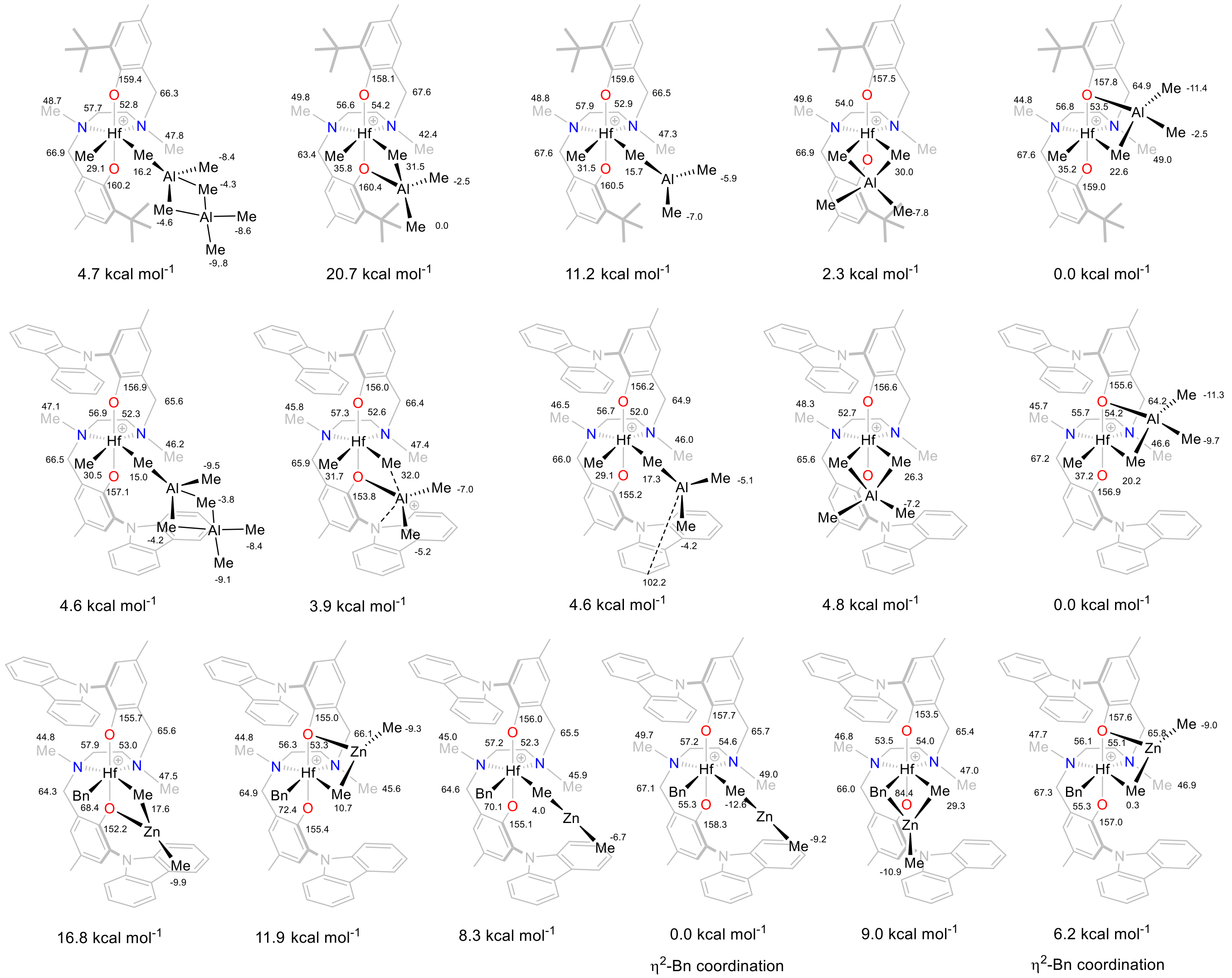

---

[1] Karimpour, T.; Safaei, E.; Wojtczak, A.; Jagličić, Z.; Kozakiewicz, A. Iron(III) complexes of ethylenediamine derivatives of aminophenol ligands as models for enzyme–substrate adducts of catechol dioxygenases. Inorg. Chim. Acta 2013, 395, 124-134.

[2] Busico, V.; Cipullo, R.; Friederichs, N.; Ronca, S.; Talarico, G.; Togrou, M.; Wang, B. Block Copolymers of Highly Isotactic Polypropylene via Controlled Ziegler–Natta Polymerization, Macromolecules 2004, 37, 8201-8203.

[3] (a) Cipullo. R.; Busico, V.; Fraldi, N.; Pellecchia, R.; Talarico, G. Improving the Behavior of Bis(phenoxyamine) Group 4 Metal Catalysts for Controlled Alkene Polymerization, Macromolecules 2009, 42, 3869–3872. (b) Ye, X.; Atienza, C.C.H.; Holtcamp, M.W.; Sanders, D.F.; Day, G.S.; Titone, M.E.; Cano, D.A.; Bedoya, M.S. Int. pat. appl. WO2017058388, 2017. (c) Antinucci, G.; Dereli, B.; Vittoria, A.; Budzelaar, P.H.M.; Cipullo, R.; Goryunov, G.P.; Kulyabin, P.S.; Uborsky, D.V.; Cavallo, L.; Ehm, C.; Voskoboynikov, A.Z.; Busico, V. Selection of Low-Dimensional 3-D Geometric Descriptors for Accurate Enantioselectivity Prediction ACS Catal. 2022, 12, 6934-6945.

[4] A. Dall'Anese, P. S. Kulyabin, D. V. Uborsky, A. Vittoria, C. Ehm, R. Cipullo, P. H. M. Budzelaar, A. Z. Voskoboynikov, V. Busico, L. Tensi, A. Macchioni, C. Zuccaccia, Inorg. Chem., 2023, 62, 16021−16037.

[5] Perrin, C. L.; Dwyer, T. J. Application of Two-Dimensional NMR to Kinetics of Chemical Exchange. Chem. Rev. 1990, 90 (6), 935–967.
[6] https://mestrelab.com/software/freeware/.
